# Bio-crude transcriptomics: Gene discovery and metabolic network reconstruction for the biosynthesis of the terpenome of the hydrocarbon oil-producing green alga, *Botryococcus braunii* race B (Showa)*

**DOI:** 10.1186/1471-2164-13-576

**Published:** 2012-10-30

**Authors:** István Molnár, David Lopez, Jennifer H Wisecaver, Timothy P Devarenne, Taylor L Weiss, Matteo Pellegrini, Jeremiah D Hackett

**Affiliations:** 1 Natural Products Center, School of Natural Resources and the Environment, The University of Arizona, 250 E. Valencia Rd, Tucson, AZ, 85739, USA; 2 Bio5 Institute, The University of Arizona, 1657 E. Helen St, Tucson, AZ, 85721, USA; 3 Department of Molecular, Cell and Developmental Biology, University of California Los Angeles, P. O. Box 951606, Los Angeles, CA, 90095, USA; 4 Department of Ecology and Evolutionary Biology, The University of Arizona, 1041 E. Lowell St, Tucson, AZ, 85721, USA; 5 Department of Biochemistry and Biophysics, Texas A&M University, 2128 TAMU, College Station, TX, 77843, USA

**Keywords:** Biofuel, Terpene biosynthesis, Fatty acid biosynthesis, Triacylglycerol biosynthesis, Starch biosynthesis, ABC transporter, Autophagy, Transcriptome, Botryococcus braunii, Botryococcene

## Abstract

**Background:**

Microalgae hold promise for yielding a biofuel feedstock that is sustainable, carbon-neutral, distributed, and only minimally disruptive for the production of food and feed by traditional agriculture. Amongst oleaginous eukaryotic algae, the B race of *Botryococcus braunii* is unique in that it produces large amounts of liquid hydrocarbons of terpenoid origin. These are comparable to fossil crude oil, and are sequestered outside the cells in a communal extracellular polymeric matrix material. Biosynthetic engineering of terpenoid bio-crude production requires identification of genes and reconstruction of metabolic pathways responsible for production of both hydrocarbons and other metabolites of the alga that compete for photosynthetic carbon and energy.

**Results:**

A *de novo* assembly of 1,334,609 next-generation pyrosequencing reads form the Showa strain of the B race of *B. braunii* yielded a transcriptomic database of 46,422 contigs with an average length of 756 bp. Contigs were annotated with pathway, ontology, and protein domain identifiers. Manual curation allowed the reconstruction of pathways that produce terpenoid liquid hydrocarbons from primary metabolites, and pathways that divert photosynthetic carbon into tetraterpenoid carotenoids, diterpenoids, and the prenyl chains of meroterpenoid quinones and chlorophyll. Inventories of machine-assembled contigs are also presented for reconstructed pathways for the biosynthesis of competing storage compounds including triacylglycerol and starch. Regeneration of *S*-adenosylmethionine, and the extracellular localization of the hydrocarbon oils by active transport and possibly autophagy are also investigated.

**Conclusions:**

The construction of an annotated transcriptomic database, publicly available in a web-based data depository and annotation tool, provides a foundation for metabolic pathway and network reconstruction, and facilitates further omics studies in the absence of a genome sequence for the Showa strain of *B. braunii*, race B. Further, the transcriptome database empowers future biosynthetic engineering approaches for strain improvement and the transfer of desirable traits to heterologous hosts.

## Background

Excessive reliance on fossil hydrocarbons for world energy and synthetic chemistry needs has led to environmental degradation such as global warming, economic imbalances, and their associated national and geopolitical risks for producing and exporting nations alike. The problem is most acute in the liquid fuel sector (itself responsible for about two thirds of the global energy demand, [1]), where renewable sources of energy made the least impact up until now. Microbial conversion of feedstocks (simple sugars, starches and other polysaccharides, and total biomass) to biofuels (primarily ethanol as of now, but preferably other alcohols and fatty acid esters in the future) promises a renewable and potentially globally distributed source of transportation fuel. “Photosynthetic biofuels” directly link the biosynthesis of energy-rich, storable, transportation-friendly, and fuel infrastructure-compatible metabolites to photosynthesis, using land plants, eukaryotic microalgae or cyanobacteria as cell factories to yield a sustainable and potentially carbon-neutral source of fuels. Microalgae may be especially advantageous as they can be grown on marginal or even non-arable land, and may use water sources not directly utilizable in traditional agriculture [2-5]. In addition, microalgae grow faster, have higher photosynthetic productivity [1,5], and accumulate biofuel feedstocks to a much higher percentage of their total biomass than land plants [6,7]. These advantages translate to 15–60 times higher annual oil productivity as compared to soybean, the main US oil crop, grown on the same area of land [2,4,5].

Oleaginous algae accumulate storage carbon and energy in the form of neutral lipids, mainly triacylglycerols (TAG), which need to be extracted from intracellular oil bodies by disrupting the cells. TAG conversion to biofuel crude usually involves transesterification of the constituent fatty acids with alcohols before the resulting bio-crude is refined to transportation fuels [8]. In contrast, the cosmopolitan green colonial microalga *Botryococcus braunii* (Chlorophyta, Trebouxiophyceae) stores photosynthetic carbon in the form of liquid hydrocarbons which need no chemical conversion to provide biofuel crude. This ready-made bio-crude is compatible with, and can be directly processed by the existing petrochemical refinery and distribution infrastructure to yield jet fuel, gasoline, and diesel with little coke formation [9]. Further, this liquid hydrocarbon bio-crude is stored in an extracellular polymeric matrix material surrounding the individual cells of the colony [10]. This may permit relatively mild extraction procedures that use nondisruptive, nontoxic solvents, thus allowing the recycling of “de-fatted” algal biomass (“milking” [1]).

*B. braunii* strains belong to three races defined by the chemical nature of their liquid hydrocarbon products. Race A strains accumulate C_23_-C_33_ alkadienes and alkatrienes derived from very long chain fatty acids (VLCFA) [10,11]. Race B and race L strains produce triterpenoids (C_30_-C_37_ botryococcenes and methylated squalenes) or tetraterpenoids (C_40_ lycopadiene), respectively [10,11]. B race strains of *B. braunii* typically accumulate hydrocarbons at 30-40% of their dry cell weight, although their hydrocarbon content can reach as high as 86% [12]. Hydrocarbon accumulation apparently does not require specific metabolic triggers like nitrogen starvation as seen in TAG-accumulating microalgae [9-11]. Hydrocarbons originating from all races of *B. braunii* have repeatedly been isolated from fossil crude oils and coal deposits in significant amounts, indicating a possible role for this alga in the formation of our current petroleum reserves [9,13] (and references therein).

The genomes of about a dozen photosynthetic algae, including model organisms such as *Chlamydomonas reinhardtii*, *Chlorella variabilis* and *Volvox carteri* have been sequenced and annotated (http://genome.jgi-psf.org) to gain insight into unique aspects of algal biology [14-16]. These investigations also led to the reconstructions of metabolic pathways and networks for selected biological processes of interest, including those that govern lipid production [17-19]. Multiple homologs of enzymes involved in triacylglycerol biosynthesis, such as diacylglcyerol acyltransferases, phospholipid diacylglycerol acyltransferase and phosphatidate phosphatases, have been identified in algae [19,20]. Next-generation sequencing of transcripts from algal cultures grown under various conditions was used to discover regulated genes that are critical in key biochemical processes and biological pathways [21,22]. Next-generation sequencing has also been utilized to assemble the complete transcriptome of the microalgae *Dunaliella tertiolecta* and *Chlorella vulgaris*, and to discover pathways involved in TAG biosynthesis [23,24].

Functional annotation, the process of assigning biological meaning to genomic and transcriptomic data, is an important step in extracting useful information from genomic and transcriptomic projects. The annotation process involves assigning biological pathway, ontology, and protein domain data to genes and transcripts. In the case of *de novo* assembly of genomes and transcriptomes, sequence similarity is a common basis for assigning annotations [23]. Sequences from primary annotation databases, such as KEGG [25], Gene Ontology [26], and Pfam [27], can be used to assign biological function to the assembled transcripts, and further manual curation can be used to validate and extend the annotations derived from sequence similarity. To provide the most utility from a collection of functional annotations, data mining environments are commonly used to facilitate the analysis of large-scale datasets and to provide a public repository of functional data. Several such tools are available for algae, including the Algal Functional Annotation Tool, which allows visualization of sets of transcripts on pathway maps and provides for statistically rigorous functional analysis of large sets of transcripts [28].

**Figure 1:**
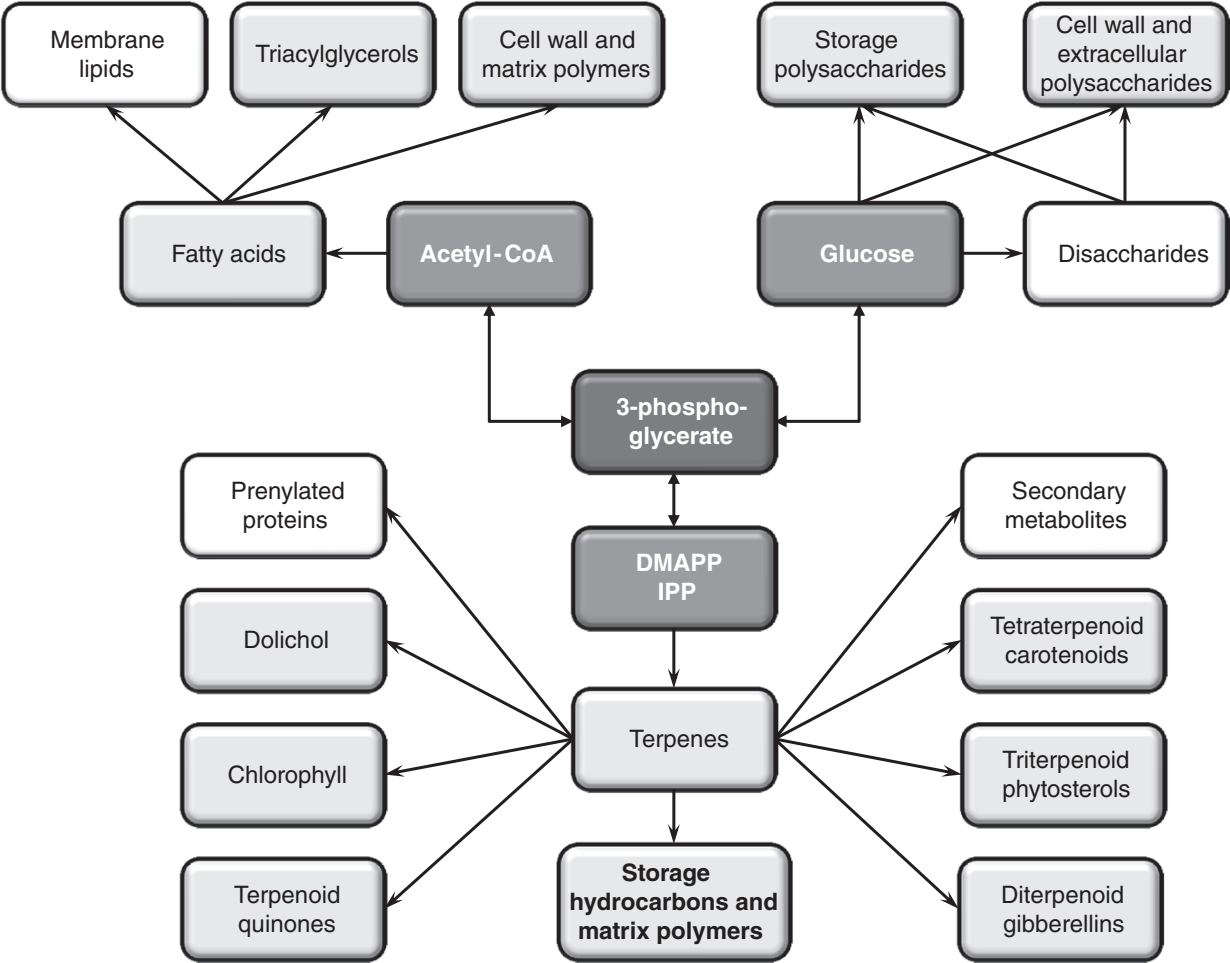
**Reconstruction of the liquid hydrocarbon biosynthesis network and selected competing pathways in*B. braunii*Showa.** Central metabolites are shown in dark boxes, metabolite groups whose biosynthetic pathways are analysed in the text are in light shaded boxes, and metabolite groups whose biosynthetic pathways are not discussed in the text are in white boxes.

Economic viability demands significant improvements in biofuel feedstock yields from microalgae [5]. This necessitates a much better understanding of cellular metabolic networks, in particular those that are directly implicated in the production of biofuel feedstocks and their metabolic inputs. While this manuscript was in preparation, a series of pioneering articles from the Watanabe lab provided the first global picture of the transcriptomes of *B. braunii* strains, based on assemblies of limited cDNA end sequencing (11,904 reads for the race B strain BOT-70) and next generation sequencing data (185,936 reads for the race A strain BOT-88-2, and 209,429 reads for the race B strain BOT-22) [29-32]. Simultaneously, the Chappell, Okada, and Devarenne laboratories published several articles on the characterization of selected key enzymes and their encoding genes for triterpene hydrocarbon biosynthesis (squalene synthase like-1, 2, and 3; triterpene methyltransferase-1, 2, and 3; and deoxyxylulose phosphate synthase-I, II, and III) from *B. braunii*, race B, strain Showa [33-35]. In the current study, we have extended these findings to provide a detailed global picture of the biosynthesis of the terpenome of the *B. braunii* Showa strain (Figure 1), using transcriptomic information in the absence of a genome sequence. Exploiting an extensive next-generation sequence read dataset generated by the Department of Energy Joint Genome Institute (JGI), we have performed a *de novo* assembly and annotation of the transcriptome of *B. braunii* Showa, and successfully identified and manually curated the genes for the key enzymes in the biosynthesis of liquid triterpenoid compounds. We describe here reconstructed pathways for the production of terpene precursors from photosynthetic 3-phosphoglycerate, the biosynthesis of terpene backbones, and the conversion of these scaffolds into liquid hydrocarbons and phytosterols. We discuss competing terpenoid pathways that channel photosynthetic carbon into tetraterpenoid carotenoids, diterpenoids, and the prenyl chains of meroterpenoid quinones and chlorophyll. We also provide an inventory of machine-annotated contigs that represent genes for the biosynthesis of competing storage compounds, including fatty acids and triacylglycerols (TAGs), and carbohydrates like starch (Figure 1). Inventories are also presented for genes whose putative protein products are predicted to be involved in *S*-adenosylmethionine regeneration, and those that may take part in the extracellular localization of the hydrocarbon oils by active transport and possibly by autophagy.

## Results and discussion

### Biomass sample collection and RNA isolation from the Showa strain of *B. braunii*

The advent of massively parallel high throughput cDNA sequencing techniques now allows the cost-effective *de novo* assembly and analysis of the transcriptomes of organisms with still unsequenced genomes [23,24]. The genome size of *B. braunii* Showa has recently been measured at 166 Mbp, significantly larger than that of the largest sequenced Chlorophyta alga, *V. carteri* at 138 Mbp [36]. This large genome, and the long and frequent repeat regions present in the *B. braunii* genome (A. Koppisch, personal communication) make the sequencing and analysis of this genome challenging. Although a transcriptome sequence database cannot capture untranscribed genomic regions (for example promoters) and does not reflect post-transcriptional regulation, it can still serve as a useful tool for gene discovery and metabolic pathway and network reconstruction, and will inform further proteomic and genomic analyses.

As a first step to construct such a database, we isolated total RNA from seven time points (days 0, 3, 5, 8, 14, 18, and 22) during the four week culture cycle of the Showa strain of *B. braunii*, race B. These time points are centered on the early portion of the culture cycle (days 0–8) because previous studies have shown that the biosynthesis and accumulation rate of botryococcenes is maximal in this period [35,37,38]. Additionally, both enzyme activity and gene expression associated with botryococcene biosynthesis have been shown to be maximal during these early time points [35,37,38]. Because of this, we hoped that the resulting transcriptomic database would be enriched for transcripts related to liquid hydrocarbon biosynthesis.

Total RNA from each time point was purified using TRIzol and LiCl precipitation to eliminate co-purifying polysaccharides. Each sample was analyzed for protein and polysaccharide contaminations ( Additional file 1: Table S1) and 5-μg aliquots from the samples with the most RNA (days 0, 3, and 5) were analyzed on a denaturing agarose gel ( Additional file 1: Figure S1A). To further confirm successful isolation of good quality RNA, RT-PCR was carried out to amplify cDNA fragments for several *B. braunii* Showa genes (Additional file 1: Figure S2), including squalene synthase (SS) [37] and squalene synthase-like-1 (SSL-1) [34]. All of the RNA samples were then pooled into a single sample, treated with DNAse (Additional file 1: Figure S1B), and analyzed for any remaining RNase contamination (Additional file 1: Figure S1C). These analyses indicated that the isolated RNA was of high quality and did not contain active nucleases. The pooled RNA sample was submitted to the Department of Energy Joint Genome Institute (JGI) for transcriptome sequencing.

### *De novo* assembly of the *B. braunii* Showa transcriptome

454 pyrosequencing yielded 1,334,609 reads (620 Mb of data) representing the ESTs from mRNA isolated from whole cells of a near-axenic *B. braunii* Showa culture [36] as described above. The reads have been deposited by the JGI into two publicly available Sequence Read Archive accessions, SRX028986 and SRX028987. We assembled these reads into 46,422 contigs with an average length of 756 bp (Additional file 1: Figure S3) using a multi-step, recursive sequence assembly protocol [39,40] as described in the Methods. This new transcriptome assembly provides significantly improved coverage over that of Watanabe *et al.* for a different B race strain of *B. braunii* (27,427 contigs with an average length of 267 bp for strain BOT-22 [32]). Contig coverage in the Showa transcriptome assembly spans three orders of magnitude, from 1X to 8,231X. The most highly represented transcripts either encode proteins related to photosynthesis, or do not show significant similarity to sequences in the GenBank non-redundant database (E-value < 1e-5, Additional file 1: Table S2). To benchmark the quality of our assembly, we compared the *B. braunii* Showa transcriptome to the core set of 458 conserved proteins (CEGs) that occur in a wide variety of eukaryotes [41]. Using the Core Eukaryotic Genes Mapping Approach (CEGMA) algorithm [41,42], we recovered 451 out of the 458 core proteins (98.4%, E value cutoff ≤ 1e-5), with 325 out of the 458 CEGs (71.0%) yielding alignments whose lengths exceed 60% of either the CEG or the contig sequence.

### Functional annotation, web-based annotation tool and data depository

Functional annotations were assigned to all unique transcript sequences using a previously described annotation pipeline [28]. The Kyoto Encyclopedia of Genes and Genomes (KEGG) [25], MetaCyc [43], Reactome [44], Panther [45], and Pfam [46] annotation databases were chosen to provide biological pathway, ontology, and protein domain annotations. Orthologous proteins from *C. reinhardtii* (a model green alga) and *Arabidopsis thaliana* (thale cress) were also used to infer ontology identifiers from their respective Gene Ontology and MapMan Ontology sets [47] (Additional file 1: Table S3). Functional annotations were assigned to 20,906 transcripts (45%), while 8,575 sequences yielded significant BlastX hits (E value <1e-5) against the non-redundant protein database of GenBank. Species in the green plant lineage were the most frequent sources from which functional annotations were derived. Top-hit analysis of KEGG protein alignments producing pathway annotations confirmed the algal character of the transcripts (Additional file 1: Table S4). Eleven of the top 15 organisms are algae or land plants, with the two top organisms, *V. carteri* and *C. reinhardtii*, together contributing 25% of all KEGG pathway assignments. An analysis of the contigs by the Metagenomics RAST server [48] found top Blast hits to proteins from fungi (Ascomycota and Basidiomycota, 19.8% of the contigs), animals (Chordata, Arthropoda, and Nematoda, 10.7% of the contigs), and bacteria (Firmicutes, Proteobacteria, and Actinobacteria, 10.1% of the contigs). However, this analysis likely provides a highly inflated estimate of non-Botryococcal transcripts in our database. While some of these transcripts may indeed originate from contaminating organisms reflecting the non-axenic nature of the algal culture and/or sample handling mistakes introduced during the sequencing process, others still likely represent genuine *B. braunii* transcripts. These transcripts may highlight gaps and biases in the GenBank database, reflect localized high similarities to phylogenetically distant homologues, originate from sequencing/assembly mistakes, or represent genes from recent horizontal gene transfer.

**Figure 2:**
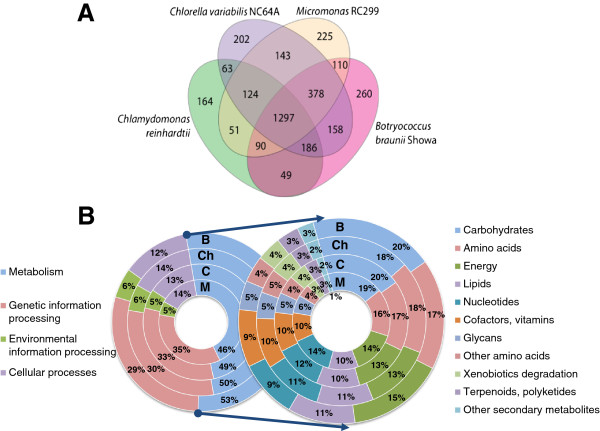
**Comparisons of KEGG annotations for Chlorophyta algae. A**. Overlap amongst KEGG annotation objects assigned to one or more genes/contigs in the transcriptome of *B. braunii* Showa, and the genomes of *C. reinhardtii*, *Ch. variabilis* NC64A, and *Micromonas* RC299. Contigs with KEGG annotations found only in the *B. braunii* Showa transcriptome are listed in Additional file 1: Table S5. B. Distribution of genes/contigs annotated with KEGG ontology categories. Circles on left: first-level ontologies; circles on right: second-level metabolic process ontologies. **B**, *B. braunii* Showa; Ch, *Ch. variabilis* NC64A; **C**, *C. reinhardtii*; M, *Micromonas* RC299.

Four-way comparisons amongst the genome sequences of selected green algae and the Showa transcriptome revealed 1,297 KEGG objects that are shared by *B. braunii*, *Micromonas sp.* RC299, *Chlorella variabilis* NC64A, and *C. reinhardtii* (Figure 2A), while 260 KEGG objects were found only in *B. braunii* Showa ( Additional file 2: Table S5). This is likely an overestimate of the true number of KEGG-annotated genes unique to *B. braunii*. First, several plastid-encoded transcripts (e.g. psbA, psbB) are present in our transcriptome assembly that are not found in the nuclear genome annotations of the other three species. Second, contigs representing transcripts of non-*Botryococcus* origin are also present in our database due to the non-axenic nature of the culture from which the mRNA was isolated (see examples in the later sections). Nevertheless, KEGG ontology terms assigned to contigs in our transcriptome show a distribution similar to those in the sequenced genomes of other Chlorophyta algae (Figure 2B).

**Figure 3:**
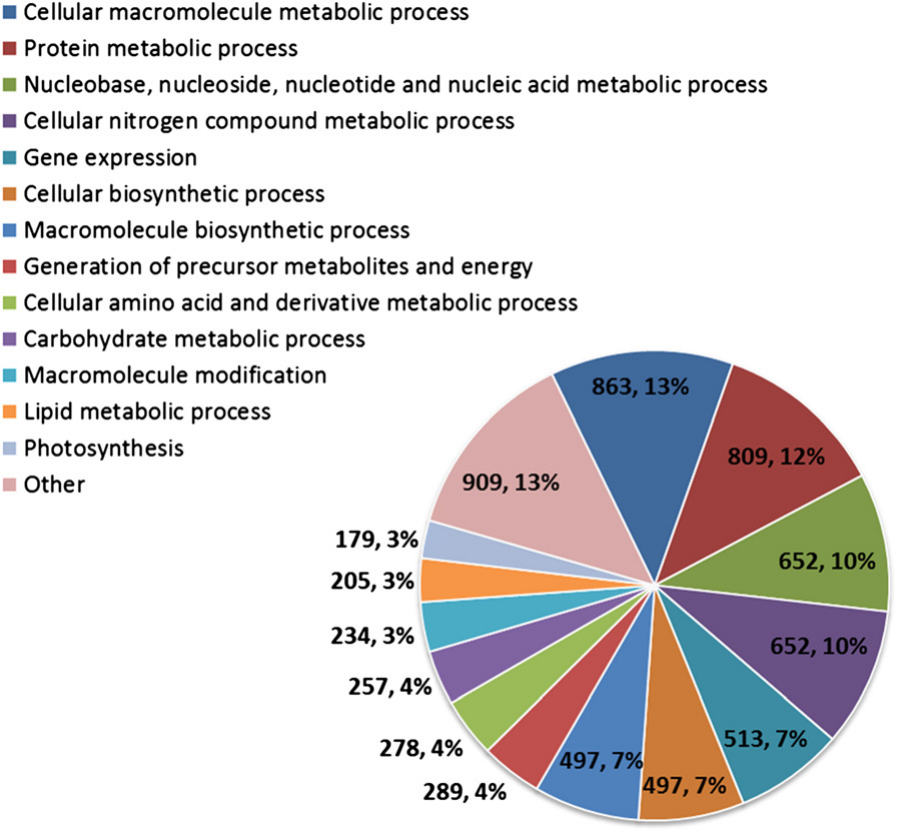
***B. braunii*Showa contigs annotated with Gene Ontology terms for molecular function.**

Inspection of the completeness of pathway assignments shows that most reactions in the majority of KEGG pathways are catalyzed by at least one predicted enzyme in the *B. braunii* Showa transcriptome. The distribution of molecular function GO (Gene Ontology) identifiers reveals the diversity of annotation categories assigned to the contigs (Figure 3). Thus, the annotation shows that we have assembled a comprehensive database of the *B. braunii* Showa transcriptome that may serve as a valuable reference for investigating the metabolic capabilities of this organism.

In order to facilitate the exploration of the data and to expedite future *B. braunii* omics and functional genetic studies, the sequences and their annotations have been deposited into the Algal Functional Annotation Tool [28] and are publicly accessible from a web-based portal at http://pathways-pellegrini.mcdb.ucla.edu/botryo1. A combined view of annotations from all primary databases for any particular transcript can be accessed by the transcript ID, and provides pathway, ontology, and protein domain data alongside primary sequence data. Functional enrichment testing and dynamic pathway visualization may be performed for lists of transcripts from within the annotation tool. Transcripts may also be looked up by biological function using a keyword search tool that returns lists of transcripts with annotations matching a keyword or a phrase. Lastly, a pathway browser tool allows visualization of transcripts for any KEGG pathway of interest. The public repository of the *B. braunii* Showa transcriptome assembly and the utilities provided to query the data will furnish a platform to update annotations as these are made available by future studies and characterization of additional strains.

### Manual curation and pathway reconstruction of the terpenome

Biosynthesis of terpenes yields a large variety of essential primary metabolites and specialized secondary metabolites in plants. Terpene biosynthesis provides membrane sterols, phytohormones, carrier molecules for N-glycan biosynthesis, pigments and antioxidants, volatile oils, aroma compounds and resins, and various toxins. Terpene biosynthesis also provides the prenyl side chains of photosynthetic pigments and meroterpenoid carrier molecules in oxidative phosphorylation, as well as polyprenyl compounds for the prenylation of proteins [49,50]. Crucially, it also underpins the production of extracellular liquid hydrocarbons and contributes to the polymeric extracellular matrix materials in race B strains of *B. braunii*[11,51]. C_30_-C_37_ triterpenes, in the form of methylated, oxidized, and cyclized botryococcenes, as well as methylated squalene, are initially synthesized inside the cells and at least botryococcenes can be found in intracellular oil bodies [11]. However, the majority (95%) of the botryococcenes are deposited into the colony extracellular matrix [10]. These oils that may be described as “bio-crude” are of prime interest for the biofuel industry as petroleum replacements.

To derive a comprehensive picture of the biosynthesis of the terpenome of *B. braunii* Showa, contigs in our databases that encode proteins for metabolic pathways yielding terpenes and their precursors (Figure 1) were collected and supplemented by further contigs identified by targeted Blast searches and Pfam keyword inquiries, as described in the Methods section. Contigs were extended and assembled into isotigs, and sequence discrepancies and potential frameshifts in the derived protein models were resolved using manual alignments and scrutiny of the assemblies. Manually curated transcript sequences (contigs and isotigs) are referred heretofore as “curated contigs”, while transcripts assembled without human supervision are simply called “contigs”. Contigs and curated contigs were considered as potentially originating from a different organism of the non-axenic culture if their deduced protein products show the highest similarities to fungal, animal or bacterial proteins with no or very low similarities to plant proteins, and if at the same time their codon usage significantly differs from that of *B. braunii* (http://www.kazusa.or.jp/codon/index.html). Subcellular localization of the deduced proteins was also predicted using TargetP (http://www.cbs.dtu.dk/services/TargetP/).

Based on databank similarities and the prediction of facile translational start and stop codons, full-size protein models were derived for approximately 30% of the manually curated transcripts. A distinguishing feature of *B. braunii* Showa transcripts is their surprisingly long (>1-2 kb) 3’ untranslated regions (UTRs), as already noted by Okada *et al.*[35]. Machine-assembled contigs with moderate to high sequence coverage, apparently derived from these long 3’ UTRs, are abundant in the transcriptome database. These may be partially responsible for the relatively large number of contigs in the dataset, and are also in apparent congruence with the large genome size of *B. braunii*[36,52].

Transcript abundances were approximated from sequence coverage, i.e. the number of primary sequence reads for a particular contig divided by the length of that contig. True estimation of expression levels would require the utilization of other techniques like qRT-PCR, DNA microarray analysis, or proteomics [18,21,22].

### Biosynthesis of terpene precursors

**Figure 4:**
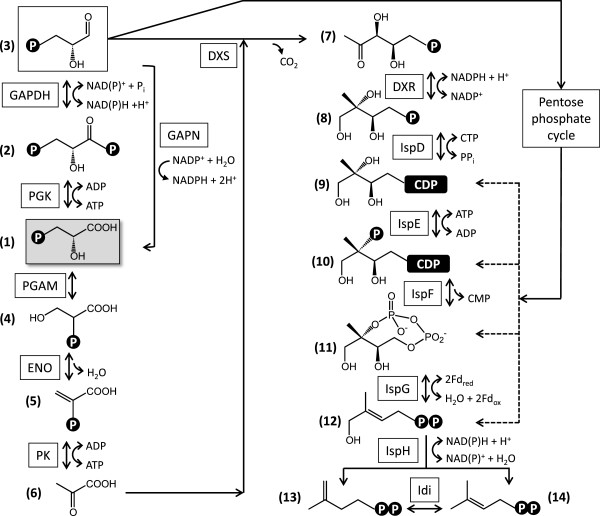
**Reconstruction of the biosynthesis of terpenoid precursors in *B. braunii*Showa.** 1, 3-phospho-D-glycerate; 2, 3-phospho-D-glyceroyl phosphate; 3, D-glyceraldehyde 3-phosphate; 4, 2-phospho-D-glycerate; 5, phosphoenolpyruvate; 6, pyruvate; 7, 1-deoxy-D-xylulose 5-phosphate; 8, 2-*C*-methyl-D-erythritol 4-phosphate; 9, 4-diphosphocytidyl-2-*C*-methyl-D-erythritol; 10, 4-diphosphocytidyl-2-*C*-methyl-D-erythritol 2-phosphate; 11, 2-*C*-methyl-D-erythritol 2,4-cyclodiphosphate; 12, (*E*)-4-hydroxy-3-methylbut-2-en-1-yl diphosphate; 13, isopentenyl diphosphate; 14, dimethylallyl diphosphate. PGK, phosphoglycerate kinase; GAPDH, glyceraldehyde phosphate dehydrogenase; GAPN, NADP^+^-dependent glyceraldehyde 3-phosphate dehydrogenase; PGAM, phosphoglycerate mutase; ENO, phosphopyruvate hydratase; PK, pyruvate kinase; DXS, 1-deoxy-D-xylulose 5-phosphate synthase; DXR, 1-deoxy-D-xylulose-5-phosphate reductoisomerase; IspD, 2-*C*-methyl-D-erythritol 4-phosphate cytidylyltransferase; IspE, 4-(cytidine 5’-diphospho)-2-*C*-methyl-D-erythritol kinase; IspF, 2-*C*-methyl-D-erythritol 2,4-cyclodiphosphate synthase; IspG, (*E*)-4-hydroxy-3-methylbut-2-enyl diphosphate synthase; IspH, 4-hydroxy-3-methylbut-2-enyl diphosphate reductase; Idi, isopentenyl-diphosphate Δ-isomerase. Fd, ferredoxin.

Terpenes are biosynthesized from the universal C_5_ building blocks isopentenyl diphosphate (IPP **13**, Figure 4) and dimethylallyl diphosphate (DMAPP, **14**). These precursors originate from the mevalonate (MVA) pathway in the cytosol of animal, fungal, archaeal, Gram-positive coccus, and higher plant cells, while the methylerythritol 4-phosphate/deoxyxylulose phosphate (MEP/DOXP) pathway is operational in plant plastids and many Gram-positive and Gram-negative Eubacteria. Genomic and biochemical evidence indicates that green algae (Chlorophyta) may have lost the MVA pathway, while other photosynthetic eukaryotes including land plants (Streptophyta) generally retain a functional contingent of the MVA pathway enzymes [49]. Experimental evidence from *B. braunii* also argues for the exclusive utilization of the MEP/DOXP pathway for terpene precursor biosynthesis in this alga [53]. Fittingly, exhaustive searches of the assembled *B. braunii* Showa transcriptome (and the unassembled singletons) identified ESTs only for the first two of the six enzymes of the MVA pathway (Additional file 1: Table S6) [54]. These two enzymes, a predicted acetyl-CoA carboxylase (AtoB, E.C. 2.3.1.9) and a deduced hydroxymethylglutaryl-CoA synthase (HMGS, E.C. 2.3.3.10), were found to be encoded by a single curated contig each with low sequence coverage (<25 reads/kb), hinting at low abundance of the corresponding mRNA species in *B. braunii* Showa. Further curated contigs in our assembly for presumed AtoB and HMGS, and for a third predicted MVA pathway enzyme, hydroxymethylglutaryl-CoA reductase (HMGR, E.C. 1.1.1.34) display very high identities to fungal sequences and show a highly divergent codon usage compared to that of *B. braunii*. These transcripts may have originated from cohabiting fungi of the non-axenic culture used for RNA isolation, or may result from very recent horizontal gene transfers into the alga. Both AtoB and HMGS are involved in various catabolic processes, including the degradation of ketone bodies and branched-chain amino acids. Thus, their deduced presence in the *B. braunii* Showa transcriptome is not an indication for the presence of a functional MVA pathway.

In contrast, a complete contingent of deduced enzymes for the MEP/DOXP pathway [49,55,56] is well represented in the *B. braunii* Showa transcriptome. The MEP/DOXP pathway uses D-glyceraldehyde 3-phosphate and pyruvate as its metabolic input. Photosynthetic 3-phospho-D-glycerate (**1**) may be converted to D-glyceraldehyde 3-phosphate (**3**) via 3-phospho-D-glyceroyl phosphate (**2**) by the collective action of phosphoglycerate kinase (PGK, E.C. 2.7.2.3) and glyceraldehyde phosphate dehydrogenase (GAPDH, Figure 4, Additional file 1: Table S6). PGK is represented in our transcriptome by three nonredundant curated contigs for three presumed isoenzymes, one of which may have originated from a fungal cohabitant. Curated contigs for one predicted isoform of the NADP^+^-dependent GAPDH (E.C. 1.2.1.13, Benson-Calvin cycle) and five inferred isoforms (three of them probably of fungal origin) of the NAD^+^-dependent GAPDH (E.C. 1.2.1.12, glycolysis and gluconeogenesis) were also identified. During glycolysis, D-glyceraldehyde 3-phosphate (**3**) may also be converted to (**1**) by the non-phosphorylating NADP^+^-dependent glyceraldehyde 3-phosphate dehydrogenase (GAPN, E.C. 1.2.1.9), encoded by a curated contig with moderate coverage (25–99 reads/kb) in the dataset (Additional file 1: Table S6). TargetP predictions for the subcellular localization of these enzymes provided support for the mitochondrial targeting of the putative NAD^+^-dependent GAPDH (E.C. 1.2.1.12) isoforms, and cytosolic localization for the PGK isozymes. Some transcripts for inferred PGK and NAD^+^-dependent GAPDH (E.C. 1.2.1.12) are present at very high abundance (>250 reads/kb) in the transcriptome, suggesting a high flux involving D-glyceraldehyde 3-phosphate in the glycolysis/gluconeogenesis pathways in *B. braunii* Showa. Contigs encoding short fragments of PGK and GAPDH orthologs have recently been described from the race B *B. braunii* strain BOT-22 [32]. These show 89-99% identity at the amino acid level with the corresponding enzymes from *B. braunii* Showa predicted in this study.

Pyruvate (**6**) is formed from (**1**) in three steps by phosphoglycerate mutase (PGAM, E.C. 5.4.2.1), phosphopyruvate hydratase (ENO, E.C. 4.2.1.11) and pyruvate kinase (PK, E.C. 2.7.1.40) during glycolysis/gluconeogenesis (Figure 4). All these enzymes are encoded in the transcriptome as multiple deduced isozymes (Additional file 1: Table S6). Curated contigs 10955, 16949, 23205, and 41366 show the highest similarities to individual domains of PKs with four similar catalytic domains each, thus probably representing a single multifunctional enzyme. All these curated contigs have moderate coverage. Curated contig 43373, encoding a predicted cytoplasmic ENO, is the only exception and is represented by very abundant ESTs. Two contigs from *B. braunii* BOT-22 (FX085139 and FX085140) encoding short regions of PK [32] show 94% amino acid identity to two inferred PK isozymes encoded in our dataset (curated contig 10955 and 41736, respectively).

Four curated contigs code for three predicted isozymes of the first enzyme of the MEP/DOXP pathway, the thiamine diphosphate-dependent 1-deoxy-D-xylulose 5-phosphate synthase (DXS, E.C. 2.2.1.7) that produces 1-deoxy-D-xylulose 5-phosphate (**7**) from (**3**) and (**6**) (Figure 4, Additional file 1: Table S6). Multiple isoenzymes of DXS are routinely found in land plants [57], and clade into three phylogenetically distinct families [58]. Constitutively expressed DXS isozymes of these plants produce precursors for essential terpenoids, while certain inducible DXS isozymes specialize in stress response and ecological interactions with symbionts or pathogens [57]. In contrast, genomic evidence shows that strains of green algae harbour only a single DXS each. These proteins form a sister clade to the three DXS clades of land plants [35,49]. The Okada group has recently reported the cloning and biochemical characterization of three isozymes of DXS from *B. braunii* Showa [35]. Interestingly, these three isoenzymes all fall into the basal clade for Chlorophyta DXSs, thus representing paralogous sequences resulting from gene duplications within the green algal lineage [35]. All three isozymes were found to be expressed simultaneously, and show similar kinetic parameters except for a higher temperature tolerance for DXS-III [35]. Our data also indicate similar, moderate transcript abundances for DXS-I (curated contig 10163, 66.6 reads/kb) and DXS-II (curated contig 42027, 93.7 reads/kb), with a somewhat lower abundance for DXS-III-related ESTs (curated contigs 07667 and 11032, 4.0 reads/kb and 29.4 reads/kb, respectively). Short curated contigs representing orthologs of DXS-II (FX085276 and FX085277) and DXS-III (FX085274 and FX085275) were also identified in the preliminary transcriptomic analysis of the *B. braunii* race B strain BOT-22 [32], with amino acid identities to the Showa enzymes in the 72-84% range ( Additional file 1: Table S6). All three DXS isozymes of the Showa strain have been found to contain chloroplast targeting sequences at their N-termini [35], in agreement with our TargetP predictions ( Additional file 1: Table S6). DXS has been described as one of the rate-limiting steps of the MEP/DOXP pathway in plants [49,57], thus the expression of three isoforms of this enzyme in *B. braunii* Showa might provide an increased metabolic flux for the production of terpenoid precursors. Alternatively, each DXS isoform might be associated with the production of a specific class of terpenoids. Considering the absence of the cytoplasmic mevalonate pathway as an alternative to furnish isoprene precursors, evolutionary optimization of the DXS step by repeated gene duplications providing parallel capacity for the production of (**7**) might have been beneficial for *B. braunii* race B strains.

1-deoxy-D-xylulose 5-phosphate (**7**) is converted to 2-*C*-methyl-D-erythritol 4-phosphate (**8**, Figure 4) by the NADPH-dependent enzyme 1-deoxy-D-xylulose-5-phosphate reductoisomerase (DXR, E.C. 1.1.1.267). A single deduced isozyme of DXR is encoded in our dataset by a curated contig with moderate coverage (Additional file 1: Table S6). The DXR-catalyzed reaction is the first committed step towards the production of isoprene precursors since (**7**) is also utilized for the biosynthesis of thiamine diphosphate and pyridoxal phosphate [57]. The rest of the pathway involves the CTP-dependent conversion of (**8**) to 4-diphosphocytidyl-2-*C*-methyl-D-erythritol (**9**) by IspD (2-*C*-methyl-D-erythritol 4-phosphate cytidylyltransferase, E.C. 2.7.7.60), and the ATP-dependent phosphorylation of (**9**) to 4-diphosphocytidyl-2-*C*-methyl-D-erythritol 2-phosphate (**10**) by IspE (4-(cytidine 5’-diphospho)-2-*C*-methyl-D-erythritol kinase, E.C. 2.7.1.148). Curated contigs for these two inferred enzymes are present in the Showa transcriptome at moderate coverage. Formation of 2-*C*-methyl-D-erythritol 2,4-cyclodiphosphate (**11**) from (**10**) by IspF (2-*C*-methyl-D-erythritol 2,4-cyclodiphosphate synthase, E.C. 4.6.1.12) releases CMP, followed by the two-electron reduction of (**11**) to (*E*)-4-hydroxy-3-methylbut-2-en-1-yl diphosphate (**12**) by the [4Fe-4S] enzyme IspG (4-hydroxy-3-methylbut-2-enyl diphosphate synthase, E.C. 1.17.7.1). The last step of the MEP/DOXP pathway is the formation of both isopentenyl diphosphate (IPP, **13**) and dimethylallyl diphosphate (DMAPP, **14**) from (**12**) by another [4Fe-4S] enzyme, IspH (also known as LytB, 4-hydroxy-3-methylbut-2-enyl diphosphate reductase, E.C. 1.17.1.2)[59]. Both IspG and IspH accept electrons from a ferredoxin. These electrons may originate directly from the photo-oxidation of water during photosynthetic conditions in the chloroplast without the involvement of reducing cofactors, while a ferredoxin reductase is required in the dark to channel electrons from cellular pools of NADPH [57]. The branching reaction catalysed by IspH is in stark contrast to the MVA pathway that yields IPP (**13**) exclusively [59], which has to be later isomerised to DMAPP (**14**) by Idi (isopentenyl-diphosphate delta-isomerase, E.C. 5.3.3.2). Each of the predicted enzymes for the downstream half of the MEP/DOXP pathway (IspF and onwards) are encoded by single nonredundant curated contigs with high to very high sequence coverage in the *B. braunii* Showa transcriptome (200.7, 242.9, and 426.6 for IspF, IspG and IspH, respectively), indicating vigorous transcription and perhaps robust metabolic flow through these enzymes. This is in contrast to some plant systems where the same enzymes were found to be rate-limiting [49,57]. Two presumed isozymes of Idi are encoded by two nonredundant curated contigs with only low coverage in the Showa transcriptome. Another curated contig with moderate coverage for a putative fungal IPP isomerase was also identified (34876, Additional file 1: Table S6). Although Idi is generally present in organisms utilizing the MEP/DOXP pathway for terpenoid precursor biosynthesis, it has not been found strictly essential and plays only a supplementary role in optimizing IPP and DMAPP ratios [57]. Type II IPP isomerases, detected in *Streptomyces* spp. and in *Synechocystis* spp. [60,61], were not found in the Showa transcriptome. TargetP predictions for all MEP/DOXP enzymes suggest chloroplast targeting, with the exception of IspH where both chloroplast and mitochondrial targeting seems equally plausible (Additional file 1: Table S6).

The cyanobacterium *Synechocystis sp.* PCC6803 contains no MVA pathway but features a full complement of the MEP/DOXP pathway. Nevertheless, neither pyruvate (**6**) nor 1-deoxy-D-xylulose 5-phosphate (**7**) has been observed to stimulate IPP biosynthesis in cell extracts. Similarly, fosmidomycin has not been seen to inhibit DXR by reducing IPP biosynthesis *in vitro* or by reducing cellular growth *in vivo*[62]. On the other hand, metabolites of the reductive pentose phosphate cycle, especially D-xylulose 5-phosphate, increased IPP formation even in the presence of the DXR inhibitor fosmidomycin [62]. While the immediate entry point of the pentose phosphate cycle metabolites into the MEP/DOXP pathway is currently not known, it is assumed to be downstream of DXR (Figure 4). A survey of the Showa transcriptome identified curated contigs encoding putative chloroplast-targeted RPE (ribulose phosphate 3-epimerase, E.C. 5.1.3.1) and thiamine diphosphate-dependent TKTL (transketolase, E.C. 2.2.1.1) enzymes that yield D-xylulose 5-phosphate in the pentose phosphate cycle (Additional file 1: Table S6). Short contigs that encode fragments of the TKTL enzyme have recently been identified from *B. braunii* BOT-22 (FX085315 and FX085314) [32], and show 87-90% amino acid identities with the enzyme encoded by curated contig 32329 of the Showa transcriptome. Transcripts of putative fungal origin were also detected for both RPE and TKTL. Thiamine diphosphate-dependent phosphoketolase (XFP, E.C. 4.1.2.9) was represented only by a single curated contig of potential fungal origin. This enzyme generates (**3**) and acetyl phosphate from D-xylulose 5-phosphate and inorganic phosphate. Curated contig 30447 for RPE has moderate sequence coverage at 55.8 reads/kb, while reads for curated contig 32329 for TKTL are highly abundant at 436.6 reads/kb.

While the importance, or even the dominance, of the pentose phosphate cycle for isoprene precursor biosynthesis has been speculated upon for the BOT-22 and BOT-70 strains of *B. braunii* race B [31,32], these recent studies employed limited transcriptomic and EST datasets. Our data support the presence of a fully functional MEP/DOXP pathway in *B. braunii* Showa, with multiple paralogous DXSs with moderate EST coverage for each, providing a reasonable entry for isoprene precursor biosynthesis. The sequence coverage of the inferred downstream half of the MEP/DOXP pathway enzymes (IspF and onwards) is higher than that of the upstream half of the pathway, which would be consistent with an anaplerotic feed of metabolites from the pentose phosphate cycle. Future studies of the dynamics of transcription of the MEP/DOXP pathway and the pentose phosphate cycle enzymes by e.g. qRT-PCR, in relation to the age of the culture and hydrocarbon production levels would be necessary to shed more light on this issue. Investigating the inhibition by fosmidomycin of *B. braunii* cultures *in vivo* and/or the isolated Showa DXR enzyme *in vitro* may also provide further clues to map the flux of metabolites through these alternative biosynthetic routes.

### Terpene backbone biosynthesis

**Figure 5:**
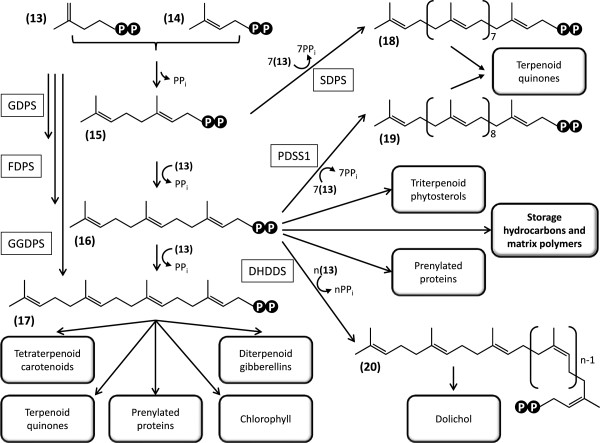
**Reconstruction of the biosynthesis of linear terpene backbones in *B. braunii*Showa.** 15, Geranyl diphosphate; 16, 2-*trans*, 6-*trans*-farnesyl diphosphate; 17, *all-trans*-geranylgeranyl diphosphate; 18, *all-trans*-nonaprenyl diphosphate; 19, *all-trans*-decaprenyl diphosphate; 20, *di-trans*, *poly-cis*-polyprenyl diphosphate (n=10-55). GDPS, geranyl diphosphate synthase; FDPS, farnesyl diphosphate synthase; GGDPS, geranlylgeranyl diphosphate synthase; SDPS, solanesyl diphosphate synthase; PDSS1, decaprenyl diphosphate synthase; DHDDS, dehydrodolichyl diphosphate synthase.

Terpenoids are derived from linear polyprenyl diphosphate chains that are generated by the stepwise recursive head-to-tail (1’-4) condensation of IPP (**13**) first with DMAPP (**14**) and further with allylic polyprenyl diphosphates with the concomitant release of pyrophosphate in every condensation cycle (Figure 5). These alkylations are catalysed by a family of polyprenyl diphosphate synthases (prenyl transferases) that are present in widely varying numbers in plants [63]. Prenyl transferases may have relatively relaxed substrate and product specificities, accepting DMAPP or longer allylic prenyl diphosphates and producing polyprenyls of different chain lengths [63-65].

C_10_ geranyl diphosphate (**15**, Figure 5) is synthesized by geranyl diphosphate synthase (GDPS, E.C. 2.5.1.1) from (**13**) and (**14**). **15** yields monoterpenes in plants, and serves as a precursor for solanesyl diphosphate synthase (SDPS, E.C. 2.5.1.84) that affords *all-trans*-nonaprenyl diphosphate (**18**) for the polyprenyl chains of terpenoid quinones in the mitochondria (ubiquinones) and in the chloroplast (plastoquinones). Both GDPS and SDPS are predicted to be present in the Showa transcriptome as a single nonredundant curated contig each with low sequence coverage (Additional file 1: Table S7).

Farnesyl diphosphate synthase (FDPS, E.C. 2.5.1.10) generates C_15_ farnesyl diphosphate (**16**) in two reaction steps *via***15**. **16** is the precursor for the sesquiterpenes and the triterpenoids (including squalene and phytosterols), and provides the farnesyl side chains for post-translational modification of proteins. **16** is also the substrate for decaprenyl diphosphate synthase (PDSS1, E.C. 2.5.1.91). This enzyme produces *all-trans*-decaprenyl diphosphate (**19**) for the side chain of the mitochondrial electron carrier ubiquinone-10 (coenzyme Q_10_). The *cis-*prenyltransferase dehydrodolichyl diphosphate synthase (DHDDS, E.C. 2.5.1.-) also uses **16** as its substrate to generate dehydrodolichyl diphosphates (*di-trans*, *poly-cis*-polyprenyl diphosphate, **20**) that serve as precursors to the glycosyl carrier lipid dolichol for *N*-glycan biosynthesis. Crucially, **16** is also the precursor for the liquid hydrocarbon triterpenoid botryococcenes and methylated squalenes, and the cell wall ether lipids and some of the matrix polymers of *B. braunii* Showa [9,11]. Two nonredundant curated contigs for two putative isozymes of FDPS with 72% amino acid identity were identified in the Showa transcriptome, both with moderate sequence coverage (Additional file 1: Table S7). The FDPS isozyme encoded by curated contig 15137 is predicted to be localized outside the chloroplast, the mitochondrion, or the secretory apparatus of the cell. A third curated contig for a presumed FDPS with extremely low sequence coverage may stem from a fungal source (Additional file 1: Table S7). Deduced DHDDS isozymes are encoded by four curated contigs of low sequence coverage, one of which is potentially of fungal origin. A single curated contig with low sequence coverage encodes a putative PDSS1 that may also have been derived from a fungal cohabitant (Additional file 1: Table S7).

Geranylgeranyl diphosphate (**17**) is produced by geranylgeranyl diphosphate synthase (GGDPS, E.C. 2.5.1.29), encoded in the Showa transcriptome by only a single nonredundant curated contig with moderate sequence coverage (Additional file 1: Table S7). In higher plants, this enzyme may be present as three isoenzymes - one cytosolic, one plastidic and one mitochondrial [49]. TargetP prediction supports mitochondrial localization for the single GGDPS identified in the Showa transcriptome. The expected plastidic GGDPS might be encoded by the chloroplast genome and thus likely missed by our transcriptome database. Alternatively, the other isoforms may arise from multiple targeting, or simply represent gaps in the current transcriptome database due to lower levels of expression or sequencing/assembly artifacts. Plant GGDPS enzymes may initiate synthesis of **17** from **14** (Figure 5), but the most effective substrate of GGDPSs from animals and fungi is **16**[49,65]. **17** serves as the precursor for the C_20_ diterpenes including gibberellins, the phytyl side chains of chlorophyll, phylloquinone and tocopherol (vitamin E), and the geranylgeranyl chains of prenylated proteins. **17** is also the precursor for tetraterpenoid carotenoids, including lycopene, lutein, canthaxanthin and others isolated from race L and race B *B. braunii* strains [11,66].

### Liquid triterpenoid hydrocarbon biosynthesis

**Figure 6:**
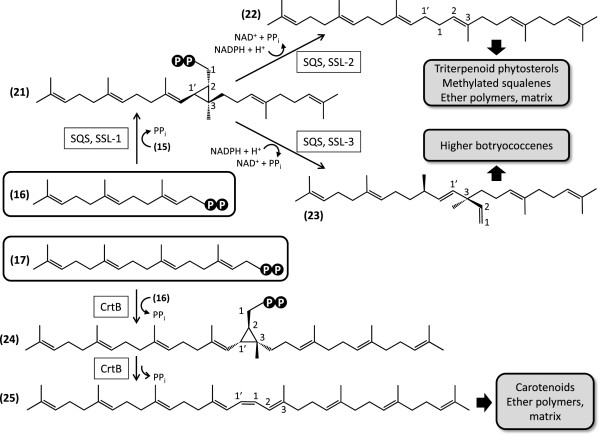
**Reconstruction of the biosynthesis of linear tri- and tetraterpenoids in *B. braunii*Showa.** 21, presqualene diphosphate; 22, squalene; 23, botryococcene; 24, (1*R*, 2*R*, 3*R*)-prephytoene diphosphate; 25, 15*-cis*-phytoene. SQS, squalene synthase; SSL-1, -2, -3, squalene synthase-like enzymes; CrtB, phytoene synthase.

Farnesyl diphosphate (**16**) serves as the precursor for the biosynthesis of the C_30_ triterpenoid structural isomers squalene and botryococcene, catalyzed by squalene synthase (SQS, E.C. 2.5.1.21) and squalene synthase-like enzyemes, respectively, in a two-step reaction. First, head-to-head condensation of two molecules of **16** yields presqualene diphosphate (**21**, Figure 6) with a C1’-2-3 cyclopropyl moiety. Next, **21** undergoes a reductive rearrangement in the presence of NADPH to afford either squalene (**22**) with a C1’-1 linkage, or botryococcene (**23**) with a C1’-3 bond connecting the two farnesyl moieties [67]. The *B. braunii* Showa SQS for the biosynthesis of squalene (termed BSS) has been cloned and expressed in *E. coli*[37]. Recombinant BSS catalysed both half-reactions in the presence of NADPH and afforded **22**, but not **23**. BSS is encoded in the Showa transcriptome by a curated contig with high sequence coverage (Additional file 1: Table S8). While botryococcene synthase activity has been demonstrated in *B. braunii* Showa cell extracts [38], the corresponding enzyme(s) and gene(s) remained elusive until the recent identification of three squalene synthase-like enzymes (SSL-1 to SSL-3) from this strain [34]. Unlike any other known natural SQS, SSL-1 catalyses only the first half reaction to produce **21**. Reactions combining SSL-1, SSL-2 and NADPH afforded squalene **22**, while those with SSL-1, SSL-3 and NADPH provided botryococcene **23** as the main product with minor amounts of **22**[34]. Thus, the solution for the problem of the biosynthesis of both squalene and botryococcene in *B. braunii* Showa involved the evolution of a novel enzyme system in addition to a conserved **22**-producing SQS by repeated gene duplications and neofunctionalization of the paralogs. In this ancillary system, the two half reactions of the canonical SQS reaction are dissociated into separate enzymes that presumably form subunits for a catalytic complex. Production of **22** by two separate enzyme systems (BSS and the SSL-1 + SSL-2 complex) might provide separate pools of this compound for primary triterpenoids (membrane sterols) and secondary triterpenoids (liquid hydrocarbons and matrix polymers) [34]. All three SSL enzymes are represented by curated contigs of moderate sequence coverage in the Showa transcriptome (Additional file 1: Table S8), with no additional SQS isozymes detectable. While BSS, SSL-1 and SSL-3 do not appear to be targeted to the chloroplast, mitochondrion or the lumen of the endoplasmic reticulum based on predictions by TargetP, botryococcene synthase activity has been shown to be associated with the membrane fraction [38]. Additionally, BSS (but not SSL-1 or SSL-3) has been described to associate with the ER using a C-terminal membrane anchoring sequence [34].

In addition to SQSs and phytoene synthases (CrtB, see next section), genome sequence surveys of algae often identify a gene encoding a putative, uncharacterized protein with the head-to-head trans-isoprenyl diphosphate synthase fold. These predicted proteins (Class 1 isoprenoid biosynthesis-related proteins [ISR]), form a clade that is distinct from SQSs and CrtB enzymes. ISRs had been hypothesized earlier to constitute the then-unknown botryococcene synthase [68], but the recent *in vitro* studies from the Chappell laboratory, as described above, conclusively showed that these proteins are not necessary for botryococcene biosynthesis [34]. A single nonredundant curated contig with low sequence coverage represents this cryptic ISR enzyme in the Showa transcriptome ( Additional file 1: Table S8).

**Figure 7:**
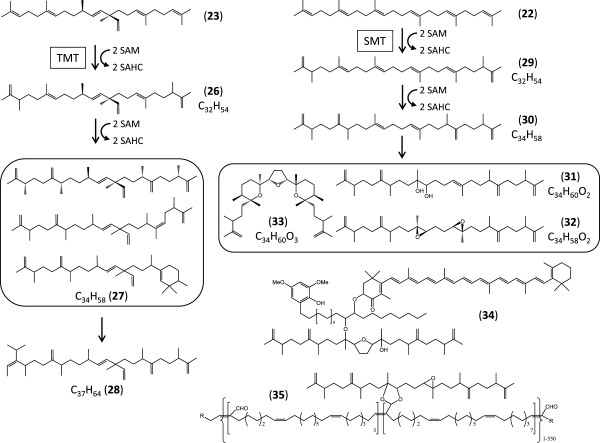
**Biosynthesis of liquid triterpenoid compounds and matrix polymer materials in *B. braunii*Showa.** 26, C_32_ botryococcene; 27, representative C_34_ isomeric botryococcenes; 28, a C_37_ botryococcene; 29, C_32_ dimethylsqualene; 30, C_34_ tetramethylsqualene; 31, dihydroxy-tetramethylsqualene; 32, diepoxy-tetramethylsqualene; 33, botryolin; 34, braunixanthin 1; 35, complex polymeric matrix lipid from *B. braunii* race **B**. TMT, terpenoid methyltransferase, SMT, squalene methyltransferase.

The extracellular liquid hydrocarbons of *B. braunii*, race B are dominated by variously methylated botryococcenes, with C_32_ and C_34_ botryococcenes as the most abundant liquid hydrocarbon oil (Figure 7) [69-71]. Methylated squalene (C_34_ tetramethylsqualene) represents approximately 4.5% of the liquid hydrocarbons in race B [72], with a higher level of tetramethylsqualene (10%) found covalently linked to polyacetals in the polymers of the colony extracellular matrix [51]. These methylated botryococcenes (C_31_ – C_37_) and methylsqualenes (C_31_ – C_34_), are biosynthesized by S-adenosylmethione (SAM)-dependent methyltransferases acting upon botryococcene **23** and squalene **22**, respectively. The biosynthesis of C_32_ botryococcene (**26**) and C_32_ methylsqualene (**29**) has recently been clarified by the Chappell group [33] while this manuscript was in preparation. This paper described the cloning, heterologous expression and functional characterization of six enzymes showing similarity to sterol 24-*C*-methyltransferases (SMT, E.C. 2.1.1.41) that catalyse single methyl additions onto the linear prenyl side chains of sterols using SAM as the methyl donor [73,74]. Three of these putative SMTs that harboured variant sterol binding domains were found to conduct two successive methyl transfers onto linear triterpenoids. Triterpenoid methyltransferase-1 (TMT-1) and TMT-2 both yielded terminal mono- and dimethylated squalene (e.g. **29**), while TMT-3 afforded terminal mono- and dimethylated botryococcene (e.g. **26**). TMT-1 and TMT-2 displayed very little activity towards C_30_ botryococcene **23**, while squalene **22** was a similarly weak substrate for TMT-3. The remaining three SMT-like enzymes (SMT-1 to SMT-3) did not accept **22** or **23** as their substrates, nor did they methylate common plant sterols in spite of harbouring apparently canonical sterol binding domains. None of the six identified enzymes, nor any of their pairwise combinations accepted the C_32_ linear triterpenoids **26** or **29**, thus the biosynthesis of the C_34_ linear triterpenoids **27** and **30**, or that of the higher botryococcene homologs like **28** remains to be elucidated. All six SMTs identified by Niehaus et al. [33] were well represented in the Showa transcriptome analysed here (Additional file 1: Table S8). Sequence coverage ranged from 71.7 reads/kb for TMT-2 to 1196.7/kb for SMT-1, with the botryococcene methyltransferase TMT-3 also exhibiting an extremely high sequence coverage at 768.4 reads/kb. Thus, the genes for these SMTs are apparently very actively transcribed, perhaps reflecting the fact that >70% of the liquid hydrocarbon oils of *B. braunii* Showa are composed of C_32_ – C_34_ botryococcenes [33]. All three TMTs are predicted by TargetP here to localize into the secretory system (endoplasmic reticulum, Golgi apparatus), and were found by Niehaus *et al*. to associate with yeast microsomes (originating from the ER) upon heterologous expression in that host [33]. Indeed, C_33_ – C_34_ botryococcenes are overwhelmingly localized in the extracellular matrix, while **23** and lesser methylated botryococcenes predominate in intracellular oil bodies [13,75]. In contrast, SMT-1, SMT-2 and SMT-3 are predicted here to be targeted to a compartment outside the chloroplast, mitochondrion or the secretory system (Additional file 1: Table S8). Our analysis has also uncovered four additional nonredundant curated contigs encoding deduced SMTs, one of them (32241) of putative fungal origin, all with low (5.7 - 15.0 reads/kb) sequence coverage. These predicted enzymes might be candidates for the missing SMTs for the biosynthesis of **27** and **30**. The biosynthetic basis for the double bond isomerization and terminal cyclization reactions that lead to the various C_34_ botryococcene isomers in the B race of *B. braunii* (**27**, Figure 7) remain unknown [9,11]. A curated contig with low sequence coverage (31093, 11.2 reads/kb) encodes an enzyme with similarity to 24-methylenesterol *C*-methyltransferases (MSMT, E.C. 2.1.1.143). These enzymes afford C24-ethyl sterols by methylating C24-methylsterols like 24-methylenelophenol [76]. It remains to be determined whether the corresponding enzyme takes part in phytosterol biosynthesis or in the production of higher botryococcenes like the C_37_ botryococcene **28** (Figure 7).

Hydroxylation, epoxidation, and formation of O-containing heterocycles (for example compounds **31**, **32**, and **33**, Figure 7) increase the complexity of race B liquid and polymer hydrocarbons. These oxidized methylsqualenes, together with carotenoids and very long chain fatty acids (and to a smaller degree oxidized botryococcenes) also support the biosynthesis of ether lipids like braunixanthin 1 (**34**) and matrix polymers (**35**) as precursors [9-11,51,77]. Candidates for the introduction of the oxygen functionality into linear triterpene structures might be enzymes similar to the flavoprotein squalene monooxygenase (SQLE, E.C. 1.14.13.132) that produces (3*S*)-2,3-epoxy-2,3-dihydrosqualene from **22** by incorporation of molecular oxygen. Enzymes similar to SQLE might also accept tetramethylsqualene **30** as their substrate and catalyse epoxide formation towards the centre of the long terpene chain, yielding compounds like diepoxy-tetramethylsqualene (**32**). Reducing equivalents are channelled to these enzymes by NADPH-dependent hemoprotein reductases. The Showa transcriptome contains seven nonredundant curated contigs with very low to low sequence coverage (3.4 to 20.2 reads/kb, Additional file 1: Table S8) encoding SQLE-like enzymes: these putative enzymes are candidates to channel squalenes and maybe botryococcenes towards the production of extracellular matrix materials.

### Biosynthesis of other terpenoids

The terpenome of *B. braunii* Showa includes meroterpenoid quinones, the side chain of chlorophyll, diterpenoid gibberellins, triterpenoid phytosterols, tetraterpenoid carotenoids, polyprenyl carrier molecules (dolichol, see above), and the prenyl chains of proteins. All these primary and secondary metabolites draw on the common isoprene (IPP and DMAPP) pool generated by the chloroplast-based MEP/DOXP pathway, and divert these precursors away from the production of liquid and matrix hydrocarbons. We have curated and catalogued contigs representing putative enzymes involved in these competing pathways.

**Figure 8:**
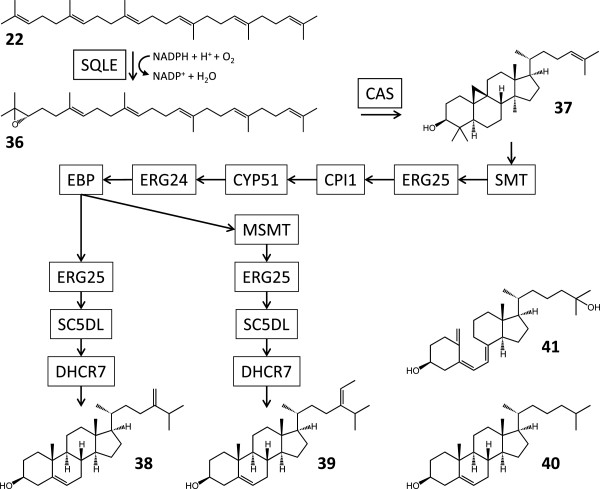
**Reconstruction of the biosynthesis of triterpenoid sterols and vitamin D**_**3**_** in *B. braunii*Showa.** 36, (*S*)-2,3-epoxisqualene; 37, cycloartenol; 38, 24-methylenecholesterol; 39, isofucosterol; 40, cholesterol; 41, 25-hydroxyvitamin D_3_ (calcidiol). SQLE, squalene monooxygenase; CAS, cycloartenol synthase; SMT, sterol 24-*C*-methyltransferase; ERG25, methylsterol monooxygenase; CPI1, cycloeucalenol cycloisomerase; CYP51, sterol 14-demethylase; ERG24, Δ-14-sterol reductase; EBP, cholestenol Δ-isomerase; MSMT, 24-methylenesterol *C*-methyltransferase; SC5DL, lathosterol oxidase; DHCR7, 7-dehydrocholesterol reductase.

Squalene **22** is the precursor for the triterpenoid phytosterols, chloroplast membrane cholesterol and its esters [73,78], and vitamin D_3_. Following oxidation of **22** to (*S*)-squalene 2,3-epoxide (**36**, Figure 8) by SQLE (see previous section), 2,3-oxidosqualene cyclases catalyse a cationic cyclization cascade converting linear triterpenes to fused ring compounds. A single curated contig with moderate sequence coverage (Additional file 1: Table S9) encodes a putative cycloartenol synthase (CAS, E.C. 5.4.99.8) which may produce cycloartenol ((3*S*)-2,3-epoxy-2,3-dihydrosqualene, **37**), an intermediate in the biosynthesis of phytosterols. The Showa transcriptome contains curated contigs for a full contingent of enzymes (Additional file 1: Table S9) to afford 24-methylenecholesterol (**38**) and isofucosterol (**39**), the main sterols in *B. braunii* strains [79]. Stigmasterol and β-sitosterol, close structural analogues of **38** and **39**, have also been identified in this alga by pyrolysis-GC/MS [80], while conventional GC/MS has identified campesterol and β-sitosterol [79]. In *C. reinhardtii*, precursors of the predominant membrane sterol ergosterol were shown to be synthesized by an identical pathway [81]. Similar enzymes, with the addition of sterol-4α-carboxylate 3-dehydrogenase (ERG26, E.C. 1.1.1.170) and vitamin D 25-hydroxylase (CYP2R1, E.C. 1.14.13.15), may also be involved in the biosynthesis of cholesterol (**40**) [79,82] and calcidiol (25-hydroxyvitamin D3, **41**) (Additional file 1: Table S9). A curated contig for a putative sterol esterase (LIPA, E.C. 3.1.1.13) may take part in the esterification of sterols with long-chain fatty acids – this enzyme may alternatively be involved in the production of ester polymers or matrix materials (**34** and **35**, see previous section).

**Figure 9:**
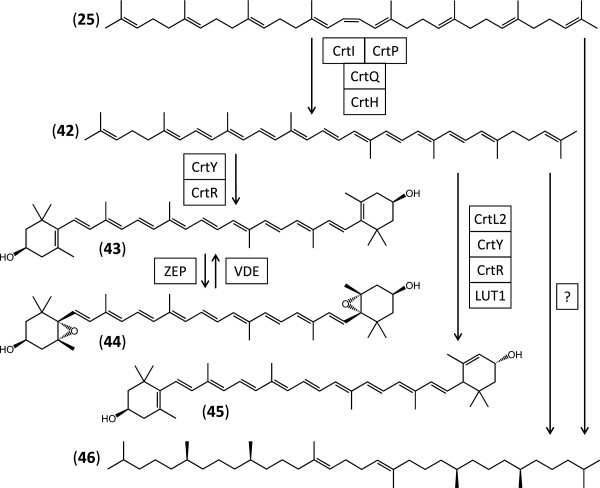
**Reconstruction of tetraterpenoid biosynthesis in *B. braunii*Showa. 42**, lycopene; **43**, zeaxanthin; **44**, violaxanthin; **45**, lutein; **46**, *trans,trans*-lycopadiene. CrtI, phytoene desaturase; CrtP, 15-*cis-*phytoene dehydrogenase; CrtQ, ζ-carotene desaturase; CrtH, prolycopene isomerase; CrtY, lycopene β-cyclase; CrtR, β-carotene 3-hydroxylase; ZEP, zeaxanthin epoxidase; VDE, violaxanthin de-epoxidase; CrtL2, lycopene ε-cyclase; LUT1, carotene ε-monooxygenase, ?, unidentified enzyme(s).

Geranylgeranyl diphosphate **17** serves as the precursor for the biosynthesis of the tetraterpenoid carotenoids that are important photoprotectants, antioxidants and membrane protein function modulators for the photosynthetic complexes [83-85]. Tetraterpenoid biosynthesis initiates with a trans-isoprenyl diphosphate synthase, phytoene synthase (CrtB, E.C. 2.5.1.32), catalysing the head-to-head condensation of two molecules of **17** in a two-step reaction with the concomitant release of pyrophosphate, paralleling the reactions of squalene synthase in triterpenoid biosynthesis discussed earlier. In the first step, (1*R*, 2*R*, 3*R*)-prephytoene diphosphate (**24**) with a C1’-2-3 cyclopropyl moiety is produced (Figure 6). Next, **24** undergoes a rearrangement, this time with no reduction, to afford 15-*cis-*phytoene (**25**) with a C1’-1 linkage. CrtB is encoded in the Showa transcriptome by a curated contig with low sequence coverage (Additional file 1: Table S10). **25** may be converted to a variety of carotenoids in *B. braunii* Showa by a complex network of enzymes, with curated contigs encoding enzymes presumed to be involved in the production of lycopene (**42**), zeaxanthin (**43**), violaxanthin (**44**), and lutein (**45**) (Figure 9, Additional file 1: Table S10). The majority of the deduced carotenoid tailoring enzymes listed in Additional file 1: Table S10 are represented by multiple nonredundant isozymes encoded in the Showa transcriptome. Some of these deduced enzymes are predicted by TargetP to be localized outside of the plastid, the mitochondrion or the secretory systems. All these contigs have low sequence coverage (less than 25 reads/kb), with the sole exception 09778, which codes for a β-carotene 3-hydroxylase (CrtR, E.C. 1.14.13.129) that has moderate sequence coverage (94.1 reads/kb). Considering the variety of predicted isozymes, and the fact that many of the carotenoid biosynthetic enzymes have somewhat broad substrate specificities and may catalyse multiple and overlapping reaction steps [85], this alga may biosynthesize a large variety of these pigments. Indeed, **4345**, as well as various other β-carotenes, echinenone, canthaxanthin, loroxanthin, and neoxanthin have been described from race B strains [66,77,86-89]. Some of these carotenoids may also be incorporated into the ether polymers and insoluble extracellular matrix materials, thus may have a structural role [9-11,51].

The prominent liquid hydrocarbon *trans,trans-*lycopadiene (**46**, Figure 9) of race L strains of *B. braunii* is probably biosynthesized by reduction of an acyclic carotenoid such as 15-*cis-*phytoene (**25**) or lycopene (**42**) [10]. Lycopadiene (**46**) is considered a biomarker for race L strains of *B. braunii* as there are no reports for the occurrence of this compound in race B strains [90]. Indeed, we could not identify plausible tetraterpene reductases in the Showa transcriptome for the biosynthesis of **46**.

**Figure 10:**
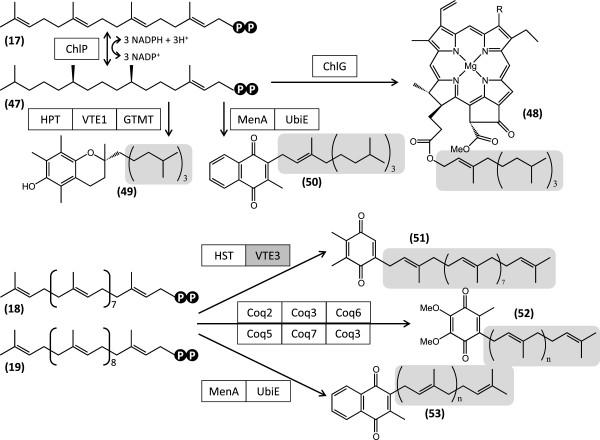
**Reconstruction of meroterpenoid quinone biosynthesis in *B. braunii*Showa.** 47, phytyl diphosphate; 48, chlorophyll a (R=CH_3_) and b (R=CH_2_O); 49, α-tocopherol (vitamin E); 50, phylloquinone (vitamin K1); 51, plastoquinone-9; 52, ubiquinone (Coenzyme Q); 53, menaquinone (vitamin K2). ChlP, geranylgeranyl reductase; ChlG, chlorophyll synthase; HPT, homogentisate phytyltransferase; VTE1, tocopherol cyclase; GTMT, tocopherol methyltransferase; MenA, dihydroxynaphthoate octaprenyltransferase; UbiE, phylloquinone / menaquinone methyltransferase; HST, homogentisate solanesyltransferase; VTE3, methylsolanyl-benzoquinone methyltransferase (not found); Coq2, 4-hydroxybenzoate hexaprenyltransferase; Coq3, hexaprenyldihydroxybenzoate methyltransferase (two methylation reactions); Coq6, ubiquinone biosynthesis monoonxygenase; Coq5, ubiquinone biosynthesis methyltransferase; Coq7, ubiquinone biosynthesis monooxygenase. Terpenoid side chains are highlighted in gray.

Meroterpenoid quinones play indispensable roles as electron carriers in photosynthesis in the chloroplast and oxidative phosphorylation in the mitochondria. The terpene chains of these molecules, as well as that of the photosynthetic pigment chlorophyll, are derived from polyprenyl diphosphates. The C_20_ prenyl diphosphate geranylgeranyl diphosphate (**17**) is reduced to phytyl diphosphate (**47**, Figure 10) by geranylgeranyl reductase (ChlP, E.C. 1.3.1.83) represented by three nonredundant curated contigs in the Showa transcriptome. One of these contigs, 30757, features high sequence coverage and the encoded protein is predicted to be directed into the chloroplast, as expected (Additional file 1: Table S11). **47** is the precursor for the phytyl side chain of prenylated proteins and that of chlorophylls (**48**). A single curated contig with moderate sequence coverage encodes the putative prenyl transferase chlorophyll synthase (ChlG, E.C. 2.5.1.62) that attaches the phytyl side chain onto chlorophyllide-a to yield **48**. **47** is also the precursor for the terpenoid side chains of α-tocopherol (vitamin E, **49**) and phylloquinone (vitamin K1, **50**). The vitamin E family of antioxidants including α-tocopherol (**49**) contributes to the integrity of photosynthetic membranes and may influence plant responses to many physiological stressors [91]. Phylloquinone (vitamin K1) is present in all photosynthetic plants as a cofactor for photosystem-I-mediated electron transport [92]. All the enzymes necessary for the biosynthesis of vitamin E from **47** and homogentisate, and vitamin K1 from **47** and 1,4-dihydroxy-2-naphthoate are predicted to be represented in the Showa transcriptome by curated contigs of low to moderate sequence coverage, some of them (VTE1, GTMT, and UbiE, Additional file 1: Table S11) encoded as multiple isozymes. The polyprenyl diphosphates **18** and **19**, derived from geranyl diphosphate (**15**) and geranylgeranyl diphosphate (**16**) provide the terpene side chains of the electron carriers plastoquinone-9 (**51**) in the chloroplast, and ubiquinone (**52**) and menaquinone in the mitochondria (**53**, Figure 10) [93]. Proteins similar to the enzymes necessary for the biosynthesis of plastoquinone from **18** and homogentisate, and for the production of coenzyme Q (ubiquinone **52**) and vitamin K2 (menaquinone **53**) from 4-hydroxybenzoate and 1,4-dihydroxy-2-naphthoate, respectively, and from **18** and **19** are encoded in the Showa transcriptome by curated contigs of low to moderate sequence coverage, many as multiple isozymes (Additional file 1: Table S11). However, we could not identify contigs for methylsolanyl-benzoquinone methyltransferase (VTE3) for **51** biosynthesis, and a presumed ubiquinone biosynthesis methyltransferase (Coq5, E.C. 2.1.1.201) is encoded only by a single curated contig of predicted fungal origin (Additional file 1: Table S11).

The diterpenoid growth hormones gibberellic acids [94] are derived from geranylgeranyl diphosphate (**17**) by cyclization followed by multiple oxidations in land plants. There are no unequivocal reports on the production of gibberellins in green algae [95], nor have genes with high similarity to key gibberellin production pathways been located in genome sequences [15]. Fittingly, the first enzyme of the gibberellic acid pathway that generally harbours both *ent-*copalyl diphosphate synthase (E.C. 5.5.1.13) and *ent-*kaurene synthase (E.C. 4.2.3.19) activities could not be identified in the *B. braunii* Showa transcriptome. On the other hand, curated contigs that may encode enzymes for the oxidative processing of the tetracyclic intermediate *ent*-kaurene to gibberellin A4 are present in the transcriptome at low sequence coverage (Additional file 1: Table S12).

### *S*-adenosylmethione regeneration

In addition to the biosynthesis of many primary metabolites of terpenoid or other origin, the production of large amounts of higher botryococcenes and methylated squalenes (Figure 7) in *B. braunii* Showa requires a robust supply of *S*-adenosylmethionine (SAM) that is used as a donor for methylation reactions. Transfer of the methyl group of SAM to the substrate by these methyltransferases yields *S*-adenosylhomocysteine that is hydrolysed by *S*-adenosyl-L-homocysteine hydrolase (AhcY, E.C. 3.3.1.1) to homocysteine and adenosine (Additional file 1: Table S13). Homocysteine is methylated by 5-homocysteine *S*-methyltransferases, including the *S*-methylmethionine-dependent MmuM (E.C. 2.1.1.10), the 5-methyltetrahydrofolate- and cobalamin-dependent MetH (E.C. 2.1.1.13), and the 5-methyltetrahydropteroyl-triglutamate-utilizing but cobalamin-independent MetE (E.C. 2.1.1.14), yielding L-methionine. Finally, *S*-adenosylmethionine synthase (MetK, E.C. 2.5.1.6) transfers the adenosyl moiety of ATP to methionine, yielding SAM and releasing phosphate and pyrophosphate. Machine-assembled contigs for all these deduced enzymes have been identified in the Showa transcriptome, with low (MetK, MmuM), low to moderate (MetH) and high to extremely high sequence coverage (AhcY, MetE, Additional file 1: Table S13).

### Competing storage compounds: biosynthesis of triacylglycerols

Photosynthetic carbon and energy intended for storage is partitioned in *B. braunii* Showa amongst terpenoid hydrocarbons, triacylglycerols (TAGs), and carbohydrates. We have generated inventories for machine-assembled contigs predicted to encode crucial enzymes in hydrocarbon-competing storage compound biosynthetic pathways.

**Figure 11:**
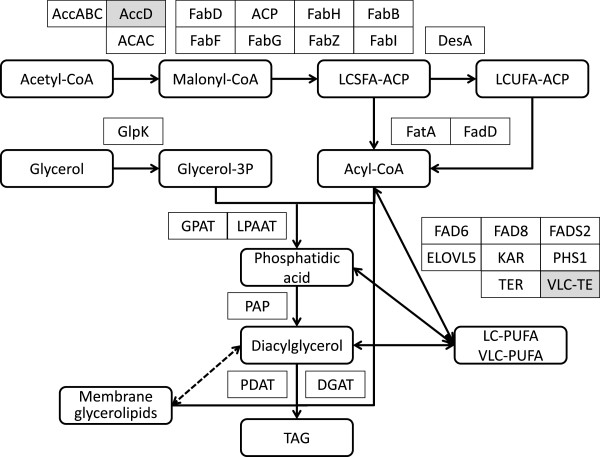
**Reconstruction of triacylglycerol biosynthesis in *B. braunii.*** Deduced enzymes are shown in white boxes, enzymes not represented in the transcriptome of the Showa strain of *B. braunii* are in grey boxes. AccABC, acetyl-CoA carboxylase, subunits A, B, and C; AccD, acetyl-CoA carboxylase, subunit D; ACAC, multifunctional protein acetyl-CoA carboxylase; FabD, ACP:*S*-malonyltransferase; ACP, acyl carrier protein; FabH, β-ketoacyl:ACP synthase III; FabB, β-ketoacyl:ACP synthase I; FabF, β-ketoacyl:ACP synthase II; FabG, β-ketoacyl:ACP reductase; FabZ, β-hydroxyacyl:ACP dehydratase; FabI, enoyl-ACP reductase; DesA, stearoyl-ACP δ-9 desaturase; FatA, oleoyl-ACP hydrolase; FadD, long chain fatty acid:CoA ligase; GlpK, glycerol kinase; GPAT, glycerol-3-phosphate *O*-acyltransferase; LPAAT, lysophosphatidic acid acyltransferase; PAP, phosphatidic acid phosphatase; DGAT, diacylglycerol acyltransferase; PDAT, phospholipid:diacylglycerol acyltransferase; FAD6, Δ-12 fatty acid desaturase; FAD8, Δ-15 fatty acid desaturase; FADS2, Δ-6 fatty acid desaturase; ELOVL5, very long chain fatty acid elongase; KAR, β-ketoacyl-CoA reductase; PHS1, 3-hydroxyacyl-CoA dehydratase; TER, enoyl-CoA reductase, VLC-TE, very long chain fatty acyl-CoA hydrolase. CoA, coenzyme A; LCSFA, long chain saturated fatty acid (C16-18:0); LCUFA, long chain unsaturated fatty acid (C16-18:1 n-9); LC-PUFA, long chain polyunsaturated fatty acid (C16-18:2–4); VLC-PUFA, very long chain polyunsaturated fatty acid (C20-24:2–4), TAG, triacylglycerol.

Fatty acid biosynthesis for polar membrane lipids (various glycosyl-glycerolipids and phosphoglycerolipids) and neutral TAGs in microalgae like *B. braunii* primarily occur in the chloroplast (with limited synthesis also occurring in the mitochondria, [96]) by a type II (multiprotein complex) fatty acid synthase (FAS) enzyme system. Malonyl-CoA, the substrate for FAS, is derived from the primary metabolite acetyl-CoA by the biotin-containing multienzyme complex acetyl-CoA carboxylase (Acc, E.C. 6.4.1.2) in the chloroplast (Figure 11) [19,97]. Using ATP, the biotin carboxylase subunit AccC carboxylates biotin on the biotin carboxyl carrier protein AccB, followed by the transfer of the carboxyl moiety to acetyl-CoA by the carboxyl transferase subunit AccA and AccD. Contigs of moderate to high sequence coverage encode the presumed AccA, B and C (Additional file 1: Table S14), while contigs for AccD (carboxyl transferase β-subunit) was missing from the Showa transcriptome as expected for a plastid-encoded gene [98]. A multifunctional, most likely cytosolic Acc isozyme (ACAC, E.C. 6.3.4.14) [19,98] is encoded by multiple contigs with various sequence coverage. Malonate from malonyl-CoA is transferred to the acyl carrier protein (ACP1 and ACP2) of the type II FAS by ACP *S*-malonyltransferase (FabD, E.C. 2.3.1.39) [23,97]. Malonyl-ACP is the acyl donor for the subsequent recursive, decarboxylative Claisen condensations catalysed by the β-ketoacyl:ACP synthase components of the type II FAS, generating the growing fatty acyl chain intermediates that remain bound to the ACP as thioesters. KAS III (β-ketoacyl:ACP synthase III, FabH, E.C. 2.3.1.180) conducts the first condensation using acetyl-CoA and malonyl-ACP. Acyl-ACP (C_4_-C_14_) serves as the acceptor for further condensations with malonyl-ACP as the donor to produce long chain fatty acids (most frequently palmitic acid, C16:0) by KAS I (β-ketoacyl:ACP synthase I, FabB, E.C. 2.3.1.41). The final condensation to yield the long chain fatty acid stearic acid (C18:0) is catalysed by KAS III (β-ketoacyl:ACP synthase II, FabF, E.C. 2.3.1.179) [23]. Each condensation cycle also involves three consecutive reductions to yield the fully saturated fatty acyl-ACP from the nascent β-ketoacyl-ACP. These steps are catalysed by the NADPH-dependent β-ketoacyl-ACP reductase (FabG, E.C. 1.1.1.100), β-hydroxyacyl-ACP dehydratase (FabZ, E.C. 4.2.1.-), and the NADH or NADPH-dependent enoyl-ACP reductase (E.C. 1.3.1.-). Desaturation to generate long chain fatty acids with n-9 *cis* double bonds is carried out at the acyl-ACP thioester stage by stearoyl-ACP Δ^9^ desaturase (DesA, E.C. 1.14.19.2) [23], yielding palmitoleic or more frequently oleic acid (C16:1 n-9 and C18:1 n-9, respectively). Long chain acyl-ACPs may be utilized for a direct transfer of the acyl group to afford phosphatidic acid towards the synthesis of various polar membrane glycerolipids in the plastid. Alternatively, long chain acyl-ACPs are hydrolysed by thioesterases (including oleoyl-ACP hydrolase [FatA, E.C. 3.1.2.14]) to release free fatty acids that are exported from the plastid. These fatty acids are reactivated as CoA thioesters by long chain fatty acid:CoA ligases (FadD, E.C. 6.2.1.3) in the cytosolic face of the ER for the biosynthesis of various lipids [19] (Figure 11). Multiple contigs for two deduced isoforms of ACP, and a full contingent of predicted enzymes for a type II FAS, a Δ^9^ desaturase, a thioesterase and fatty acyl CoA ligases have been identified in the Showa transcriptome ( Additional file 1: Table S14), some with very high sequence coverage (contig 35624 with 725.5 reads/kb for an ACP, and contig 43176 with 378.2 reads/kb for a Δ^9^ desaturase). A short contig (FX085405) for FabB from the race B strain *B. braunii* BOT-22 shows 98% identity to the FabB encoded by contig 14404 of the Showa strain [32]. Interestingly, contigs with low sequence coverage, coding for assumed animal and fungal Type I (multifunctional enzyme) FASs have also been identified in the Showa transcriptome. Based on the high similarities of the encoded proteins to animal or fungal enzymes, and the divergent codon usage of the contigs, the corresponding transcripts may have originated from other organisms present in the non-axenic culture.

Long chain fatty acids may undergo further desaturations and chain elongations to afford long chain and very long chain polyunsaturated fatty acids (LC-PUFA and VLC-PUFA, respectively, Figure 11). Oxygen-dependent Ω^6^ (Δ^12^) fatty acid desaturases (FAD6, E.C. 1.14.19.-) and Ω^3^ (Δ^15^) fatty acid desaturases (FAD8, E.C. 1.14.19.-) in the chloroplast and the endoplasmic reticulum generate linoleic acid (C18:2 n-6) and α-linolenic acid (C18:3 n-3) and their longer chain equivalents, respectively, using acyl-glycerolipid substrates [23,99,100]. Both of these predicted enzymes are encoded in the Showa transcriptome at low to moderate sequence coverage. An apparent front-end desaturase, FADS2 (Δ^6^ fatty acid desaturase, E.C. 1.14.19.-) that may yield stearidonic acid (C18:4 n-3) or its longer chain equivalents, perhaps by using acyl-CoA esters [19], is also present in the transcriptome with low sequence coverage. For VLCFA and VLC-PUFA, recursive cycles of 2-carbon additions from malonyl-CoA and the following three desaturation steps outside of the chloroplast (probably in the microsomes) parallel that of *de novo* long-chain fatty acid biosynthesis in the chloroplast, but without the involvement of acyl carrier proteins [19]. Thus, multiple contigs with moderate to high sequence coverage that encode putative very long chain fatty acid elongase (ELOVL5, E.C. 2.3.1.-), β-ketoacyl-CoA reductase (KAR, E.C. 1.1.1.-), 3-hydroxyacyl-CoA dehydratase (PHS1, E.C. 4.2.1.-), and enoyl-CoA reductase (TER, E.C. 1.3.1.-) enzymes have been found in the Showa transcriptome. However, a very long chain fatty acyl-CoA hydrolase (E.C. 3.1.2-) that may act as a thioesterase has not been identified. VLCFA, VLC-PUFA and hydrocarbon pyrolysis products of these have been observed in *B. braunii*, albeit primarily in the A race strains [80,101-103].

Triacylglycerol storage lipids are assembled in the endoplasmic reticulum by two sequential acylations of *sn*-glycerol-3-phosphate, followed by dephosphorylation and a final acyl transfer (Figure 11). Exchange of acyl chains amongst TAG, glycerolipids and the acyl-CoA pool (acyl editing) provides flexibility to channel carbons for storage or for functional lipid biosynthesis [23]. Following the ATP-dependent phosphorylation of glycerol by glycerol kinase (GlpK, E.C. 2.7.1.30), glycerol-3-phosphate *O*-acyltransferase (GPAT, E.C. 2.3.1.15) and lysophosphatidic acid acyltransferase (LPAAT, E.C. 2.3.1.51) generate phosphatidic acid using the acyl-CoA pool (Additional file 1: Table S14). Phosphatidic acid phosphatase (PAP, E.C. 3.1.3.4) affords *sn-*1,2-diacylglycerol, to be acylated by diacylglycerol acyltransferase (DGAT, E.C. 2.3.1.20) to produce TAG. Diacylglycerols are also the precursors for the various polar lipids (glycosyl-glycerolipids and phosphoglycerolipids), while phospholipid:diacylglycerol acyltransferase (PDAT, E.C. 2.3.1.158) shuttles acyl groups amongst phosphoglycerolipids, betaine lipids, and TAG in an acyl-CoA independent process [19,97,103]. All these key TAG biosynthetic enzymes are predicted to be present in the Showa transcriptome with low sequence coverage ( Additional file 1: Table S14) which may be reflective the low level of TAGs predicted to be present in the B race of *B. braunii*[79].

In land plants, TAGs accumulate in oil bodies whose lipid/water interface features the structural proteins oleosins. Hydrophobic proteins (MLDP, major lipid droplet protein) that may be functionally equivalent to, but not structurally similar to plant oleosins have recently been identified by proteomic approaches in the oil bodies of Chlorophyta algae [20,104]. Multiple contigs encoding presumed MLDPs are also featured in the Showa transcriptome, some with very high sequence coverage (contigs 0772, 35177 and 42893, with 237–295 reads/kb) that may indicate active transcription of the corresponding genes.

### Competing storage compounds: biosynthesis of starch and other carbohydrates

A major sink for photosynthetic carbon intended for storage in algae are polysaccharides including starch. The biosynthesis of these compounds competes with those of hydrocarbon oils and TAG lipids in *B. braunii*, thereby reducing biofuel yield. On the other hand, starch and cellulosic biomass, after hydrolysis, may be utilized as a feedstock for the fermentative production of biofuel using non-photosynthetic microorganisms [105,106].

Starch biosynthesis in the chloroplast is initiated by the phosphorylation of α-D-glucose at the C6 position by hexokinase (HK, E.C. 2.7.1.1) and glucokinase (Glk, E.C. 2.7.1.2) in ATP-dependent reactions. D-glucose 6-phosphate is then converted by phosphoglucomutase (PGM, E.C. 5.4.2.2) to α-D-glucose 1-phosphate that serves as a substrate for the ATP-dependent glucose-1-phosphate adenylyltransferase (GlgC, E.C. 2.7.7.27), a major rate-controlling enzyme of the pathway in plants and bacteria [107]. The resulting ADP-glucose is then polymerized by starch synthase (GlgA, E.C. 2.4.1.21) to generate amylose with linear α-1,4-glycosidic linkages. Branched α-1,6 glycosidic linkages between α-1,4-glucan chains is generated by the 1,4-α-glucan branching enzyme (GlgB, E.C. 2.4.1.18) to yield water-insoluble amylopectin. Conversely, amylo-α-1,6-glucosidase (AGL, E.C. 3.2.1.33) hydrolyses α-1,6 glycosidic linkages to limit branching and to mobilise glucose from starch. Multiple contigs with low to moderate sequence coverage encoding these predicted enzymes are present in the Showa transcriptome, with the deduced glycogen debranching enzyme AGL encoded by a single contig that might have originated from a fungal cohabitant (Additional file 1: Table S15).

Considering that cellulose is a major constituent of the cell wall in green algae (up to 80% in *Chlorella* sp., [108]), the biosynthesis of this 1,4-β-D-glucan presents a major demand from the available photosynthetic carbon. Since Chlorophyta cell walls do not contain lignin [109,110], cellulose from the biomass of these algae may provide a relatively more easily accessible source of sugar for the production of biofuels by fermentation. For the biosynthesis of cellulose, α-D-glucose 1-phosphate is activated by uridylation by UTP:α-D-glucose-1-phosphate uridylyltransferase (UGP, E.C. 2.7.7.9). The resulting UDP-glucose is the substrate for cellulose synthase (BcsA, E.C. 2.4.1.12). Orthologous genes are present in single copies in green algae [111]. Contigs with low to moderate sequence coverage have been identified for these two inferred enzymes in the *B. braunii* Showa transcriptome (Additional file 1: Table S15).

### Localization of liquid terpenoid compounds into the extracellular matrix

Apart from the biosynthesis of hydrocarbons with fuel characteristics and utility resembling that of fossil crude oil, *B. braunii* is also remarkable in depositing these compounds into a communal extracellular matrix that holds the colony together. Forming a fibrous network, this matrix consists of highly complex cross-linked polymers, originating from methylated squalenes and/or very long chain fatty acids [9]. The hydrocarbons initially accumulate within the cells as oil bodies, but the large majority (95%) of the extractable liquid oils are found in the matrix [9]. Traffic of these hydrocarbons seems to coincide with maturation of botryococcenes to C_34_ and higher homologues in *B. braunii*, race B [13,75]. While the exact mechanism of the excretion of hydrocarbons into the extracellular space is unknown, the characterization and subsequent engineering of this trait into other (micro)organisms holds great promise for relieving product toxicity and simplifying extraction of advanced biofuels from biomass.

Efflux pumps [112] are one of the obvious candidates for the cellular export system of hydrocarbons. The Showa transcriptome contains numerous contigs encoding potential ABC (ATP-binding cassette) transporters (also known as multidrug-resistance-related proteins, MRPs) that mediate ATP-dependent transport of a bewildering array of molecules across organellar and cellular membranes (Additional file 1: Table S16). Plant ABC transporters fall into several subfamilies with varied (and often uncharacterized) functions but with a substantial functional redundancy of the family members within a single organism [113]. Subfamily A members take part in the transport of various lipids including sterols and lipoproteins across membranes in animals. Although members of this subfamily have been identified in plants, their functions have not been characterized. Subfamily B members are multidrug resistance factors that take part in auxin, secondary metabolite, and xenobiotic traffic in plants, with mitochondrion-located members involved in iron-sulphur cluster trafficking. Subfamily C members play a role in detoxification and in vacuolar transport of glucuronides, chlorophyll degradation products and anthocyanins. Subfamily D members are essential for the import of VLCFA into the peroxisomes for β-oxidation. Subfamily G transporters are involved in the export of alkanes and other lipids that form the waxy cuticle and in the secretion of volatile compounds in flowers and roots of plants. Other group members convey resistance to various substances including terpenoids and herbicides [113,114]. Subfamily G is generally expanded in plants compared to animals [114], with a large number of transcripts encoding presumed members of this subfamily also present in the Showa transcriptome. The proteins represented by these contigs, and to a lesser extent those of putative Subfamily D and A members (Additional file 1: Table S16), are prime candidates for the role of a liquid hydrocarbon exporter in *B. braunii* race B strains including Showa.

**Figure 12:**
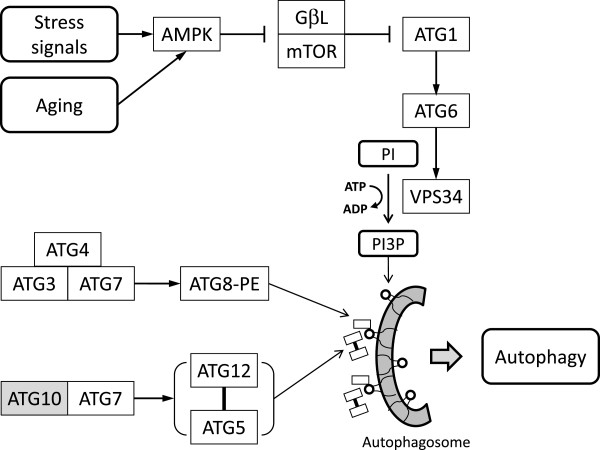
**Reconstruction of autophagy regulation in *B. braunii.*** Deduced enzymes are shown in white boxes, enzymes not represented in the transcriptome of the Showa strain of *B. braunii* are in grey boxes. AMPK, 5'-AMP-activated protein kinase, catalytic alpha subunit; GβL, mTOR associated protein; mTOR, FKBP12-rapamycin complex-associated protein; ATG1, unc51-like kinase; ATG6, Beclin1; VPS34, phosphatidylinositol 3-kinase; ATG3, -4, -5, -7, -10, -12, autophagy-related proteins; ATG8-PE, phosphatidylethanolamine-modified autophagy-related protein 8. PI, 1-phosphatidyl-D-myo-inositol; PI3P, 1-phosphatidyl-D-myo-inositol 3-phosphate.

Programmed cell death may be an alternative or auxillary mechanism to release liquid hydrocarbon products from intracellular vesicles into the extracellular matrix, or to generate the outer matrix-derived “cell cap” material that covers the outer edge of the *B. braunii* cells in the colony [115] . However, given the large amount of liquid hydrocarbon in the extracellular matrix and that we infrequently see dead cells of *B. braunii* Showa within a colony that is rapidly accumulating extracellular hydrocarbons, the contribution of cell death to the extracellular localization of these compounds may only be limited. While caspase-mediated apoptotic Type 1 cell death pathway transcripts are only sporadically represented in the Showa transcriptome, a significant number of contigs encode putative proteins related to the Type 2 (autophagic) cell death pathway (Additional file 1: Table S17). Autophagy recycles cellular constituents (from cytosolic macromolecules to whole organelles) by proteolytic degradation in response to cell aging or various stress conditions including nutrient deprivation and oxidative stress. Autophagy plays a housekeeping role and is one of the coping mechanisms of the cell, and its deficiencies were linked to several diseases in mammals [116]. Over-activation of autophagy on the other hand promotes cell death in a caspase-independent pathway or by a complex interplay with apoptosis [117]. Autophagy pathways are evolutionarily conserved, and have also been characterized in green algae including *C. reinhardtii*[118,119]. Stress signals are transmitted by 5'-AMP-activated protein kinase (AMPK, E.C. 2.7.11.11) towards the FKBP12-rapamycin complex-associated protein (mTOR) and the mTOR associated protein (GβL), causing de-suppression of autophagy (Figure 12). Activation of autophagy-related protein 1 (unc51-like kinase ATG1, E.C. 2.7.11.1) and Beclin 1 (VPS30 or ATG6) activates the ATP-dependent phosphatidylinositol 3-kinase (VPS34, E.C. 2.7.1.137) that phosphorylates 1-phosphatidyl-1D-myo-inositol. The resulting 1-phosphatidyl-1D-myo-inositol 3-phosphate recruits further ATG proteins to the phagophore membrane. These include ATG8-phosphatidylethanolamine (itself generated by ATG4, ATG3 and ATG7), and the complex of ATG5 and ATG12 whose conjugation is catalysed by ATG7 and ATG10. The binding of these complexes facilitates selection of cargo to be degraded, and leads to the expansion of the autophagosome and its fusion with the vacuolar membrane (Figure 12) [116,119]. Contigs with low to moderate sequence coverage encoding predicted autophagy pathway proteins are present in the Showa transcriptome (Additional file 1: Table S17). Further experiments are necessary to determine whether this reconstructed autophagy pathway contributes to hydrocarbon excretion in *B. braunii* Showa, or whether its presence in the transcriptome simply reflects the abundance of cells that are in stationary phase approaching senescence and may be stressed by nutrient limitation in the long term culture.

## Conclusions and outlook

Functional annotation of the transcriptome of the *B. braunii* race B, strain Showa uncovered diverse biological processes operational in this hydrocarbon-producing green alga. Global comparisons with other algal transcriptomes reveal similar degrees of annotation coverage, with the number and the range of annotations supporting a strong degree of conservation between algal genomes and transcriptomes. The level of annotation coverage in *B. braunii* is supported by the identification of the majority of predicted enzymes within diverse biological pathways, for example within those related to the production of terpenes and lipids.

This study describes reconstituted metabolic pathways related to the biosynthesis of terpenoid hydrocarbons in the non-model green microalga *B. braunii* Showa, following the fate of photosynthetic carbon from 3-phosphoglycerate to the general terpenoid precursors IPP and DMAPP, the production of linear polyprenyl backbones, the biosynthesis of triterpenoids, the decoration/tailoring of botryococcene and squalene to yield liquid hydrocarbon compounds and matrix structural materials, and possible routes for the extracellular localization of these compounds. A recurrent theme is the expansion of particular gene families. This allows the adaptation of the paralogs to structurally orthogonal substrates (botryococcene methyltransferases), and permits neofunctionalization to support novel biochemical reactions (botryococcene synthase). Paralogs may yield increased metabolic flux, or may provide additional flexibility in terms of regulation, compertmentalization, and biochemical properties (deoxyxylulose phosphate synthase, and possibly ABC transporters for hydrocarbon export). Metabolic pathways leading to other terpenoids have also been reconstructed, and anabolic pathways for competing storage compounds (TAG and polysaccharides) were similarly mapped.

The reconstructed metabolic networks, their participating enzymes and the corresponding cDNA sequences provide a genetic and metabolic framework that should empower biosynthetic engineering approaches targeting the increased production of hydrocarbons in *B. braunii*, or the mobilization of these pathways into genetically tractable photosynthetic (algal or land plant) hosts or heterotrophic microbial strains [50,120-122]. In particular, increasing the flux of photosynthetic carbon towards terpene precursors in the chloroplast or the cytosol of *B. braunii* by transplanting the cytoplasmic mevalonate pathway, or by fine tuning the expression of DXR (deoxyxylulose phosphate reductase) and Idi (isopentenyl-diphosphate delta-isomerase) may be interesting approaches [60,120,123]. Further experiments should investigate the importance of the pentose phosphate cycle as an anaplerotic pathway feeding into the MEP/DOXP pathway, and clarify the actual metabolite(s) and enzyme(s) connecting these two routes [62]. Conversely, tuning down, or blocking the biosynthesis of competing storage carbons (like that of starch in *Chlamydomonas*[20,124,125]) may also increase the accumulation of liquid hydrocarbons. Varying the level and timing of the expression for the two-component botryococcene synthase [34,38] and various storage hydrocarbon decorating enzymes (including the recently described methyltransferases for C_32_ triterpenoids [33], the still unknown methyltransferases for C_34_ triterpenoids, and the various oxidases that yield oxidized (methyl)squalenes, ether polymers and matrix materials) should help to tailor liquid hydrocarbon biosynthesis for particular biofuel applications. Investigations into the export of hydrocarbons into the extracellular matrix of *B. braunii* should uncover a particularly valuable trait that may have a widespread application to increase yield, reduce end product toxicity and simplify product recovery during biofuel manufacture by fermentation. Interesting questions are presented by the interconnection of the biosyntheses of terpenoid hydrocarbons and very long chain fatty acids with the formation of the extracellular matrix materials in *B. braunii* that provide the building blocks for an extracellular carbon storage organ, but also the physical basis for colony organization in this organism. Further transcriptomic, proteomic, metabolomic, and metabolic flux analyses that compare varied growth conditions influencing hydrocarbon accumulation would shed light on the regulatory networks and pathway interactions channeling carbon and energy flow in algal cells.

In addition to biofuel biosynthetic pathway discovery, the integrated data-mining environment offered by the publicly available web annotation tool described here allows researchers to query the *B. braunii* transcriptome for any specific transcript sets to rapidly and efficiently extract biologically relevant information related to different contexts. As the transcriptome dataset is revised and supplemented with addtional manually curated annotations, the most current functional data will be made available to the community via the public portal and annotation tool.

## Methods

### Algal cell cultures

A near axenic culture of *B. braunii* B race, Showa strain (a.k.a the Berkeley strain) [126] was obtained as previously described [36]. Briefly, cultures were diluted with sterilized media, individual colonies were isolated under a microscope, and transferred to fresh, sterile media for growth. The resulting cultures were grown in modified Chu 13 media [66] at 22.5°C under a 12:12 light:dark (L:D) cycle using 13 W compact fluorescent 65 K light bulbs at a distance of 7.62 cm, which produced a light intensity of 280 μmol photons · m^-2^ · s^-1^. The cultures were continuously aerated with filter-sterilized, enriched air containing 2.5% CO_2_. Fifty milliliters of culture was used to inoculate 750 ml of subsequent subcultures every 4 weeks. Bacterial contamination of the cultures was routinely monitored by inoculating LB plates with 100 μl of undiluted media from a *B. braunii* culture that has had the algae removed by filtration (see below), and incubating the plates at 37°C for 4 days. This routinely yields an average of 5 bacterial colonies.

### Sample collection and RNA isolation

*B. braunii* samples for total RNA isolation were collected at seven time points (days 0, 3, 5, 8, 14, 18, and 22) representing the four-week culture cycle. For days 0, 3, and 5, cells were collected from a full culture flask (750 ml) each, yielding a biomass of approximately 0.5 g (days 0 and 3) and 1 g (day 5) of wet cell weight. For all other time points, one half of a flask culture (375 ml) was harvested, yielding a biomass of approximately 1.5 g (day 8) and 3 g (day 22) of wet cell weight. Thus, a total of 5 separate culture flasks were used for the biomass collection (1 flask each for days 0, 3, and 5; 1 flask for days 8, and 14; one flask for days 18 and 22), and each of these 5 flasks originated from a single 4-week-old *B. braunii* culture. Samples were harvested by vacuum filtration using 35 μm nylon mesh (Aquatic Ecosystems Inc., Apopka, FL). The harvested colonies were rinsed with sterilized deionized water, frozen in liquid nitrogen, and stored at −80°C for RNA extraction.

Total RNA from each sample was extracted by pulverizing liquid nitrogen-frozen algae using a Tissuelyser II (Qiagen, Valencia, CA). Approximately 200 mg of the pulverized frozen tissue was added to 1 ml of TRIzol reagent (Life Technologies, Grand Island, NY) and total RNA was extracted following the protocol of the manufacturer. The resulting RNA pellet was washed with 75% EtOH, dried in a SpeedVac, and the RNA was selectively precipitated from contaminating polysaccharides by resuspending the RNA in 500 μl of 2M LiCl and incubating at room temperature for 5 min. The sample was centrifuged at 12,000 x *g* for 15 min to pellet the RNA and the supernatant (containing the polysaccharides) was discarded. The LiCl precipitation steps were repeated until the size of the RNA pellet appeared constant. The final RNA pellet was resuspended in 1x TE, extracted against an equal volume of phenol/CHCl_3_/isoamyl alcohol (25/24/1) and centrifuged at 10,000 x *g*. The aqueous supernatant containg the RNA was extracted with CHCl_3_ and centrifuged at 10,000 x *g*. The RNA in the aqueous supernatant was then precipitated by adding 0.1 volumes of 3M sodium acetate and 2.5 volumes 100% EtOH, incubating at −20°C for 20 min, and centrifuging at 10,000 x *g* for 15 min. The RNA pellet was washed with 70% EtOH, dried, and dissolved in 50 μl RNase free dH_2_O. The RNA was quantified by absorbance at 260 nm (A_260_), and the A_260_/A_280_ (protein contamination) and A_260_/A_230_ (polysaccharide contamination) ratios were determined. An A_260_/A_280_ ratio of 1.8 or higher and an A_260_/A_230_ ratio of 2.0 or higher were deemed as suitable. The later time points (days 18 and 22) contained higher amounts of polysaccharides that were not completely removable by repeated LiCl precipitation and the A_260_/A_230_ ratios for these samples were in the range of 1.5 to 1.6. The RNA from each sample was then pooled into one sample and treated at 37°C for 30 min with 1 unit of RQ1 RNAse-free DNAse (Promega, Fitchburg, WI) for each μg of RNA. After phenol/CHCl_3_/isoamyl alcohol and subseaquent CHCl_3_ extractions, the RNA was again precipitated with 3M sodium acetate/100% EtOH as above. The resulting RNA was resuspended in 100 μl RNAse-free dH_2_O and the RNA quantified and protein and polysaccharide contamination determined as above. Finally, the RNA was sent to the DOE Joint Genome Institute (JGI) for library construction and 454 sequencing.

### RNA quality assessment

In addition to the A_260_/A_280_ and A_260_/A_280_ ratios, the quality of the isolated RNA was analyzed by several methods. The RNA from days 0, 3, and 5 were analyzed by RT-PCR for the presence of several cDNAs including squalene synthase (SS) [37], squalene synthase-like-1 (SSL-1) [34], β-actin [36], ferredoxin-1, and ferredoxin NADPH reductase ( Additional file 1: Figure S2). The pooled RNA was analyzed for the presence of active nucleases by incubating one 5-μg aliquot of RNA at 37°C and one 5-μg aliquot of RNA at 0°C for 1 hr, followed by visual inspection for degradation of the RNA on a denaturing agarose gel.

### Sequence assembly

The data were assembled using an in house cleaning, clustering, and assembling pipeline developed for 454 transcriptome sequences [39]. This pipeline uses a combination of assembly methods, which has been shown to produce assemblies that are superior to any single method, resulting in fewer contigs with shorter cumulative length [40]. Raw reads were first cleaned using SnoWhite v1.1.3 (http://dlugoschlab.arizona.edu/software.html) with the TagDust cleaning step implemented [127]. The cleaned reads were clustered using the *wcd* EST clustering tool with an error threshold value of 5 [128]. Based on the *wcd* output, a perl script split the dataset into multiple fasta and associated quality files based on cluster sizes. These cluster sizes correspond to read depth (i.e. expression level) allowing the assembly parameters to be tailored for the expression level of transcripts. In this way, transcript contigs are initially assembled in separate bins according to their expression level, ameliorating the effects of large differences in sequence representation in transcriptome datasets. The multiple files were assembled separately using Mira v3.0.0 [129]. The Mira outputs were assembled a second time using CAP3 with an overlap percent identity cutoff of 94 [130], creating an “assembly of assemblies”. Coverage and read depth in this final assembly were determined by mapping the reads to the assembly in CLC Genomics Workbench (v4.9). The quality of the assembly was benchmarked against the core set of eukaryotic genes using the Core Eukaryotic Genes Mapping Approach (CEGMA) algorithm [41]. Transcript contigs were deposited into the GenBank Transcriptome Shotgun Assembly Sequence Database and were assigned accession numbers KA089548 - KA133805 and KA659919 - KA660048. The number of contigs that might have originated from transcripts of other organisms were estimated using the Metagenomics RAST server [48] from the taxonomical distribution of the top BlastX hits to proteins in GenBank with the transcripts of the *B. braunii* Showa database.

### The *Botryococcus braunii* web-based annotation tool and data depository

Contigs were annotated using Blast2GO to assign Gene Ontology classifications [131]. KEGG annotation was done with the KAAS annotation server (http://www.genome.jp/tools/kaas/) using the bi-directional best hit method [132]. Custom Perl sripts (avalible from JDH by request) were used to generate the Venn diagram in Figure 3 to compare KEGG annotations for Chlorophyta genomes and the *B. braunii* transcriptome, using predicted proteins from the genomes of *Chlamydomonas reinhardtii* (v3.0, http://genome.jgi-psf.org/Chlre3/Chlre3.download.ftp.html), *Chlorella variabilis* NC64A (http://genome.jgi-psf.org/ChlNC64A_1/ChlNC64A_1.download.ftp.html) and *Micromonas* sp. RCC299 (v3.0, http://genome.jgi-psf.org/MicpuN3/MicpuN3.download.ftp.html).

To assign pathway annotations, unique transcript sequences were aligned using the BlastX alignment program to a target database containing KEGG proteins with pathway annotations as described in [28]. Alignments were filtered using an E-value threshold of 10^-5^, and the top KEGG protein hit for each transcript meeting the threshold was kept. KEGG pathways assigned to the protein in the target database were subsequently assigned to the corresponding transcripts. MetaCyc, Panther, and Reactome pathways were assigned by performing BlastX alignments against UniProt entries with annotations in the respective databases and filtering using an E-value threshold of 10^-5^. Gene Ontology, MapMan Ontology, and KOG annotations were assigned by identifying orthologous proteins in the model alga *C. reinhardtii* and the plant *Arabidopsis thaliana* using reciprocal BlastX and tBlastN alignments and keeping alignments that were pairwise best hits. Annotations assigned to orthologous proteins in those organisms were subsequently transferred to *Botryococcus* transcripts. Pfam annotations were assigned using the publicly available web-based batch search feature of the Pfam database.

### Manual curation of selected transcripts and pathways

Contigs with automated annotations related to terpenoid biosynthesis were extracted from the dataset and confirmed by reciprocal BlastX searches using the non-redundant protein sequences at NCBI as comparators. Further contigs representing target genes were collected by exhaustive Blast searches of the *B. braunii* Showa contig and singleton databases using BlastStation-Free v 1.1 (TM Software, Inc.). Machine-identified *B. braunii* Showa contigs and representative proteins from plants were used as sequence baits. Further gene candidates were identified by searching for appropriate conserved domains and enzyme name keywords using the *B. braunii* Showa web-based annotation tool (http://pathways-pellegrini.mcdb.ucla.edu/botryo1). Contigs and singletons that may overlap and extend contigs of interest were collected by BlastN searches using BlastStation-Free v1.1. Overlapping sequences were assembled into isotigs using Sequencher 5.0 (GeneCodes Corp.) implementing Tablet v1.11 (The James Hutton Institute). Protein models were built by identifying protein-coding regions in “noisy” sequences using FrameDP (http://iant.toulouse.inra.fr/FrameDP/cgi-bin/framedp.cgi) that integrates protein similarities and probabilistic models into a single prediction. Translational start sites were further evaluated using the NetStart 1.0 Server (http://www.cbs.dtu.dk/services/NetStart/). Predicted frameshifts were corrected by scrutinizing the assemblies for the questionable regions using Tablet. Contigs and curated contigs whose deduced protein products showed the highest similarities to fungal, animal or bacterial proteins with no or very low similarities to plant proteins were flagged, and their codon usage frequencies were plotted against those of *B. braunii* (http://www.kazusa.or.jp/codon/index.html) using the Graphical Codon Usage Analyser (http://gcua.schoedl.de/). Flagged contigs and isotigs whose mean differences of codon usage frequencies and relative adaptiveness values significantly exceeded (by 30% or more) those of non-flagged contigs and isotigs of the same pathway were treated as potentially originating from a different organism. Subcellular localization of deduced proteins with protein models covering the N-terminus were predicted using the TargetP 1.1 Server (http://www.cbs.dtu.dk/services/TargetP/).

## Endnote

Dedicated to the memory of Prof. Michael Cusanovich (1942–2010).

## Competing interests

The authors declare no competing interests.

## Authors’ contributions

TPD and TLW cultivated the alga and isolated RNA for sequencing. JHW and JDH performed the assembly. DL, JHW, JDH, and MP assigned automated annotations. DL and MP created the web annotation tool and data depository. IM conceived the study, curated transcripts and reconstructed pathways. IM, DL, TPD and JDH drafted the manuscript. Dedicated to the memory of Prof. Michael Cusanovich (1942–2010). All authors read and approved the final manuscript.

## Supplementary Material

Additional file 1 **Figure S1. –** Denaturing agarose gel analyses of purified *B. braunii* Showa RNA. **Figure S2 –** RT-PCR analysis using *B. braunii* Showa RNA from days 0, 3, and 5. **Figure S3 –** Size distribution of contigs in the assembled *B. braunii* Showa transcriptome. **Table S1** - RNA quantitation from each sample and pooled sample. **Table S2 -** Machine-assembled contigs with the highest sequence coverage in the *B. braunii* Showa transcriptome. **Table S3 -***B. braunii* Showa transcript annotations. **Table S4 -** Top source organisms in the KEGG annotations of the *B. braunii* Showa transcriptome. **Table S6 -** Curated contigs for IPP and DMAPP biosynthesis in the *B. braunii* Showa transcriptome. **Table S7 -** Curated contigs for polyprenyl diphosphate synthases in the *B. braunii* Showa transcriptome. **Table S8 -** Curated contigs for triterpenoid hydrocarbon biosynthesis in the *B. braunii* Showa transcriptome. **Table S9 -** Curated contigs for triterpenoid sterol biosynthesis in the *B. braunii* Showa transcriptome. **Table S10 -** Curated contigs for tetraterpenoid biosynthesis in the *B. braunii* Showa transcriptome. **Table S11 -** Curated contigs for meroterpenoid quinone biosynthesis in the *B. braunii* Showa transcriptome. **Table S12 –** Curated contigs for the biosynthesis of gibberellic acid diterpenes in the *B. braunii* Showa transcriptome. **Table S13 –** Machine-assembled contigs for *S*-adenosylmethionine regeneration in the *B. braunii* Showa transcriptome. **Table S14 –** Machine-assembled contigs for fatty acid biosynthesis, desaturation, elongation and TAG assembly in the *B. braunii* Showa transcriptome. **Table S15 –** Machine-assembled contigs for starch and cellulose biosynthesis in the *B. braunii* Showa transcriptome. **Table S16 –** Machine-assembled contigs for putative ABC transporter pumps in the *B. braunii* Showa transcriptome. **Table S17 –** Machine-assembled contigs related to autophagy in the *B. braunii* Showa transcriptome. Click here for file

Additional file 2 **Table S5 –** Inventory of machine-assembled contigs in the transcriptome of *B. braunii* Showa with KEGG annotations not shared with proteins encoded in the genomes of *C. reinhardtii* (v3.0), *Ch. variabilis* NC64A (v1.0), and *Micromonas* RC299 (v3.0). Click here for file
