# Supplementary material for: Bio-crude transcriptomics: Gene discovery and metabolic network reconstruction for the biosynthesis of the terpenome of the hydrocarbon oil-producing green alga, Botryococcus braunii race B (Showa)*
Source: BMC Genomics. 2012 Oct 30;13:576. doi: 10.1186/1471-2164-13-576 (PMC3533583; doi:10.1186/1471-2164-13-576)
Supplement: Additional file 1 — Figure S1. – Denaturing agarose gel analyses of purified B. braunii Showa RNA. Figure S2 – RT-PCR analysis using B. braunii Showa RNA from days 0, 3, and 5. Figure S3 – Size distribution of contigs in the assembled B. braunii Showa transcriptome. Table S1 - RNA quantitation from each sample and pooled sample. Table S2 - Machine-assembled contigs with the highest sequence coverage in the B. braunii Showa transcriptome. Table S3 -B. braunii Showa transcript annotations. Table S4 - Top source organisms in the KEGG annotations of the B. braunii Showa transcriptome. Table S6 - Curated contigs for IPP and DMAPP biosynthesis in the B. braunii Showa transcriptome. Table S7 - Curated contigs for polyprenyl diphosphate synthases in the B. braunii Showa transcriptome. Table S8 - Curated contigs for triterpenoid hydrocarbon biosynthesis in the B. braunii Showa transcriptome. Table S9 - Curated contigs for triterpenoid sterol biosynthesis in the B. braunii Showa transcriptome. Table S10 - Curated contigs for tetraterpenoid biosynthesis in the B. braunii Showa transcriptome. Table S11 - Curated contigs for meroterpenoid quinone biosynthesis in the B. braunii Showa transcriptome. Table S12 – Curated contigs for the biosynthesis of gibberellic acid diterpenes in the B. braunii Showa transcriptome. Table S13 – Machine-assembled contigs for S-adenosylmethionine regeneration in the B. braunii Showa transcriptome. Table S14 – Machine-assembled contigs for fatty acid biosynthesis, desaturation, elongation and TAG assembly in the B. braunii Showa transcriptome. Table S15 – Machine-assembled contigs for starch and cellulose biosynthesis in the B. braunii Showa transcriptome. Table S16 – Machine-assembled contigs for putative ABC transporter pumps in the B. braunii Showa transcriptome. Table S17 – Machine-assembled contigs related to autophagy in the B. braunii Showa transcriptome. [file 1471-2164-13-576-S1.docx]

# Additional files

**Bio-crude transcriptomics: Gene discovery and metabolic network reconstruction for the biosynthesis of the terpenome of the hydrocarbon oil-producing green alga, *Botryococcus braunii* race B (Showa)**

István Molnár^1,2,§^, David Lopez ^3^, Jennifer H. Wisecaver^4^, Taylor L. Weiss^5^, Timothy P. Devarenne^5^, Matteo Pellegrini^3^, Jeremiah D. Hackett^2,4^

**TABLE OF CONTENT**

**Supplementary Figure 1 –** Denaturing agarose gel analyses of purified *B. braunii* Showa RNA

**Supplementary Figure 2 –** RT-PCR analysis using *B. braunii* Showa RNA from days 0, 3, and 5

**Supplementary Figure 3 –** Size distribution of contigs in the assembled *B. braunii* Showa transcriptome

**Supplementary Table 1** - RNA quantitation from each sample and pooled sample

**Supplementary Table 2 -** Machine-assembled contigs with the highest sequence coverage in the *B. braunii* Showa transcriptome

**Supplementary Table 3 -** *B. braunii* Showa transcript annotations

**Supplementary Table 4 -** Top source organisms in the KEGG annotations of the *B. braunii* Showa transcriptome

**Supplementary Table 5 -** Inventory of machine-assembled contigs in the transcriptome of *B. braunii* Showa with KEGG annotations not shared with proteins encoded in the genomes of *C. reinhardtii* (v3.0), *Ch. variabilis* NC64A (v1.0), and *Micromonas* RC299 (v3.0).

**Supplementary Table 6 -** Curated contigs for IPP and DMAPP biosynthesis in the *B. braunii* Showa transcriptome

**Supplementary Table 7 -** Curated contigs for polyprenyl diphosphate synthases in the *B. braunii* Showa transcriptome

**Supplementary Table 8 -** Curated contigs for triterpenoid hydrocarbon biosynthesis in the *B. braunii* Showa transcriptome

**Supplementary Table 9 -** Curated contigs for triterpenoid sterol biosynthesis in the *B. braunii* Showa transcriptome

**Supplementary Table 10 -** Curated contigs for tetraterpenoid biosynthesis in the *B. braunii* Showa transcriptome

**Supplementary Table 11 -** Curated contigs for meroterpenoid quinone biosynthesis in the *B. braunii* Showa transcriptome

**Supplementary Table 12 –** Curated contigs for the biosynthesis of gibberellic acid diterpenes in the *B. braunii* Showa transcriptome

**Supplementary Table 13 –** Machine-assembled contigs for *S­*-adenosylmethionine regeneration in the *B. braunii* Showa transcriptome

**Supplementary Table 14 –** Machine-assembled contigs for fatty acid biosynthesis, desaturation, elongation and TAG assembly in the *B. braunii* Showa transcriptome

**Supplementary Table 15 –** Machine-assembled contigs for starch and cellulose biosynthesis in the *B. braunii* Showa transcriptome

**Supplementary Table 16 –** Machine-assembled contigs for putative ABC transporter pumps in the *B. braunii* Showa transcriptome

**Supplementary Table 17 –** Machine-assembled contigs related to autophagy in the *B. braunii* Showa transcriptome

## Supplementary Figure 1 – Denaturing agarose gel analyses of purified *B. braunii* Showa RNA

**A.** Analysis of ~5 μg of total RNA isolated from *B. braunii* Showa cultures on days 0, 3, and 5 (see Methods for cultivation conditions). **B.** DNAse treatment of the pooled RNA sample. The pooled RNA sample was treated at 37˚C for 30 min with 1 unit of RNAse-free DNAse per μg of RNA and analyzed on a denaturing agarose gel. **C.** Heat treatment of the pooled RNA sample for analysis of degradation by contaminating nucleases. Two separate 5 μg RNA aliquots were incubated for 1 hr at 0˚C or 37˚C, followed by analysis on a denaturing agarose gel.

## Supplementary Figure 2 – RT-PCR analysis using *B. braunii* Showa RNA from days 0, 3, and 5

Ten micrograms of RNA from each sample were used for first strand cDNA synthesis by standard methods, using an oligo dT primer. Primer sets (see below) for the indicated genes were then used to amplify cDNA fragments from the first strand cDNA of each sample. PCR products were analyzed on a 0.8% agarose DNA gel. The SS and SSL-1 cDNAs cloned into pBSK- were used as positive controls. ^1^Squalene Synthase ^2^Squalene Synthase-Like-1 ^3^Ferredoxin-1 ^4^Ferredoxin NADPH Reductase.

Primer sets:

SS set #1, F: 5’- CCACTGCCAAGCTGATGTACACC, R: 5’- CTGCTCGAGTTAGGCGCTGAGTGTGGGTCTAGG;

SS set #2, F: 5’- CCGCGACTACTTTGAGGACATC, R: 5’- CTGCTCGAGTTAGGCGCTGAGTGTGGGTCTAGG;

SS set #3, F: 5’- CCGCGACTACTTTGAGGACATC, R: 5’- GGTGTACATCAGCTTGGCAGTGG;

SSL-1 set #1, F: 5’- GTTCTGGCCCCAGGAGAT, R: 5’- GGTACTTCCTCGGGTGCC

SSL-1 set #2, F: 5’- ATGACTATGCACCAAGACCACGGAGTC, R: 5’- GGTCATTGTGCAGTTCAACC;

Fdx1 set #1, F: 5’- GCTGCGTCATACCAAGTTACTTTG, R: 5’- TTATGGGTTGAGTGCTTCCTCC;

FNR set #1, F: 5’- GGGGCAATCCTACGGCGTCATTCCCC, R: 5’- TCAGTACACCTCCACGTGCCACTGG;

β-actin set #1, F: 5’- CAAGCCGTACTGTCCTTGTA, R: 5’- GCACAACATTGCCGTACA.

## Supplementary Figure 3 – Size distribution of contigs in the assembled *B. braunii* Showa transcriptome


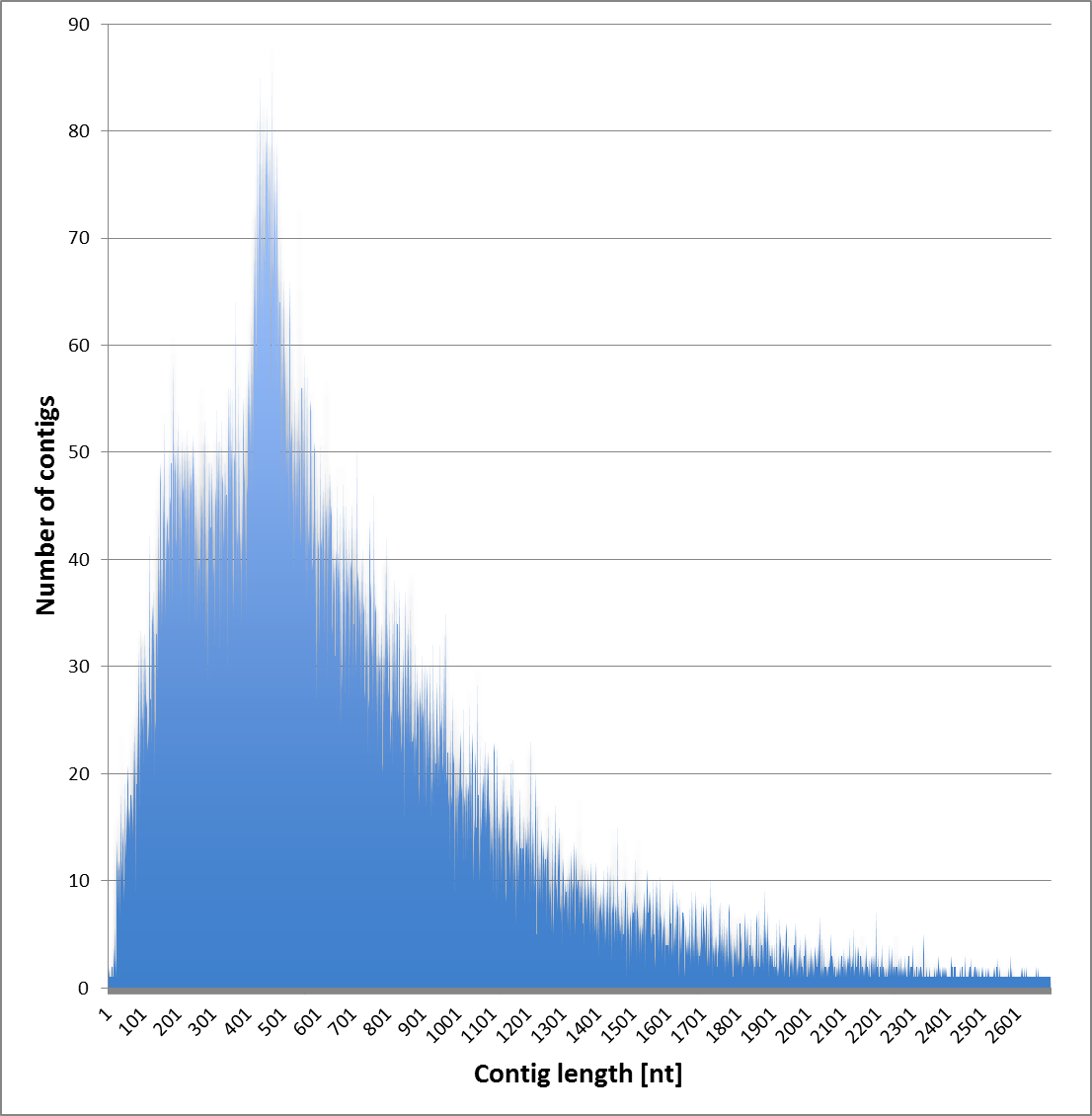


## Supplementary Table 1 - RNA quantitation from each sample and the pooled sample

^1^The amount of pooled RNA is less that the sum of RNA from each sample due to loss of RNA during sample preparation.

## Supplementary Table 2 - Machine-assembled contigs with the highest sequence coverage in the *B. braunii* Showa transcriptome

Coverage indicated in average number of reads at each consensus bases of the contig. Accession numbers refer to the GenBank Transcriptome Shotgun Assembly Sequence Database. Best BlastX hit in GenBank: E-value cutoff at 1e-5. NF, not found.

| **Contig ID**  **Accession** | **Length** | **Average coverage** | **Best BlastX hit in GenBank** | **Hit species** | **Accession** | **E-Value** |
| --- | --- | --- | --- | --- | --- | --- |
| 27783  KA108063 | 435 bp | 8,231.3 | Light-harvesting chlorophyll-a/b binding protein LhcbM9 | *Chlamydomonas incerta* | ABD37914 | 1e-19 |
| 26095  KA106340 | 427 bp | 1,668.8 | NF |  |  |  |
| 30295  KA110778 | 1140 bp | 1,641.5 | Chlorophyll a/b binding protein | *Volvox carteri f. nagariensis* | XP_002948016 | 1e-52 |
| 39882  KA120902 | 584 bp | 1,567.5 | Major light-harvesting chlorophyll a/b protein DsLhcII-2.1 | *Dunaliella salina* | ABD91646 | 3e-54 |
| 8735  KA132443 | 567 bp | 1,275.7 | NF |  |  |  |
| 33302  KA114090 | 2798 bp | 1,222.4 | Major light-harvesting chlorophyll a/b protein 3 | *Dunaliella salina* | ABM74386 | 3e-07 |
| 28452  KA108787 | 611 bp | 1,209.4 | Light-harvesting complex II protein precursor Lhcbm2 | *Acetabularia acetabulum* | DAA05914 | 3e-67 |
| 7832  KA131471 | 526 bp | 1,203.6 | Major light-harvesting chlorophyll a/b protein DsLhcII-2.1 | *Dunaliella salina* | ABD91646 | 2e-72 |
| 23851  KA104377 | 753 bp | 1,203.3 | Photosystem II 13kD protein | *Micromonas pusilla* | XP_003061552 | 2e-06 |
| 35174  KA116115 | 1696 bp | 1,172.5 | Oxygen evolving enhancer protein 3 (PsbQ) | *Chlorella variabilis* | EFN51130 | 2e-42 |
| 29614  KA110052 | 322 bp | 1,166.1 | NF |  |  |  |
| 26056  KA106308 | 517 bp | 1,150.2 | NF |  |  |  |
| 45233  KA126735 | 322 bp | 1,125.4 | NF |  |  |  |
| 7046  KA130624 | 785 bp | 1,106.3 | NF |  |  |  |
| 36519  KA117559 | 357 bp | 1,077.8 | NF |  |  |  |
| 44993  KA126481 | 508 bp | 1,011.1 | Hypothetical protein | *Mycosphaerella graminicola* | EGP85765 | 8e-21 |
| 41203  KA122331 | 755 bp | 989.4 | NF |  |  |  |
| 6256  KA129775 | 615 bp | 953.8 | Hypothetical protein | *Micromonas pusilla* | XP_003064993 | 1e-19 |
| 35760  KA116729 | 727 bp | 948.9 | Chloroplast photosystem I subunit F | *Chlamydomonas incerta* | ABA01144 | 3e-65 |
| 27796  KA108077 | 649 bp | 940.3 | Light-harvesting complex II protein precursor Lhcbm3 | *Acetabularia acetabulum* | DAA05909 | 6e-63 |
| 26222  KA106444 | 593 bp | 930.2 | NF |  |  |  |
| 07092  KA130673 | 433 bp | 920.6 | NF |  |  |  |
| 26084  KA106332 | 297 bp | 917.9 | NF |  |  |  |
| 20081  KA100346 | 1001 bp | 859.8 | Major light-harvesting chlorophyll a/b protein DsLhcII-2.1 | *Dunaliella salina* | ABD91646 | 4e-62 |
| 37234  KA118350 | 493 bp | 824.4 | Hypothetical protein | *Sorghum bicolor* | EES20203 | 2e-24 |
| 40473  KA121532 | 435 bp | 805.5 | NF |  |  |  |
| 7327  KA130930 | 593 bp | 802.7 | Major light-harvesting chlorophyll a/b protein DsLhcII-2.1 | *Dunaliella salina* | ABD91646 | 3e-58 |
| 38649  KA119804 | 465 bp | 792.3 | Unknown | *Glycine max* | ACU24256 | 2e-17 |
| 27164  KA107399 | 397 bp | 791.7 | NF |  |  |  |
| 7015  KA130591 | 537 bp | 774.2 | Chlorophyll a/b binding protein | *Selaginella moellendorffii* | XP_002986911 | 5e-07 |
| 35180  KA116121 | 1324 bp | 773.2 | NF |  |  |  |
| 8374  KA132052 | 814 bp | 765.9 | Major light-harvesting chlorophyll a/b protein DsLhcII-2.1 | *Dunaliella salina* | ABD91646 | 5e-48 |
| 28132  KA108436 | 603 bp | 757.0 | NF |  |  |  |
| 26113  KA106354 | 394 bp | 752.7 | NF |  |  |  |
| 35163  KA116104 | 694 bp | 736.7 | Putative photosystem I subunit V precursor | *Ostreococcus tauri* | XP_003079178 | 4e-25 |
| 35228  KA116170 | 436 bp | 732.3 | NF |  |  |  |
| 35204  KA116146 | 1191 bp | 730.7 | Photosystem I subunit XI, chloroplast precursor | *Micromonas pusilla* | XP_003060103 | 3e-63 |
| 41351  KA122492 | 495 bp | 720.2 | NF |  |  |  |
| 23242  KA103734 | 1931 bp | 715.7 | Photosystem I subunit PsaD | *Glycine max* | ABQ63097 | 6e-59 |
| 08703  KA132409 | 674 bp | 715.3 | Major light-harvesting chlorophyll a/b protein 3 | *Dunaliella salina* | ABM74386 | 1e-36 |
| 41424  KA122571 | 508 bp | 708.8 | NF |  |  |  |
| 24196  KA104750 | 812 bp | 704.6 | Chloroplast glyceraldehyde 3-phosphate dehydrogenase | *Botryococcus braunii* | ACX37507 | 8e-122 |
| 4475  KA126231 | 1333 bp | 699.5 | NF |  |  |  |
| 29047  KA109434 | 552 bp | 693.4 | NF |  |  |  |
| 26172  KA106400 | 785 bp | 685.9 | Light-harvesting complex I chlorophyll-a/b binding protein Lhca5 | *Volvox carteri f. nagariensis* | XP_002949400 | 2e-81 |
| 35183  KA116124 | 1142 bp | 684.8 | Chlorophyll a/b binding protein | *Chlorella variabilis* | EFN58565 | 9e-87 |
| 26142  KA106375 | 594 bp | 682.7 | Major light-harvesting chlorophyll a/b protein DsLhcII-2.1 | *Dunaliella salina* | ABD91646 | 6e-57 |
| 28419  KA108750 | 555 bp | 681.7 | Expressed protein | *Chlorella variabilis* | EFN59113 | 5e-10 |
| 08056  KA131712 | 964 bp | 661.5 | Putative senescence-associated protein | *Cupressus sempervirens* | ACA30301 | 8e-32 |

## Supplementary Table 3 - *B. braunii* Showa transcript annotations

| **Database** | **Number of contigs** |
| --- | --- |
| KEGG Pathways | 6666 |
| Panther Pathways | 1736 |
| MetaCyc Pathways | 1932 |
| Reactome Pathways | 3423 |
| Gene Ontology (inferred from *C. reinhardtii*) | 2008 |
| Gene Ontology (inferred from *A. thaliana*) | 4292 |
| MapMan Ontology (inferred from *C. reinhardtii*) | 3999 |
| MapMan Ontology (inferred from *A. thaliana*) | 3874 |
| Pfam Domains | 19900 |

## Supplementary Table 4 - Top source organisms in the KEGG annotations of the *B. braunii* Showa transcriptome

| **Species** | **Number of contigs** |
| --- | --- |
| *Volvox carteri* | 886 |
| *Chlamydomonas reinhardtii* | 762 |
| *Physcomitrella patens* ssp. patens | 430 |
| *Fusarium graminearum* | 207 |
| *Arabidopsis thaliana* | 192 |
| *Vitis vinifera* | 187 |
| *Oryza sativa* | 156 |
| *Magnaporthe oryzae* | 142 |
| *Ostreococcus lucimarinus* | 127 |
| *Populus trichocarpa* | 126 |
| *Ostreococcus tauri* | 119 |
| *Ricinus communis* | 119 |
| *Sorghum bicolor* | 114 |
| *Neurospora crassa* | 111 |
| *Podospora anserina* | 108 |
| Other | 2692 |

## Supplementary Table 5 - Inventory of machine-assembled contigs in the transcriptome of *B. braunii* Showa with KEGG annotations not shared with proteins encoded in the genomes of *C. reinhardtii* (v3.0), *Ch. variabilis* NC64A (v1.0), and *Micromonas* RC299 (v3.0).

Included as a separate Excel spreadsheat.

## Supplementary Table 6 - Curated contigs for IPP and DMAPP biosynthesis in the *B. braunii* Showa transcriptome

Coverage indicated in reads per thousand bases of the curated contig. Protein models may be truncated at either or both ends. Localization as predicted by TargetP: C, chloroplast; M: mitochondrion; S: secretory pathway; NP: no prediction. Best BlastP hit with cutoff at 1e-5. GenBank: excluding *Ch. variabilis* and *C. reinhardtii*, shown separately. *, curated contig that may have originated from a transcript of a *B. braunii* cohabitant organism. NF, not found.

| **Enzyme (symbol)** | **Contig ID,**  **Accession** | **Contig length, coverage** | **Protein length,**  **Localization** | **Best BlastP hit in** | | | | |
| --- | --- | --- | --- | --- | --- | --- | --- | --- |
|  |  |  |  | **GenBank** | ***Chlorella variabilis*** | | ***Chlamydomonas reinhardtii*** | |
| **Mevalonate (MVA) pathway** | | | | | | | | |
| Acetyl-CoA acetyltransferase (AtoB), E.C. 2.3.1.9 | 27950  KA659957 | 1,100 bp 2.7/kb | 249 aa | ACN40771, *Picea sitchensis*  (1e-87, 56%) | EFN52163  (4e-83, 54%) | | EDP02040  (7e-82, 56%) | |
|  | 01172*  KA091417 | 609 bp 23.0/kb | 176 aa | EFQ27722, *Glomerella graminicola*  (5e-105, 85%) | EFN52163  (7e-47, 48%) | | EDP02040  (1e-43, 52%) | |
|  | 43048*  KA659958 | 943 bp 322.3/kb | 86 aa | AEO70500, *Thielavia terrestris*  (2e-47, 93%) | EFN52163  (3e-35, 64%) | | EDP02040  (4e-24, 61%) | |
| Hydroxymethylglutaryl-CoA synthase (HMGS),  E.C. 2.3.3.10 | 37312  KA118436 | 1,363 bp 2.9/kb | 26 aa | CBI15535, *Vitis vinifera*  (3e-10, 79%) | EFN59211  (4e-11, 75%) | | NF | |
|  | 00453*  KA659959 | 308 bp 16.3/kb | 80 aa | EAQ85120, *Chaetomium globosum* (5e-37, 78%) | EFN59211  (2e-8, 36%) | | NF | |
| Hydroxymethylglutaryl-CoA reductase (HMGR),  E.C. 1.1.1.34 | 34020*  KA659960 | 529 bp 3.8/kb | 173 aa | EDR01917, *Laccaria bicolor*  (1e-99, 89%) | NF | | NF | |
| Mevalonate kinase (MVK),  E.C. 2.7.1.36 | NF |  |  |  |  | |  | |
| 5-phosphomevalonate kinase (PMK), E.C. 4.1.1.33 | NF |  |  |  |  | |  | |
| Mevalonate 5-diphosphate decarboxylase (MVD),  E.C. 4.1.1.33 | NF |  |  |  |  | |  | |
| **Biosynthesis of precursors for the MEP/DOXP pathway** | | | | | | | | |
| Phosphoglycerate kinase (PGK), E.C. 2.7.2.3 | 19595  KA659920 | 1,492 bp 32.2/kb | 399 aa  O | AAD55564, *Volvox carteri f. nagariensis* (0.0, 69%)  FX085563, *Botryococcus braunii BOT-22* (64 aa, 1e-29, 89%) | | EFN59289 (0.0, 69%) | | EDO98586 (0.0, 69%) |
|  | 23866  KA659919 | 2,424 bp 887.0/kb | 405 aa  O | AAD55564, *Volvox carteri f. nagariensis* (0.0, 80%) | | EFN59289 (0.0, 78%) | | EDO98586 (0.0, 80%) |
|  | 38440*  KA659921 | 416 bp  21.6/kb | 120 aa | EEY15333, *Verticillium albo-atrum*  (2e-67, 90%) | | EFN59488  (1e-36, 59%) | | EDO98586 (7e-38, 54%) |
| Glyceraldehyde phosphate dehydrogenase, NAD^+^ (GAPDH), E.C. 1.2.1.12 (Glycolysis & gluconeogenesis) | 24196  KA659922 | 1,865 bp 1540.0/kb | 411 aa  M | ACX37507, *Botryococcus braunii*  (0.0, 99%)  FX085420, *Botryococcus braunii* BOT-22 (67 aa, 1e-38, 99%) | | EFN51002  (1e-165, 74%) | | EDP09609 (0.0, 84%) |
|  | 32644  KA659924 | 2,047 bp 532.0/kb | 338 aa  M | ABN51378, *Clostridium thermocellum* (0.0, 75%)  FX085421, *Botryococcus braunii* BOT-22 (157 aa, 1e-92, 96%)  FX085422, *Botryococcus braunii* BOT-22 (121 aa, 1e-67, 94%) | | EFN53819 (0.0, 74%) | | AAA86856 (5e-175, 70%) |
|  | 32244*  KA112928 | 735 bp 89.8/kb | 103 aa | EGE07162, *Trichophyton tonsurans*  (1e-47, 71%) | | EFN53819  (3e-36, 55%) | | AAA86856 (6e-37, 59%) |
|  | 34773*  KA659925 | 891 bp 382.7/kb | 233 aa | EFQ30042, *Glomerella graminicola*  (2e-115, 91%) | | EFN53819  (2e-118, 72%) | | EDO96576 (1e-115, 69%) |
|  | 37702*  KA659923 | 652 bp 9.2/kb | 190 aa  O | ABT17340, *Lactarius rubrilacteus*  (2e-119, 87%) | | EFN53819  (2e-103, 76%) | | EDO96576 (1e-95, 71%) |
| Glyceraldehyde phosphate dehydrogenase, NADP^+^ (GAPDH), E.C. 1.2.1.13 (Benson-Calvin cycle) | 15369  KA659926 | 915 bp 13.1/kb | 262 aa | CAA59681, *Schizosaccharomyces pombe* (4e-135, 75%) | | EFN53819  (1e-143, 79%) | | AAA86856 (1e-128, 73%) |
| NADP+-dependent glyceraldehyde 3-phosphate dehydrogenase (GAPN),  E.C. 1.2.1.9 | 30401  KA659927 | 3,158 bp 67.8/kb | 495 aa | AAD03388, *Arabidopsis thaliana*  (0.0, 74%) | | EFN50637 (0.0, 71%) | | EDP03116 (0.0, 70%) |
| Phosphoglycerate mutase (PGAM), E.C. 5.4.2.1 | 09999  KA133805 | 3,061 bp 49.0/kb | 554 aa | EFJ52833, *Volvox carteri f. nagariensis* (0.0, 68%) | | EFN53796 (0.0, 72%) | | EDP08189 (0.0, 70%) |
|  | 20818  KA659930 | 332 bp 27.1/kb | 76 aa | EDQ58096, *Physcomitrella patens* ssp*.* patens (2e-24, 64%) | | EFN53685  (4e-20, 61%) | | EDP08189 (1e-18, 58%) |
|  | 24169  KA659931 | 766 bp 11.7/kb | 254 aa | ACO62635, *Micromonas sp*. RCC299  (2e-108, 64%) | | EFN53796  (5e-117, 61%) | | EDP08189  (7e-107, 61%) |
|  | 02501*  KA659928 | 365 bp 8.2/kb | 69 aa | EFQ29477, *Glomerella graminicola*  (2e-29, 84%) | | NF | | NF |
|  | 20593*  KA659929 | 545 bp 3.7/kb | 178 aa | EFI92345, *Schizophyllum commune*  (6e-101, 81%) | | EFN53796  (8e-24, 39%) | | EDP08188  (3e-23, 38%) |
|  | 38683*  KA659932 | 342 bp 8.8/kb | 77 aa | EGY17173, *Verticillium dahliae*  (8e-18, 66%) | | NF | | NF |
| Phosphopyruvate hydratase (ENO), E.C. 4.2.1.11 | 31377  KA659935 | 2,223 bp 78.3/kb | 453 aa | EFJ51080, *Volvox carteri f. nagariensis* (0.0, 68%) | | EFN53429  (1e-52, 63%) | | EDO96709  (0.0, 68%) |
|  | 43373  KA659933 | 2,471 bp 511.5/kb | 478 aa  O | EFJ51080, *Volvox carteri f. nagariensis* (0.0, 77%)  FX085138, *Botryococcus braunii* BOT-22 (82 aa, 2e-48, 100%) | | EFN53429  (6e-68, 59%) | | EDO96709  (0.0, 77%) |
|  | 23224*  KA659934 | 886 bp 103.8/kb | 236 aa | EGR50787, *Trichoderma reesei*  (6e-153, 89%) | | EFN53429  (2e-22, 72%) | | EDO96709  (8e-102, 63%) |
| Pyruvate kinase (PK),  E.C. 2.7.1.40 | 10234  KA659936 | 2,637 bp 30.3/kb | 508 aa  O | ADD52598, *Dunaliella salina*  (0.0, 80%) | | EFN57010  (6e-83, 34%) | | EDP03577  (0.0, 81%) |
|  | 10955  KA090586 | 3,678 bp 64.4/kb | 489 aa | ACO64546, *Micromonas sp*.  (1e-180, 59%)  FX085139, *Botryococcus braunii* BOT-22 (106 aa, 7e-61, 94%) | | EFN57958  (9e-69, 32%) | | EDP01525  (0.0, 58%) |
|  | 16949  KA659937 | 1,083 bp 12/kb | 274 aa | EFJ40446, *Volvox carteri f. nagarensis* (4e-114, 69%) | | EFN57958  (2e-42, 36%) | | EDO97877  (4e-121, 65%) |
|  | 23205  KA659938 | 2,039 bp 5.4/kb | 394 aa | EEH56903, *Micromonas pusilla*  (4e-108, 50%) | | EFN57958  (5e-25, 44%) | | EDP01525  (8e-112, 50%) |
|  | 41366  KA659939 | 1,104 bp 1.8/kb | 205 aa | ACO64546, *Micromonas sp*.  (1e-47, 48%) | | EFN57958  (9e-16, 31%) | | EDP01525  (2e-40, 46%) |
|  | 13636  KA659940 | 2,879 bp 38.6/kb | 557 aa  M | EEF49016, *Ricinus communis*  (0.0, 58%) | | EFN57958 (0.0, 61%) | | EDP03577  (1e-84, 33%) |
|  | 22781  KA659941 | 1,267 bp 2.4/kb | 285 aa | EFJ40338, *Volvox carteri f. nagariensis* (3e-102, 61%) | | EFN57010  (1e-63, 46%) | | EDP01697  (2e-110, 59%) |
|  | 41736  KA659943 | 2,254 bp 26.6/kb | 521 aa  O | EDQ54483, *Physcomitrella patens ssp. patens* (0.0, 61%)  FX085140, *Botryococcus braunii* BOT-22 (56 aa, 7e-29, 94%) | | EFN58575 (0.0, 63%) | | EDP01525  (2e-72, 31%) |
|  | 44263  KA659944 | 1,298 bp 4.6/kb | 367 aa | EFJ40338, *Volvox carteri f. nagariensis* (3e-124, 53%) | | EFN57010  (2e-118, 50%) | | EDP01697  (7e-131, 53%) |
|  | 25894*  KA659942 | 485 bp 6.2/kb | 68 aa | EFQ31964, *Glomerella graminicola*  (1e-36, 93%) | | EFN53164  (5e-07, 36%) | | EDP03577  (6e-11, 38%) |

| **MEP/DOXP pathway** | | | | | | |
| --- | --- | --- | --- | --- | --- | --- |
| 1-deoxy-D-xylulose 5-phosphate synthase (DXS), E.C. 2.2.1.7 | 07667  DXS-III  KA659945 | 1,243 bp 4.0/kb | 241 aa | ACT21080, *Dunaliella salina*  (7e-100, 66%)  JF284352, *Botryococcus braunii* Showa (730 aa, 2e-128, 90%) | EFN51764  (3e-105, 65%) | EDO97255  (1e-112, 68%) |
|  | 11032  DXS-III  KA659946 | 2,280 bp 29.4/kb | 389 aa | EFJ41925, *Volvox carteri f. nagariensis* (4e-17, 63%)  FX085274, *Botryococcus braunii* BOT-22 (107 aa, 4e-53, 84%)  FX085275, *Botryococcus braunii* BOT-22 (60 aa, 4e-26, 81%)  JF284352, *Botryococcus braunii* Showa (730 aa, 0.0, 100%) | EFN51764  (0.0, 68%) | EDO97255  (2e-177, 61%) |
|  | 10163  DXS-I  KA659947 | 3,361 bp 66.6/kb | 770 aa  C | EFJ41925, *Volvox carteri f. nagariensis* (0.0, 60%)  JF284350, *Botryococcus braunii* Showa (770 aa, 0.0, 99%) | EFN51764  (0.0, 61%) | CAA07554  (0.0, 61%) |
|  | 42027  DXS-II  KA659948 | 2,774 bp 93.7/kb | 771 aa  C | ACT21080, *Dunaliella salina*  (0.0, 62%)  FX085276, *Botryococcus braunii* BOT-22 (81 aa, 7e-34, 70%)  FX085277, *Botryococcus braunii* BOT-22 (70 aa, 1e-27, 72%)  JF284351, *Botryococcus braunii* Showa (771aa, 0.0, 100%) | EFN51764  (0.0, 62%) | EDO97255  (0.0, 63%) |
| 1-deoxy-D-xylulose-5-phosphate reductoisomerase (DXR), E.C. 1.1.1.267 | 41877  KA123067 | 2,087 bp 90.1/kb | 474 aa  C | EFJ45264, *Volvox carteri f. nagariensis* (0.0, 71%) | EFN58715  (0.0, 74%) | EDP02894  (0.0, 62%) |
| 2-*C*-methyl-D-erythritol 4-phosphate cytidylyltransferase (IspD), E.C. 2.7.7.60 | 15261  KA659949 | 1,302 bp 47.6/kb | 298 aa  C | EFJ40752, *Volvox carteri f. nagariensis* (7e-114, 74%) | EFN57889  (6e-29, 77%) | EDO99224  (4e-124, 68%) |
| 4-(cytidine 5’-diphospho)-2-*C*-methyl-D-erythritol kinase (IspE), E.C. 2.7.1.148 | 15091  KA659950 | 2,077 bp 72.7/kb | 314 aa | ACO67590, *Micromonas sp*.  (2e-124, 67%) | EFN59579  (1e-146, 72%) | EDP02028  (2e-116, 65%) |
| 2-*C*-methyl-D-erythritol 2,4-cyclodiphosphate synthase (IspF), E.C. 4.6.1.12 | 32528  KA659951 | 2,003 bp 200.7/kb | 239 aa  C | EFJ50974, *Volvox carteri f. nagariensis* (2e-90, 79%) | EFN54075  (3e-90, 76%) | EDP05431  (2e-95, 80%) |
| (*E*)-4-hydroxy-3-methylbut-2-enyl diphosphate synthase (IspG), E.C. 1.17.7.1 | 30410  KA659952 | 3,607 bp 242.9/kb | 737 aa  C | EFJ45455, *Volvox carteri f. nagariensis* (0.0, 74%)  FX085293, *Botryococcus braunii* BOT-22 (60 aa, 7e-35, 100%) | EFN56290  (0.0, 67%) | EDP05383  (0.0, 72%) |
| 4-hydroxy-3-methylbut-2-enyl diphosphate reductase (IspH), E.C. 1.17.1.2 | 32418  KA659953 | 2,595 bp  426.6/kb | 502 aa  C/M | EFJ51887, *Volvox carteri f. nagariensis* (0.0, 63%) | EFN56438  (0.0, 65%) | EDO97597  (0.0, 62%) |
| Isopentenyl-diphosphate Δ-isomerase (Idi), E.C. 5.3.3.2 | 08862  KA659954 | 258 bp 11.6/kb | 71 aa | ABB80114, *Haematococcus pluvialis*  (5e-19, 59%) | EFN53784  (2e-23, 58%) | EDO97534  (1e-12, 44%) |
|  | 13533  KA659956 | 1,547 bp 17.5/kb | 124 aa | EFJ46000, *Volvox carteri f. nagariensis* (1e-12, 51%) | NF | EDP02662  (5e-19, 35%) |
|  | 34876*  KA659955 | 714 bp 40.6/kb | 77 aa | EFY98343, *Metarhizium anisopliae*  (2e-34, 77%) | EFN53784  (9e-11, 33%) | EDO97534  (5e-12, 39%) |
| Isopentenyl-diphosphate Δ-isomerase (Idi-2), E.C. 5.3.3.2 | NF |  |  |  |  |  |
| **Production of D-xylulose 5-phosphate in the pentose phosphate cycle** | | | | | | |
| Ribulose phosphate 3-epimerase (RPE), E.C. 5.1.3.1 | 30447  KA659961 | 2,096 bp  55.8/kb | 278 aa  C | AAD09954, *Arabidopsis thaliana*  (1e-133, 76%) | EFN57968 (2e-127, 75%) | EDP05517  (2e-137, 72%) |
|  | 24626*  KA105133 | 325 bp  36.9/kb | 75 aa | EEY18335, *Vericillium arbo-atrum*  (1e-38, 88%) | EFN59756  (8e-25, 68%) | EDP07568  (5e-25, 65%) |
| Transketolase (TKTL),  E.C. 2.2.1.1 | 32329  KA659962 | 2,998 bp  436.6/kb | 740 aa | EFJ45315, *Volvox carteri f. nagariensis* (0.0, 69%)  FX085315, *Botryococcus braunii* BOT-22 (78 aa, 8e-40, 87%)  FX085314, *Botryococcus braunii* BOT-22 (51 aa, 5e-25, 90%) | EFN54980  (0.0, 74%) | EDP06856  (0.0, 73%) |
|  | 23409*  KA103911 | 757 bp  67.4/kb | 130 aa | EEY14530, *Verticillium albo-atrum*  (3e-81, 89%) | EFN54980  1e-22, 45% | EDP06856  3e-19, 38% |
| Phosphoketolase (XFP),  E.C. 4.1.2.9 | 43065*  KA124382 | 464 bp  38.8/kb | 74 aa | EEY21400, *Verticillium albo-atrum*  (3e-31, 79%) | NF | NF |

## Supplementary Table 7 - Curated contigs for polyprenyl diphosphate synthases in the *B. braunii* Showa transcriptome

Coverage indicated in reads per thousand bases of the curated contig. Protein models may be truncated at either or both ends. Localization as predicted by TargetP: C, chloroplast; M: mitochondrion; S: secretory pathway; NP: no prediction. Best BlastP hit with cutoff at 1e-5. GenBank: excluding *Ch. variabilis* and *C. reinhardtii*, shown separately. *, curated contig that may have originated from a transcript of a *B. braunii* cohabitant organism. NF, not found.

| **Enzyme (symbol)** | **Contig ID**  **Accession** | **Contig length, coverage** | **Protein length, Localization** | **Best BlastP hit in** | | |
| --- | --- | --- | --- | --- | --- | --- |
|  |  |  |  | **GenBank** | ***Chlorella variabilis*** | ***Chlamydomonas reinhardtii*** |
| Geranyl diphosphate synthase (GDPS), E.C. 2.5.1.1 | 21328  KA659965 | 1,523 bp  23.0/kb | 349 aa | EFJ50585, *Volvox carteri f. nagariensis* (8e-132, 58%) | EFN53887  (8e-126, 58%) | EDP05515  (1e-142, 60%) |
| Farnesyl diphosphate synthase (FDPS),  E.C. 2.5.1.10 | 13377  KA659964 | 3,937 bp  78.7/kb | 337 aa | EFJ41223, *Volvox carteri f. nagariensis* (2e-137, 56%) | EFN59689  (5e-160, 63%) | EDP03194  (3e-153, 60%) |
|  | 15137  KA659963 | 1,823 bp  34.0/kb | 362 aa  O | EFJ41223, *Volvox carteri f. nagariensis* (7e-140, 58%) | EFN59689  (1e-170, 65%) | EDP03194  (6e-158, 62%) |
|  | 09221*  KA132966 | 827 bp  2.4/kb | 223 aa | BAD15361, *Lactarius chrysorrheus* (8e-57, 48%) | EFN59689  (1e-29, 47%) | EDP03194  (7e-33, 38%) |
| Geranlylgeranyl diphosphate synthase (GGDPS),  E.C. 2.5.1.29 | 31128  KA659966 | 2,403 bp  67.8/kb | 359 aa  M | EFJ45441, *Volvox carteri f. nagariensis* (1e-84, 49%) | EFN53881  (8e-49, 45%) | EDO96545  (1e-85, 48%) |
| Solanesyl diphosphate synthase (SDPS), E.C. 2.5.1.84 | 10708  KA659967 | 1,170 bp  19.7/kb | 188 aa | EFJ51461, *Volvox carteri f. nagariensis* (3e-49, 46%) | EFN51886  (6e-27, 35%) | EDP08684  (5e-52, 45%) |
| Decaprenyl diphosphate synthase (PDSS1),  E.C. 2.5.1.91 | 34796*  KA115728 | 397 bp  15.1/kb | 34 aa | EEY17180, *Verticillium albo-atrum* (8e-9, 79%) | NF | NF |
| Dehydrodolichyl diphosphate synthase (DHDDS),  E.C. 2.5.1.- | 03498  KA659968 | 576 bp  6.9/kb | 84 aa | ACO67861, *Micromonas sp*.  (5e-16, 53%) | EFN60106  (8e-10, 42%) | EDO98487  (3e-11, 45%) |
|  | 12780  KA659969 | 2,387 bp  14.7/kb | 167 aa | AEO54154, *Myceliophthora thermophile* (9e-9, 27%) | EFN53865  (5e-12, 41%) | EDP04887  (3e-6, 57%) |
|  | 33019  KA113778 | 1,229 bp  11.4/kb | 237 aa | ACO67861, *Micromonas sp*.  (1e-70, 52%) | EFN60106  (5e-61, 47%) | EDO98487  (4e-61, 48%) |
|  | 24316*  KA659970 | 403 bp  22.3/kb | 42 aa | EEY19826, *Verticillium albo-atrum* (2e-19, 83%) | EFN53865  (4e-6, 31%) | NF |

## Supplementary Table 8 - Curated contigs for triterpenoid hydrocarbon biosynthesis in the *B. braunii* Showa transcriptome

Coverage indicated in reads per thousand bases of the curated contig. Protein models may be truncated at either or both ends. Localization as predicted by TargetP: C, chloroplast; M: mitochondrion; S: secretory pathway; NP: no prediction. Best BlastP hit with cutoff at 1e-5. GenBank: excluding *Ch. variabilis* and *C. reinhardtii*, shown separately. *, curated contig that may have originated from a transcript of a *B. braunii* cohabitant organism. NF, not found.

| **Enzyme (symbol)** | **Contig ID Accession** | **Contig length, coverage** | **Protein length,**  **Localization** | **Best BlastP hit in** | | |
| --- | --- | --- | --- | --- | --- | --- |
|  |  |  |  | **GenBank** | ***Chlorella variabilis*** | ***Chlamydomonas reinhardtii*** |
| Squalene synthase (SQS),  E.C. 2.5.1.21 | 13418  BSS  KA659971 | 3,074 bp  136.3/kb | 461 aa  O | AAF20201, *Botryococcus braunii* Showa (0.0, 100%) | EFN59888  (5e-69, 59%) | EDP06129  (1e-149, 57%) |
|  | 11016  SSL-1  KA659972 | 2,729 bp  67.8/kb | 403 aa  O | AEL16715, *Botryococcus braunii* Showa (0.0, 100%) | EFN59888  (5e-51, 44%) | EDP06129  (6e-105, 43%) |
|  | 16777  SSL-2  KA659973 | 2,612 bp  35.2/kb | 272 aa | AEL16716, *Botryococcus braunii* Showa (0.0, 100%) | EFN50644  (4e-20, 67%) | EDP06129  (2e-66, 54%) |
|  | 11158  SSL-3  KA659974 | 2,389 bp  52.7/kb | 383 aa  O | AEL16717, *Botryococcus braunii* Showa (0.0, 100%) | EFN59888  (4e-53, 46%) | EDP06129  (8e-114, 45%) |
| Isoprenoid biosynthesis-related protein, Class 1 (ISR) | 15288  KA659975 | 2,273 bp  12.3/kb | 321 aa  M | EFJ41732, *Volvox carteri f. nagariensis* (6e-85, 48%) | EFN52015  (4e-59, 38%) | EDP03291  (7e-55, 46%) |
| Sterol 24-*C*-methyltransferase (SMT), E.C. 2.1.1.41 | 44999  TMT-1  KA659987 | 2,972 bp  270.2/kb | 378 aa  S | EFJ51011, *Volvox carteri f. nagariensis* (2e-122, 51%)  JN828962, *Botryococcus braunii* Showa (0.0, 99%) | EFN52937  (9e-17, 30%) | EDP05221  (5e-125, 50%) |
|  | 15912  TMT-2  KA659985 | 1,437 bp  71.7/kb | 378 aa  S | EFJ51011, *Volvox carteri f. nagariensis* (3e-121, 50%)  JN828963, *Botryococcus braunii* Showa (0.0, 100%) | EFN52937  (2e-15, 29%) | EDP05221  (1e-124, 50%) |
|  | 35334  TMT-3  KA659981 | 1,749 bp  768.4/kb | 379 aa  S | EFJ51011, *Volvox carteri f. nagariensis* (6e-123, 51%)  JN828964, *Botryococcus braunii* Showa (0.0, 100%) | EFN52937  (7e-14, 27%) | EDP05221  (3e-128, 50%) |
|  | 35335  SMT-1  KA659986 | 3,091 bp  1196.7/kb | 389 aa  NP | EFJ51011, *Volvox carteri f. nagariensis* (3e-106, 44%)  JN828965, *Botryococcus braunii* Showa (0.0, 100%) | EFN52937  (3e-15, 29%) | EDP05221  (1e-107, 42%) |
|  | 15560  SMT-2  KA659984 | 1,839 bp  203.4/kb | 389 aa  O | EFJ51011, *Volvox carteri f. nagariensis* (4e-118, 49%)  JN828966, *Botryococcus braunii* Showa (0.0, 100%) | EFN52937  (3e-15, 28%) | EDP05221  (3e-120, 51%) |
|  | 14599  SMT-3  KA659983 | 1,761 bp  173.8/kb | 391 aa  O | EFJ51011, *Volvox carteri f. nagariensis* (4e-92, 42%)  JN828967, *Botryococcus braunii* Showa (0.0, 99%) | EFN52937  (4e-14, 29%) | EDP05221  (2e-91, 43%) |
|  | 02780  KA659982 | 683 bp  11.7/kb | 215 aa | EFJ51011, *Volvox carteri f. nagariensis* (4e-80, 68%) | NF | EDP05221  (6e-86, 71%) |
|  | 41995  KA123198 | 1,065 bp  15.0/kb | 154 aa | EFJ51011, *Volvox carteri f. nagariensis* (2e-32, 47%) | NF | EDP05221  (5e-35, 43%) |
|  | 45698  KA659988 | 873 bp  5.7/kb | 187 aa  O | EFJ51011, *Volvox carteri f. nagariensis* (4e-50, 48%) | EFN52196  (2e-7, 36%) | EDP05221  (6e-56, 49%) |
|  | 32241*  KA112925 | 393 bp  15.3/kb | 36 aa | EGR49758, *Trichoderma reesei*  (7e-13, 86%) | NF | EDP05221  (5e-5, 43%) |
| 24-methylenesterol *C*-methyltransferase (MSMT),  E.C. 2.1.1.143 | 31094  KA659989 | 1,958 bp  11.2/kb | 219 aa | EFJ51011, *Volvox carteri f. nagariensis* (8e-31, 32%) | NF | EDP05221  (1e-33, 32%) |
| Squalene monooxygenase (SQLE), E.C. 1.14.13.132 | 03198  KA659976 | 1,188 bp  5.9/kb | 128 aa | EFJ48412, *Volvox carteri f. nagariensis* (2e-21, 40%) | EFN52926  (2e-24, 37%) | NF |
|  | 08818  KA132533 | 596 bp  3.4/kb | 77 aa | ABO97482, *Ostreococcus lucimarinus* (5e-20, 51%) | EFN52926  (1e-23, 52%) | NF |
|  | 22878  KA659977 | 3,125 bp  20.2/kb | 483 aa  O | CBI25076, *Vitis vinifera*  (3e-97, 38%) | EFN52926  (1e-91, 38%) | EDO99128  (1e-6, 30%) |
|  | 27993  KA659978 | 1,055 bp  9.5/kb | 126 aa | EFJ48412, *Volvox carteri f. nagariensis* (6e-20, 40%) | EFN52926  (6e-26, 43%) | NF |
|  | 33460  KA659979 | 516 bp  3.9/kb | 115 aa | EFJ48412, *Volvox carteri f. nagariensis* (2e-10, 37%) | EFN52926  (3e-13, 42%) | NF |
|  | 40155  KA659980 | 405 bp  9.9/kb | 120 aa | ABK24903, *Picea sitchensis*  (1e-43, 60%) | EFN52926  (1e-43, 55%) | NF |
|  | 43810  KA125206 | 474 bp  6.3/kb | 92 aa | BAF79915, *Euphorbia tirucalli*  (3e-8, 34%) | EFN52926  (2e-10, 34%) | NF |

## Supplementary Table 9 - Curated contigs for triterpenoid sterol biosynthesis in the *B. braunii* Showa transcriptome

Coverage indicated in reads per thousand bases of the curated contig. Protein models may be truncated at either or both ends. Localization as predicted by TargetP: C, chloroplast; M: mitochondrion; S: secretory pathway; NP: no prediction. Best BlastP hit with cutoff at 1e-5. GenBank: excluding *Ch. variabilis* and *C. reinhardtii*, shown separately. *, curated contig that may have originated from a transcript of a *B. braunii* cohabitant organism. NF, not found, NA: GenBank accession numbers not available for transcripts shorter than 200 bp.

| **Enzyme (symbol)** | **Contig ID, Accession** | **Contig length, coverage** | **Protein length,**  **Localization** | **Best BlastP hit in** | | |
| --- | --- | --- | --- | --- | --- | --- |
|  |  |  |  | **GenBank** | ***Chlorella variabilis*** | ***Chlamydomonas reinhardtii*** |
| **Phytosterol biosynthesis** | | | | | | |
| Cycloartenol synthase (CAS), E.C. 5.4.99.8 | 24069  KA659990 | 3,176 bp  47.9/kb | 742 aa | EFH60015, *Arabidopsis lyrata ssp. lyrata* (0.0, 57%) | EFN56189  (0.0, 61%) | EDP09612  (0.0, 57%) |
| Methylsterol monooxygenase (ERG25), E.C. 1.14.13.72 | 20688  KA659991 | 667 bp  3.0/kb | 213 aa | EFJ53159, *Volvox carteri f. nagariensis* (4e-84, 55%) | EFN55855  (2e-94, 61%) | EDP08564  (6e-89, 56%) |
|  | 01254*  NA | 162 bp  12.3/kb | 53 aa | EGR51743, *Trichoderma reesei*  (5e-30, 96%) | NF | NF |
|  | 44114*  KA659992 | 1,134 bp  3.5/kb | 132 aa | EGO23019, *Serpula lacrymans* var. lacrymans (2e-62, 73%) | NF | NF |
| Cycloeucalenol cycloisomerase (CPI1),  E.C. 5.5.1.9 | 32293  KA659993 | 1,246 bp  12.8/kb | 277 aa  M | EFJ44460, *Volvox carteri f. nagariensis* (2e-113, 62%) | EFN54514  (7e-128, 67%) | EDO99790  (1e-124, 63%) |
| Sterol 14-demethylase (CYP51), E.C. 1.14.13.70 | 12030  KA659994 | 2,443 bp  29.5/kb | 498 aa  S | EFJ52296, *Volvox carteri f. nagariensis* (0.0, 59%) | EFN54386  (0.0, 60%) | EDP06763  (0.0, 61%) |
| Δ-14-sterol reductase (ERG24), E.C. 1.3.1.70 | 11517  KA659995 | 1,763 bp  9.1/kb | 235 aa | ABO95305, *Ostreococcus lucimarinus* (2e-87, 57%) | NF | EDP06826  (2e-88, 54%) |
|  | 18937*  KA659996 | 217 bp  9.2/kb | 64 aa | EEY16446, *Verticillium albo-atrum*  (6e-24, 69%) | NF | EDP06826  (6e-6, 37%) |
| Cholestenol Δ-isomerase (EBP), E.C. 5.3.3.5 | 17464  KA659997 | 1,602 bp  10.0/kb | 226 aa  O | ADG02944, *Gossypium hirsutum*  (1e-53, 39%) | EFN58092  (1e-73, 47%) | EDO96892  (2e-69, 48%) |
| Lathosterol oxidase (SC5DL),  E.C. 1.14.21.6 | 03862  KA659998 | 1,408 bp  12.1/kb | 324 aa  O | EFJ48046, *Volvox carteri f. nagariensis* (4e-113, 59%) | EFN51518  (3e-94, 56%) | EDO97454  (8e-119, 61%) |
| 7-dehydrocholesterol reductase (DHCR7),  E.C. 1.3.1.21 | 11475  KA659999 | 2,237 bp  13.4/kb | 450 aa  O | BAJ86018, *Hordeum vulgare ssp. vulgare* (2e-173, 57%) | NF | EDP06826  (1e-53, 34%) |
| **Cholesterol / Vitamin D3 / Sterol ester biosynthesis** | | | | | | |
| Sterol-4α-carboxylate 3-dehydrogenase, decarboxylating (ERG26),  E.C. 1.1.1.170 | 16103  KA660000 | 1,655 bp  41.7/kb | 221 aa | AAH93332, *Danio rerio*  (1e-61, 50%) | EFN59037  (2e-78, 55%) | EDP00766  (3e-58, 48%) |
|  | 30098  KA660001 | 822 bp  2.4/kb | 124 aa  M | EFJ50914, *Volvox carteri f. nagariensis* (2e-24, 48%) | EFN59037  (3e-26, 44%) | EDP00766  (3e-32, 52%) |
| Vitamin D 25-hydroxylase (CYP2R1), E.C. 1.14.13.15 | 33651  KA660002 | 1,423 bp  9.8/kb | 213 aa  S | CBY31331, *Oikopleura dioica*  (2e-20, 33%) | EFN54115  (1e-38, 35%) | EDP06080  (1e-33, 35%) |
| Sterol esterase (LIPA),  E.C. 3.1.1.13 | 16826  KA660003 | 2,006 bp  77.3/kb | 241 aa | EFW46840, *Capsaspora owczarzaki* (8e-49, 41%) | EFN56361  (2e-62, 41%) | EDO96999  (9e-58, 45%) |

## Supplementary Table 10 - Curated contigs for tetraterpenoid biosynthesis in the *B. braunii* Showa transcriptome

Coverage indicated in reads per thousand bases of the curated contig. Protein models may be truncated at either or both ends. Localization as predicted by TargetP: C, chloroplast; M: mitochondrion; S: secretory pathway; NP: no prediction. Best BlastP hit with cutoff at 1e-5. GenBank: excluding *Ch. variabilis* and *C. reinhardtii*, shown separately. NF, not found.

| **Enzyme (symbol)** | **Contig ID, Accession** | **Contig length, coverage** | **Protein length, Localization** | **Best BlastP hit in** | | |
| --- | --- | --- | --- | --- | --- | --- |
|  |  |  |  | **GenBank** | ***Chlorella variabilis*** | ***Chlamydomonas reinhardtii*** |
| Phytoene synthase (CrtB),  E.C. 2.5.1.32 | 11195  KA660004 | 2,389 bp  23.4/kb | 292 aa | ADT65126, *Auxenochlorella protothecoides* (6e-152, 77%) | EFN51796  (5e-153, 75%) | AAT38473  (6e-151, 74%) |
| 15-*cis-*phytoene dehydrogenase (CrtP),  E.C. 1.3.5.5 | 20659  KA100958 | 497 bp  10.1/kb | 153 aa | ABR20878, *Muriella zofingiensis*  (1e-79, 76%) | EFN58267  (8e-80, 77%) | EDP05305  (1e-82, 76%) |
|  | 33757  KA660005 | 932 bp  3.2/kb | 204 aa | EFJ23885, *Selaginella moellendorffii* (3e-95, 72%) | EFN58267  (2e-87, 66%) | EDP05305  (1e-102, 70%) |
| Phytoene desaturase (CrtI),  E.C. 1.3.99.28 | 08609  KA660006 | 1,473 bp  6.8/kb | 368 aa | EAU63551, *Stigmatella aurantiaca* (1e-110, 54%) | EFN59350  (5e-120, 59%) | NF |
|  | 19320  KA660007 | 995 bp  12.1/kb | 239 aa  O | CAN93878, *Sorangium cellulosum*  (2e-105, 64%) | EFN59350  (8e-82, 55%) | EDP07797  (4e-6, 42%) |
|  | 20930  KA660008 | 1,215 bp  9.1/kb | 317 aa | ACO69996, *Micromonas sp*.  (5e-93, 46%) | EFN58814  (4e-116, 56%) | EDO99516  (1e-13, 25%) |
| ζ-carotene desaturase (CrtQ), E.C. 1.3.5.6 | 28815  KA660009 | 1,451 bp  5.7/kb | 346 aa | EFJ50084, *Volvox carteri f. nagariensis* (1e-142, 60%) | EFN58666  (4e-145, 58%) | EDP08879  (3e-149, 61%) |
|  | 40999  KA660010 | 875 bp  10.3/kb | 217 aa  O | ACB53982, *Cyanothece sp.*  (8e-95, 71%) | EFN58666  (9e-58, 49%) | EDP08879  (1e-53, 52%) |
|  | 41352  KA660011 | 1,167 bp  1.7/kb | 218 aa  O | ACQ41838, *Elaeis oleifera*  (7e-65, 63%) | EFN58267  (7e-23, 36%) | EDP07040  (7e-59, 58%) |
|  | 46130  KA660012 | 839 bp  6.0/kb | 198 aa | EFJ44652, *Volvox carteri f. nagariensis* (2e-94, 71%) | EFN50960  (8e-96, 70%) | EDP07040  (1e-96, 70%) |
|  | 46414  KA660013 | 1,137 bp  2.6/kb | 166 aa | EFJ44652, *Volvox carteri f. nagariensis* (1e-52, 64%) | EFN50960  (2e-56, 60%) | EDP07040  (4e-56, 63%) |
| Prolycopene isomerase (CrtH), E.C. 5.2.1.13 | 26661  KA660014 | 796 bp  10.1/kb | 231 aa | EFJ46730, *Volvox carteri f. nagariensis* (2e-103, 63%) | EFN51128  (6e-125, 73%) | EDO99516  (6e-103, 68%) |
|  | 37236  KA660015 | 799 bp  2.5/kb | 146 aa | ACK70676, *Cyanothece sp.*  (4e-63, 65%) | EFN51128  (2e-79, 75%) | EDO99516  (7e-77, 71%) |
|  | 42205  KA660016 | 1,727 bp  12.7/kb | 460 aa | EAW36931, *Lyngbya sp*.  (0.0, 59%) | EFN55141  (0.0, 60%) | EDP01276  (0.0, 62%) |
| Lycopene ε-cyclase (CrtL2),  E.C. 5.5.1.18 | 16494  KA660017 | 2,066 bp  10.2/kb | 507 aa  O | EFJ52930, *Volvox carteri f. nagariensis* (0.0, 61%) | EFN58632  (0.0, 58%) | EDP08506  (0.0, 61%) |
| Lycopene β-cyclase (CrtY),  E.C. 5.5.1.19 | 42067  KA660018 | 2,658 bp  6.4/kb | 462 aa  O | CBH31264, *Muriella zofingiensis*  (0.0, 68%) | EFN58632  (2e-72, 37%) | AAX54906  (0.0, 61%) |
| β-carotene 3-hydroxylase (CrtR), E.C. 1.14.13.129 | 09778  KA660019 | 2,518 bp  94.1/kb | 188 aa | EFJ41365, *Volvox carteri f. nagariensis* (3e-69, 63%) | EFN54530  (3e-69, 62%) | EDO99830  (2e-72, 58%) |
|  | 13639  KA660020 | 1,167 bp  21.4/kb | 161 aa  M | CBI22660, *Vitis vinifera*  (6e-56, 56%) | EFN54530  (1e-62, 55%) | EDO99830  (1e-57, 54%) |
| Carotene ε-monooxygenase (LUT1), E.C. 1.14.99.45 | 21025  KA660021 | 804 bp  8.7/kb | 224 aa | EDQ51253, *Physcomitrella patens* ssp. patens (3e-85, 59%) | EFN56967  (6e-68, 44%) | EDO98047  (2e-79, 53%) |
|  | 21510  KA660022 | 833 bp  7.2/kb | 113 aa | EEF50061, *Ricinus communis*  (1e-24, 46%) | EFN52906  (1e-23, 41%) | EDO98047  (6e-28, 45%) |
|  | 37729  KA660023 | 772 bp  2.6/kb | 218 aa | EFJ43400, *Volvox carteri f. nagariensis* (1e-93, 71%) | EFN56967  (8e-125, 78%) | ABQ59243  (2e-105, 67%) |
|  | 43428  KA660024 | 1,353 bp  5.9/kb | 224 aa | EFJ05918, *Selaginella moellendorffii* (4e-110, 69%) | EFN56967  (2e-110, 70%) | ABQ59243  (1e-124, 76%) |
| Zeaxanthin epoxidase (ZEP),  E.C. 1.14.13.90 | 11906  KA660025 | 4,560 bp  21.5/kb | 620 aa | EFJ45294, *Volvox carteri f. nagariensis* (0.0, 59%) | EFN52633  (0.0, 58%) | EDO99435  (7e-16, 30%) |
| Violaxanthin de-epoxidase (VDE), E.C. 1.10.99.3 | 17236  KA660026 | 1,808 bp  16.1/kb | 342 aa | EFJ12716, *Selaginella moellendorffii* (3e-102, 49%) | EFN55190  (3e-130, 57%) | EDP02194  (7e-5, 22%) |
|  | 29174  KA660027 | 1,553 bp  12.2/kb | 364 aa | EDQ52915, *Physcomitrella patens* ssp. patens (2e-133, 57%) | EFN53021  (2e-112, 51%) | EDP02194  (2e-105, 50%) |

## Supplementary Table 11 - Curated contigs for meroterpenoid quinone biosynthesis in the *B. braunii* Showa transcriptome

Coverage indicated in reads per thousand bases of the curated contig. Protein models may be truncated at either or both ends. Localization as predicted by TargetP: C, chloroplast; M: mitochondrion; S: secretory pathway; NP: no prediction. Best BlastP hit with cutoff at 1e-5. GenBank: excluding *Ch. variabilis* and *C. reinhardtii*, shown separately. *, curated contig that may have originated from a transcript of a *B. braunii* cohabitant organism. NF, not found.

| **Enzyme (symbol)** | **Contig ID, Accession** | **Contig length, coverage** | **Protein length, Localization** | **Best BlastP hit in** | | |
| --- | --- | --- | --- | --- | --- | --- |
|  |  |  |  | **GenBank** | ***Chlorella variabilis*** | ***Chlamydomonas reinhardtii*** |
| **Chlorophyll biosynthesis** | | | | | | |
| Geranylgeranyl reductase (ChlP), E.C. 1.3.1.83 | 29523  KA109952 | 681 bp  4.4/kb | 126 aa | EFJ46118, *Volvox carteri f. nagariensis* (5e-61, 75%) | EFN52384  (1e-52, 70%) | EDO99128  (7e-65, 75%) |
|  | 30757  KA111288 | 1,769 bp  108.5/kb | 504 aa  C | EFJ40798, *Volvox carteri f. nagariensis* (0.0, 81%) | EFN59338  (0.0, 76%) | EDP09986  (0.0, 80%) |
|  | 42805  KA660033 | 555 bp  10.8/kb | 141 aa | EFJ46118, *Volvox carteri f. nagariensis* (9e-67, 72%) | EFN52384  (5e-67, 68%) | EDO99128  (2e-71, 71%) |
| Chlorophyll synthase (ChlG),  E.C. 2.5.1.62 | 23089  KA660034 | 1,270 bp  30.7/kb | 314 aa | EFJ49627, *Volvox carteri f. nagariensis* (5e-169, 75%) | EFN57184  (8e-177, 78%) | EDO97346  (7e-171, 74%) |
| **Alpha-tocopherol (Vitamin E) and plastoquinone-9 biosynthesis** | | | | | | |
| Homogentisate phytyltransferase (HPT) | 12096  KA091828 | 3,089 bp  21.7/kb | 166 aa | ACN81039, *Linum usitatissimum*  (5e-55, 51%) | EFN55723  (4e-65, 59%) | EDP00922  (6e-40, 50%) |
| Tocopherol cyclase (VTE1) | 09831  KA660036 | 844 bp  17.8/kb | 137 aa | EFJ42521, *Volvox carteri f. nagariensis* (2e-37, 47%) | EFN58421  (4e-37, 43%) | EDP09189  (5e-44, 51%) |
|  | 14181  KA660037 | 2,494 bp  21.2/kb | 432 aa  M | EFJ42521, *Volvox carteri f. nagariensis* (7e-146, 54%) | EFN58421  (3e-165, 56%) | EDP09189  (5e-141, 51%) |
|  | 32808  KA660038 | 931 bp  4.3/kb | 257 aa | EDQ64388, *Physcomitrella patens ssp. patens* (6e-67, 46%) | EFN58421  (6e-86, 53%) | EDP09189  (2e-58, 39%) |
| Tocopherol methyltransferase (GTMT), E.C. 2.1.1.95 | 12668  KA660042 | 1,403 bp  17.1/kb | 94 aa | EFJ46509, *Volvox carteri f. nagariensis* (3e-22, 55%) | EFN52196  (5e-29, 60%) | EDP03742  (2e-28, 56%) |
|  | 17153  KA660040 | 681 bp  10.3/kb | 224 aa  C | EFJ46509, *Volvox carteri f. nagariensis* (9e-112, 72%) | EFN52196  (1e-101, 63%) | EDP03742  (2e-11, 70%) |
|  | 23079  KA660039 | 2,888 bp  58.2/kb | 344 aa | EFJ47452, *Volvox carteri f. nagariensis* (5e-151, 67%) | EFN52937  (3e-159, 70%) | EDP02465  (2e-155, 64%) |
|  | 23134  KA660041 | 1,833 bp  9.3/kb | 324 aa | EFJ46509, *Volvox carteri f. nagariensis* (1e-154, 67%) | EFN52196  (7e-144, 64%) | EDP03742  (3e-153, 65%) |
| Homogentisate solanesyltransferase (HST) | 09780  KA660035 | 2,504 bp  17.6/kb | 302 aa | EFJ40189, *Volvox carteri f. nagariensis* (5e-143, 71%) | EFN55983  (2e-148, 72%) | EDP01599  (5e-150, 71%) |
| Methylsolanyl-benzoquinone methyltransferase (VTE3) | NF |  |  |  |  |  |
| **Phylloquinone (vitamin K1) and menaquinone (vitamin K2) biosynthesis** | | | | | | |
| Dihydroxynaphthoate octaprenyltransferase (MenA), E.C. 2.5.1.74 | 46175  KA127770 | 1,321 bp  13.6/kb | 302 aa | ACG46195, *Zea mays*  (5e-69, 43%) | EFN57404  (7e-74, 46%) | NF |
| Phylloquinone / menaquinone methyltransferase (UbiE),  E.C. 2.1.1.163 | 37242  KA660047 | 2,323 bp  21.1/kb | 185 aa | ACO66628, *Micromonas sp*.  (6e-78, 65%) | EFN54437  (1e-35, 39%) | EDO99643  (6e-82, 68%) |
|  | 41145  KA660048 | 938 bp  3.2/kb | 103 aa | ACA99102, *Synechococcus sp*.  (4e-24, 47%) | EFN54843  (2e-26, 66%) | EDP06889  (1e-28, 53%) |
| **Ubiquinone (coenzyme Q) biosynthesis** | | | | | | |
| 4-hydroxybenzoate hexaprenyltransferase (Coq2), E.C. 2.5.1.39 | 03304  KA660043 | 859 bp  4.7/kb | 159 aa | EEE68474, *Oryza sativa*  (7e-42, 53%) | EFN55161  (1e-43, 72%) | EDP01665  (2e-43, 77%) |
|  | 16738  KA096859 | 785 bp  6.4/kb | 182 aa | CAL57006, *Ostreococcus tauri*  (2e-76, 66%) | EFN55161  (2e-75, 66%) | EDP01665  (3e-56, 61%) |
| Hexaprenyldihydroxybenzoate methyltransferase (Coq3),  E.C. 2.1.1.114 | 13120  KA660044 | 2,009 bp  8.0/kb | 178 aa | ACF83432, *Zea mays*  (2e-40, 45%) | EFN58414  (5e-41, 53%) | EDP06526  (4e-27, 50%) |
| Ubiquinone biosynthesis monoonxygenase (Coq6),  E.C. 1.14.13.- | 31266  KA660045 | 1,728 bp  9.8/kb | 339 aa | EFJ42741, *Volvox carteri f. nagariensis* (8e-95, 50%) | EFN59911  (5e-91, 45%) | EDP07603  (1e-92, 45%) |
|  | 35102*  KA116039 | 304 bp  9.9/kb | 76 aa | EEY18597, *Verticillium albo-atrum* (3e-34, 74%) | NF | EDP07603  (3e-5, 29%) |
| Ubiquinone biosynthesis methyltransferase (Coq5),  E.C. 2.1.1.201 | 14040*  KA093936 | 487 bp  22.6/kb | 35 aa | EDJ99095, *Magnaporthe grisea*  (6e-16, 97%) | EFN54579  (3e-5, 59%) | NF |
| Ubiquinone biosynthesis monooxygenase (Coq7),  E.C. 1.14.13.- | 10350  KA660046 | 1,295 bp  10.8/kb | 119 aa | EFX88827, *Daphnia pulex*  (3e-12, 31%) | EFN52658  (5e-13, 42%) | NF |
|  | 18835*  KA099077 | 355 bp  47.9/kb | 28 aa | EGO54538, *Neurospora tetrasperma* (7e-18, 89%) | NF | NF |

## Supplementary Table 12 – Curated contigs for the biosynthesis of gibberellic acid diterpenes in the *B. braunii* Showa transcriptome

Coverage indicated in reads per thousand bases of the curated contig. Protein models may be truncated at either or both ends. Localization as predicted by TargetP: C, chloroplast; M: mitochondrion; S: secretory pathway; NP: no prediction. Best BlastP hit with cutoff at 1e-5. GenBank: excluding *Ch. variabilis* and *C. reinhardtii*, shown separately. *, curated contig that may have originated from a transcript of a *B. braunii* cohabitant organism. NF, not found.

| **Enzyme (symbol)** | **Contig ID, Accession** | **Contig length, coverage** | **Protein length, Localization** | **Best BlastP hit in** | | |
| --- | --- | --- | --- | --- | --- | --- |
|  |  |  |  | **GenBank** | ***Chlorella variabilis*** | ***Chlamydomonas reinhardtii*** |
| *ent-*copalyl diphosphate synthase (CPS1),  E.C. 5.5.1.13 | NF |  |  |  |  |  |
| *ent-*kaurene synthase (GA2), E.C. 4.2.3.19 | NF |  |  |  |  |  |
| *ent*-kaurene oxidase (EKOX), E.C. 1.14.13.78 | 22291  KA660028 | 1,363 bp  2.2/kb | 138 aa | EFJ42950, *Volvox carteri f. nagariensis* (7e-17, 38%) | EFN57760  (5e-30, 43%) | EDP06080  (7e-20, 36%) |
| *ent*-kaurenoic acid hydroxylase (KAO),  E.C. 1.14.13.79 | 45316  KA660031 | 532 bp  16.9/kb | 116 aa | BAJ95047, *Hordeum vulgare ssp. vulgare* (3e-13, 43%) | EFN55593  (1e-27, 47%) | EDP07323  (2e-17, 38%) |
| Gibberellin-44 dioxygenase (G44OX), E.C. 1.14.11.12 | 21353  KA660029 | 690 bp  4.3/kb | 158 aa | CBJ26610, *Ectocarpus siliculosus*  (2e-44, 49%) | EFN52694  (3e-41, 39%) | EDP08138  (3e-41, 44%) |
| Gibberellin 3β-dioxygenase (G20OX), E.C. 1.14.11.15 | 16798  KA660030 | 1,375 bp  30.5/kb | 371 aa  O | CBN78512, *Ectocarpus siliculosus*  (2e-78, 41%) | EFN50719  (2e-101, 53%) | EDP08138  (5e-29, 28%) |

## Supplementary Table 13 – Machine-assembled contigs for *S­*-adenosylmethionine regeneration in the *B. braunii* Showa transcriptome

Coverage indicated in reads per thousand bases of the curated contig. Accession numbers refer to the GenBank Transcriptome Shotgun Assembly Sequence Database. Best BlastX hit with cutoff at 1e-5. GenBank: excluding *Ch. variabilis* and *C. reinhardtii*, shown separately. *, curated contig that may have originated from a transcript of a *B. braunii* cohabitant organism. NF, not found.

| **Enzyme (symbol)** | **Contig ID,**  **Accession** | **Contig length, coverage** | **Best BlastX hit in** | | |
| --- | --- | --- | --- | --- | --- |
|  |  |  | **GenBank** | ***Chlorella variabilis*** | ***Chlamydomonas reinhardtii*** |
| *S*-adenosyl-L-homocysteine hydrolase (AhcY), E.C. 3.3.1.1 | 06825  KA130381 | 807 bp  3.7/kb | CCC55421, *Pinus pinaster*  (2e-22, 85%) | EFN51251  (3e-26, 85%) | EDP03365  (2e-24, 81%) |
|  | 07109  KA130692 | 540 bp  54.6/kb | ABA01148, *Chlamydomonas incerta*  (9e-41, 88%) | EFN51251  (2e-44, 91%) | EDP03365  (2e-43, 88%) |
|  | 28237  KA108551 | 510 bp  1,392.2/kb | CAL55423, *Ostreococcus tauri*  (1e-07, 70%) | EFN51251  (5e-11, 68%) | EDP03365  (1e-12, 68%) |
|  | 43141  KA124467 | 1,622 bp  701.0/kb | EFJ48630, *Volvox carteri f. nagariensis*  (0.0, 84%) | EFN51251  (0.0, 85%) | EDP03365  (0.0, 83%) |
| *S*-methyl-methionine:homocysteine *S*-methyltransferase (MmuM), E.C. 2.1.1.10 | 31101  KA111670 | 1,110 bp  7.2/kb | ADH66335, *Nocardiopsis dassonvillei* ssp. dassonvillei (4e-15, 42%) | NF | NF |
| 5-methyltetrahydrofolate : homocysteine *S*-methyltransferase (MetH), E.C. 2.1.1.13 | 05618  KA129091 | 617 bp  6.5/kb | EFJ51715, *Volvox carteri f. nagariensis* (6e-42, 79%) | EFN52253  (3e-55, 89%) | EDP08397  (4e-49, 92%) |
|  | 10339  KA089916 | 1,313 bp  45.7/kb | EEH60712, *Micromonas pusilla*  (0.0, 67%) | EFN52253  (0.0, 69%) | EDP08397  (0.0, 67%) |
|  | 29376  KA109793 | 452 bp  4.4/kb | EFJ51715, *Volvox carteri f. nagariensis* (1e-58, 79%) | EFN52253  (2e-62, 78%) | EDP08397  (3e-59, 89%) |
|  | 34311  KA115200 | 560 bp  3.6/kb | ADE40599, *Candidatus Puniceispirillum marinum*  (4e-07, 55%) | EFN59570  (9e-12, 47%) | EDP08397  (2e-05, 45%) |
|  | 42528  KA123788 | 2,641 bp  8.3/kb | EFJ51715, *Volvox carteri f. nagariensis* (0.0, 73%) | EFN52253  (0.0, 85%) | EDP08397  (0.0, 76%) |
| 5-methyltetrahydropteroyl-triglutamate : homocysteine *S*-methyltransferase (MetE), E.C. 2.1.1.14 | 14237  KA094150 | 1,394 bp  167.1/kb | ABG50257, *Trichodesmium erythraeum* (2e-53, 46%) | EFN57923  (1e-60, 52%) | EDO96787  (3e-63, 52%) |
|  | 26108  KA106349 | 348 bp  1,367.8/kb | ABC98396, *Synechococcus sp.*  (2e-46, 75%) | EFN57923  (3e-44, 70%) | EDO96787  (3e-52, 77%) |
|  | 26820  KA107036 | 834 bp  21.6/kb | CCE41730, *Candida parapsilosis*  (1e-14, 83%) | EFN57923  (5e-17, 75%) | Q39586  (1e-12, 80%) |
|  | 27170  KA107405 | 605 bp  209.9/kb | ABC98396, *Synechococcus sp.*  (2e-18, 42%) | EFN57923  (1e-27, 54%) | EDO96787  (3e-25, 67%) |
|  | 27183  KA107418 | 670 bp  91.0/kb | ABD02841, *Synechococcus sp*.  (4e-24, 53%) | EFN57923  (9e-33, 58%) | EDO96787  (2e-33, 52%) |
|  | 29136  KA109532 | 592 bp  1,184.1/kb | AAD00267, *Chlamydomonas moewusii* (2e-08, 57%) | EFN57923  (8e-15, 62%) | EDO96787  (3e-13, 63%) |
|  | 29228  KA109633 | 668 bp  1,092.8/kb | AEB11899, *Marinithermus hydrothermalis* (3e-36, 55%) | EFN57923  (7e-50, 74%) | EDO96787  (5e-49, 73%) |
|  | 41240  KA122371 | 296 bp  381.8/kb | AAD00267, *Chlamydomonas moewusii* (1e-39, 72%) | EFN57923  (4e-41, 72%) | EDO96787  (2e-47, 77%) |
|  | 41523  KA122680 | 879 bp  4.6/kb | ZP_06381808, *Arthrospira platensis*  (2e-11, 52%) | EFN57923  (5e-15, 59%) | EDO96787  (9e-16, 50%) |
|  | 45035  KA126528 | 635 bp  436.2/kb | ABG50257, *Trichodesmium erythraeum* (6e-42, 44%) | EFN57923  (6e-57, 49%) | EDO96787  (4e-58, 46%) |
|  | 25667*  KA106007 | 262 bp  7.6/kb | EFQ29412, *Glomerella graminicola*  (4e-37, 93%) | EFN57923  (3e-21, 49%) | Q39586  (2e-21, 59%) |
|  | 45290*  KA126796 | 488 bp  10.2/kb | EDR12893, *Laccaria bicolor*  (2e-93, 94%) | EFN57923  (1e-62, 63%) | EDO96787  (2e-61, 62%) |
| *S*-adenosylmethionine synthase (MetK),  E.C. 2.5.1.6 | 06942  KA130510 | 567 bp  3.5/kb | ADI46846, *Volvox carteri f. nagariensis* (7e-27, 72%) | EFN55947  (6e-32, 72%) | EDP08638  (1e-30, 74%) |
|  | 08026  KA131680 | 784 bp  2.6/kb | BAA21726, *Nicotiana tabacum*  (3e-33, 75%) | EFN55947  (9e-39, 76%) | EDP08638  (6e-39, 77%) |
|  | 21356  KA101703 | 502 bp  6.0/kb | ABO99553, *Ostreococcus lucimarinus* (3e-30, 81%) | EFN55947  (4e-32, 76%) | AAB71833  (1e-34, 76%) |
|  | 29206  KA109609 | 444 bp  20.3/kb | CAL57515, *Ostreococcus tauri*  (7e-05, 74%) | EFN55947  (3e-08, 64%) | AAB71833  (4e-07, 75%) |
|  | 41128  KA122248 | 410 bp  12.2/kb | ABA01149, *Chlamydomonas incerta*  (2e-45, 65%) | EFN55947  (5e-50, 65%) | EDP08638  (5e-49, 65%) |

## Supplementary Table 14 – Machine-assembled contigs for fatty acid biosynthesis, desaturation, elongation and TAG assembly in the *B. braunii* Showa transcriptome

Coverage indicated in reads per thousand bases of the curated contig. Accession numbers refer to the GenBank Transcriptome Shotgun Assembly Sequence Database. Best BlastX hit with cutoff at 1e-5. GenBank: excluding *Ch. variabilis* and *C. reinhardtii*, shown separately. *, curated contig that may have originated from a transcript of a *B. braunii* cohabitant organism. NF, not found.

| **Enzyme (symbol)** | **Contig ID, Accession** | **Contig length, coverage** | **Best BlastX hit in** | | |
| --- | --- | --- | --- | --- | --- |
|  |  |  | **GenBank** | ***Chlorella variabilis*** | ***Chlamydomonas reinhardtii*** |
| **Fatty acid biosynthesis** |  |  |  |  |  |
| Type II acetyl-CoA carboxylase, carboxyl transferase alpha subunit (AccA),  E.C. 6.4.1.2 | 41985  KA123187 | 1,817 bp  32.5/kb | EFJ51070, *Volvox carteri f. nagariensis*  (6e-139, 54%) | EFN53883  (3e-145, 52%) | EDP00637  (1e-148, 53%) |
| Type II acetyl-CoA carboxylase, biotin carboxyl carrier protein (AccB),  E.C. 6.4.1.2 | 14856  KA094823 | 618 bp  163.4/kb | ACX52394, *Ammonifex degensii*  (8e-14, 44%) | EFN53301  (6e-17, 45%) | EDO98131  (5e-15, 43%) |
|  | 43154  KA124481 | 747 bp  176.7/kb | EFJ46223, *Volvox carteri f. nagariensis* (1e-28, 69%) | EFN58349  (3e-23, 53%) | EDO98131  (2e-32, 65%) |
| Type II acetyl-CoA carboxylase, biotin carboxylase subunit (AccC), E.C. 6.4.1.2 | 09793  KA133579 | 1306 bp  48.2/kb | EFJ41621, *Volvox carteri f. nagariensis* (0.0, 77%) | EFN58509  (6e-145, 67%) | EDO97049  (0.0, 71%) |
|  | 39819  KA120843 | 273 bp  164.8/kb | AAC41658, *Ricinus communis*  (5e-32, 66%) | EFN58509  (3e-26, 66%) | EDO97049  (9e-28, 64%) |
| Type I acetyl-CoA carboxylase, biotin carboxylase (ACAC), E.C. 6.3.4.14 | 02977  KA110209 | 286 bp  115.4/kb | EDQ62113, *Physcomitrella patens* ssp. patens (8e-35, 65%) | EFN59405  (3e-47, 74%) | NF |
|  | 05373  KA128825 | 589 bp  6.8/kb | BAG63782, *Homo sapiens*  (9e-24, 45%) | EFN59405  (3e-39, 60%) | NF |
|  | 09533  KA133300 | 357 bp  5.6/kb | ABP02013, *Jatropha curcas*  (4e-38, 64%) | EFN59405  (6e-46, 67%) | NF |
|  | 10683  KA090290 | 711 bp  81.6/kb | ACV32315, *Alopecurus japonicas*  (1e-110, 67%) | EFN59405  (3e-106, 69%) | EDO96794  (1e-6, 25%) |
|  | 20490  KA100781 | 718 bp  2.8/kb | AAO32647, *Lolium rigidum*  (3e-36, 65%) | EFN59405  (1e-42, 67%) | EDO97598  (3e-37, 63%) |
|  | 30569  KA111080 | 2,232 bp  23.7/kb | EDQ62113, *Physcomitrella patens* ssp. patens (8e-101, 43%) | EFN59405  (2e-18, 43%) | NF |
|  | 31245  KA111827 | 989 bp  11.1/kb | EFJ43403, *Volvox carteri f. nagariensis* (6e-25, 43%) | EFN59405  (1e-31, 37%) | EDO97599  (1e-10, 71%) |
|  | 32362  KA113057 | 326 bp  52.1/kb | EAT42106, *Aedes aegypti*  (1e-17, 53%) | EFN59405  (3e-24, 56%) | NF |
|  | 34072  KA114936 | 1,161 bp  3.4/kb | ABA01005, *Brassica rapa*  (9e-79, 59%) | EFN59405  (2e-100, 76%) | EDO97598  (1e-46, 63%) |
| Acyl carrier protein, type 1 (ACP1) | 15162  KA095150 | 1,259 bp  92.9/kb | AAU93953, *Helicosporidium sp*.  (5e-30, 55%) | EFN58407  (1e-33, 58%) | EDO98915  (1e-24, 49%) |
|  | 34786*  KA115717 | 350 bp  14.3/kb | EGY19223*, Verticillium dahliae*  (3e-37, 88%) | EFN58407  (1e-22, 53%) | EDO98915  (3e-21, 51%) |
| Acyl carrier protein, type 2 (ACP2) | 10380  KA089961 | 1,276 bp  56.4/kb | EFJ46361, *Volvox carteri f. nagariensis* (3e-15, 61%) | EFN58549  (1e-20, 58%) | EDP09036  (3e-20, 64%) |
|  | 35624  KA116586 | 368 bp  725.5/kb | EEF51709, *Ricinus communis*  (3e-05, 33%) | EFN58549  (8e-06, 38%) | EDP09036  (3e-06, 22%) |
| ACP:*S*-malonyltransferase (FabD),  E.C. 2.3.1.39 | 09966  KA133769 | 1,610 bp  49.7/kb | EFJ49352, *Volvox carteri f. nagariensis* (2e-162, 75%) | EFN58739  (7e-165, 72%) | EDP09600  (3e-102, 54%) |
| β-ketoacyl:ACP synthase III (FabH),  E.C. 2.3.1.180 | 23202  KA103692 | 1,312 bp  19.1/kb | EFJ47899, *Volvox carteri f. nagariensis* (5e-106, 62%) | EFN55645  (3e-124, 66%) | EDO96631  (7e-89, 54%) |
| β-ketoacyl:ACP synthase I (FabB),  E.C. 2.3.1.41, and  β-ketoacyl:ACP synthase II (FabF),  E.C. 2.3.1.179 | 14404  KA094331 | 1,814 bp  70.0/kb | EDQ66274, *Physcomitrella patens* ssp. patens (2e-45, 70%)  FX085405, *Botryococcus braunii* BOT-22 (97 aa, 1e-40, 98%) | EFN55629  (4e-49, 67%) | EDO98446  (2e-45, 65%) |
|  | 18212  KA098453 | 1,164 bp  18.9/kb | EFJ47888, *Volvox carteri f. nagariensis* (6e-86, 65%) | EFN51756  (7e-76, 70%) | EDO98446  (1e-87, 63%) |
|  | 30368  KA110859 | 1,721 bp  127.3/kb | EFJ42114, *Volvox carteri f. nagariensis* (0.0, 73%) | EFN51756  (0.0, 72%) | EDO97709  (0.0, 66%) |
| β-ketoacyl:ACP reductase (FabG),  E.C. 1.1.1.100 | 11964  KA091684 | 1,471 bp  15.6/kb | EFJ44673, *Volvox carteri f. nagariensis* (5e-88, 57%) | EFN57570  (2e-107, 64%) | EDP07053  (6e-102, 62%) |
|  | 22996  KA103465 | 1,583 bp  53.1/kb | EFJ52014, *Volvox carteri f. nagariensis* (1e-107, 71%) | EFN55175  (7e-115, 73%) | EDP06155  (2e-113, 71%) |
|  | 30787  KA111321 | 862 bp  34.8/kb | EFJ48538, *Volvox carteri f. nagariensis* (2e-90, 62%) | EFN55175  (4e-40, 39%) | EDO98712  (4e-90, 60%) |
|  | 31240  KA111822 | 2,009 bp  18.9/kb | EFJ40176, *Volvox carteri f. nagariensis* (1e-67, 56%) | EFN54494  2e-64, 53%) | EDP09766  (4e-75, 57%) |
|  | 18162*  KA098398 | 549 bp  58.3/kb | EEY22923, *Verticillium albo-atrum*  (2e-22, 58%) | EFN55175  (9e-12, 37%) | EDP06155  (4e-13, 40%) |
| β-hydroxyacyl-ACP dehydratase (FabZ),  E.C. 4.2.1.- | 40088  KA121118 | 538 bp  13.0/kb | AAV65356, *Prototheca wickerhamii*  (4e-48, 52%) | EFN57619  (3e-56, 60%) | EDP03190  (7e-57, 62%) |
| Enoyl-ACP reductase (FabI), E.C. 1.3.1.- | 14797  KA094758 | 1,220 bp  42.6/kb | EFJ42762, *Volvox carteri f. nagariensis* (8e-133, 69%) | EFN60073  (4e-133, 66%) | EDO97343  (9e-105, 69%) |
| Stearoyl-ACP Δ-9 desaturase (DesA),  E.C. 1.14.19.2 | 15201  KA095191 | 380 bp  144.7/kb | ACX71636, *Mychonastes zofingiensis* (4e-15, 57%) | EFN56251  (2e-19, 53%) | EDP04705  (9e-20, 62%) |
|  | 43176  KA124505 | 2,007 bp  378.2/kb | EFJ49192, *Volvox carteri f. nagariensis* (1e-144, 68%) | EFN56251  (6e-144, 66%) | EDP04705  (3e-152, 69%) |
| Oleoyl-ACP hydrolase (FatA),  E.C. 3.1.2.14 | 28633  KA108984 | 742 bp  4.0/kb | EFJ52597, *Volvox carteri f. nagariensis* (2e-37, 41%) | EFN55825  (8e-56, 55%) | EDP08596  (3e-41, 40%) |
| Long chain fatty acid:CoA ligase (FadD),  E.C. 6.2.1.3 | 06077  KA129583 | 402 bp  5.0/kb | ACZ37799, *Sphaerobacter thermophiles* (6e-41, 59%) | EFN52634  (9e-42, 58%) | EDP09111  (1e-17, 34%) |
|  | 10276  KA089848 | 3,604 bp  51.3/kb | CAA96522, *Brassica napus*  (0.0, 51%) | EFN52377  (0.0, 61%) | EDP05282  (6e-81, 68%) |
|  | 11205  KA090857 | 2,041 bp  36.7/kb | EDL94464, *Rattus norvegicus*  (3e-51, 51%) | EFN58840  (8e-54, 47%) | EDP05282  (4e-47, 49%) |
|  | 12941  KA092746 | 887 bp  16.9/kb | EFJ51208, *Volvox carteri f. nagariensis* (7e-79, 47%) | EFN56588  (8e-65, 44%) | EDO96800  (4e-80, 45%) |
|  | 18094  KA098328 | 503 bp  6.0/kb | EFJ49638, *Volvox carteri f. nagariensis* (3e-36, 60%) | EFN50517  (1e-21, 55%) | EDP05022  (5e-38, 56%) |
|  | 23345  KA103844 | 2,463 bp  10.6/kb | ACF17663, *Capsicum annuum*  (1e-122, 51%) | EFN60000  (9e-124, 50%) | EDO96800  (4e-121, 50%) |
|  | 30507  KA111012 | 2,672 bp  25.4/kb | EFJ51208, *Volvox carteri f. nagariensis* (0.0, 58%) | EFN60000  (2e-173, 51%) | EDO96800  (0.0, 54%) |
|  | 43014  KA124327 | 1,302 bp  9.2/kb | EFJ49638, *Volvox carteri f. nagariensis* (2e-22, 47%) | EFN52377  (9e-08, 24%) | EDP05022  (4e-16, 37%) |
|  | 18605*  KA098867 | 376 bp  39.9/kb | EEY21843, *Verticillium albo-atrum*  (3e-28, 62%) | EFN52377  (7e-07, 33%) | EDO96800  (3e-11, 33%) |
| Fatty acid synthase, animal type (FasN),  E.C. 2.3.1.85 | 11017*  KA090654 | 1,669 bp  21.0/kb | XP_002738672, *Saccoglossus kowalevskii* (6e-63, 40%) | EFN55810  (7e-12, 35%) | EDP07994  (3e-10, 42%) |
|  | 13420*  KA093263 | 1,031 bp  21.3/kb | EDS45473, *Culex quinquefasciatus*  (5e-30, 39%) | EFN55810  (4e-15, 28%) | EDP07994  (1e-08, 34%) |
|  | 14909*  KA094880 | 662 bp  16.6/kb | XP_002742027, *Saccoglossus kowalevskii* (2e-09, 51%) | NF | NF |
|  | 18070*  KA098302 | 962 bp  4.2/kb | EDW12339, *Drosophila mojavensis*  (5e-58, 48%) | EFN55810  (3e-26, 50%) | EDP07994  (3e-29, 34%) |
|  | 21789*  KA102167 | 890 bp  5.6/kb | EFN89136, *Harpegnathos saltator*  (7e-43, 43%) | EFN55810  (5e-10, 28%) | EDP07994  (1e-14, 30%) |
|  | 21983*  KA102378 | 823 bp  2.4/kb | EAA12911, *Anopheles gambiae*  (3e-35, 59%) | EFN55810  (1e-09, 33%) | EDP07994  (1e-13, 39%) |
|  | 22817*  KA103270 | 1,811 bp  11.6/kb | XP_396268, *Apis mellifera*  (1e-73, 41%) | EFN55810  (9e-19, 26%) | EDP07994  (1e-22, 31%) |
|  | 31757*  KA112394 | 1,525 bp  5.9/kb | EFV56123, *Trichinella spiralis*  (3e-21, 27%) | NF | NF |
|  | 36632*  KA117684 | 1,068 bp  2.8/kb | EDV19158, *Trichoplax adhaerens*  (4e-14, 67%) | NF | NF |
|  | 36837*  KA117910 | 1,060 bp  2.8/kb | EDV20797, *Trichoplax adhaerens*  (2e-30, 48%) | EFN55810  (5e-07, 31%) | EDP07994  (2e-06, 32%) |
|  | 37483*  KA118625 | 537 bp  3.7/kb | EHB09358, *Heterocephalus glaber*  (3e-13, 40%) | NF | NF |
|  | 46409*  KA128023 | 791 bp  5.1/kb | XP_002937357, *Xenopus tropicalis*  (5e-44, 43%) | EFN55810  (1e-07, 39%) | EDP07994  (3e-10, 37%) |
| Fatty acid synthase, fungal (FAS1),  E.C. 2.3.1.86 | 07064*  KA130643 | 531 bp  3.8/kb | EGO24420, *Serpula lacrymans* var.  lacrymans (5e-61, 66%) | EFN53239  (1e-07, 34%) | EDP05644  (3e-08, 34%) |
|  | 38860*  KA119997 | 261 bp  26.8/kb | EEY14911, *Verticillium albo-atrum*  (1e-15, 86%) | NF | NF |
| **Fatty acid desaturation** | | | | | |
| Ω-6 (Δ-12) fatty acid desaturase (FAD6),  E.C. 1.14.19.- | 10624  KA090228 | 1,096 bp  17.3/kb | EFJ49035, *Volvox carteri f. nagariensis* (4e-82, 61%) | EFN51858  (8e-83, 59%) | EDP03637  (3e-78, 57%) |
|  | 33113  KA113881 | 893 bp  13.4/kb | EFJ49035, *Volvox carteri f. nagariensis* (1e-36, 63%) | EFN51858  (3e-46, 70%) | EDP03637  (7e-43, 63%) |
|  | 10475*  KA090062 | 706 bp  42.5/kb | EHA30740, *Bacillus subtilis* ssp. subtilis (7e-14, 33%) | EFN51858  (1e-05, 28%) | EDP03637  (5e-08, 30%) |
|  | 31434*  KA112037 | 1,064 bp  8.5/kb | ABF89451, *Myxococcus xanthus*  (1e-17, 29%) | EFN50714  (3e-08, 29%) | EDP03637  (5e-10, 26%) |
|  | 43600*  KA124975 | 733 bp  231.9/kb | EGD60400, *Novosphingobium*  *nitrogenifigens* (2e-18, 33%) | EFN50714  (3e-12, 32%) | EDP09401  (2e-13, 27%) |
| Ω-3 (Δ-15) fatty acid desaturase (FAD8),  E.C. 1.14.19.- | 10876  KA090500 | 1,574 bp  56.5/kb | ACD03846, *Parietochloris incise*  (0.0, 75%) | EFN50714  (1e-161, 71%) | EDP09401  (0.0, 70%%) |
| Δ-6 fatty acid desaturase (FADS2),  E.C. 1.14.19.- | 13278  KA093111 | 2,038 bp  8.8/kb | ADB81955, *Parietochloris incise*  (2e-47, 61%) | NF | EDP09855  (4e-10, 28%) |
| **Fatty acid elongation** | | | | | |
| Very long chain fatty acid elongase (ELOVL5), E.C. 2.3.1.- | 19999  KA100257 | 383 bp  122.7/kb | ACR53359, *Pyramimonas cordata*  (3e-26, 45%) | NF | NF |
| β-ketoacyl-CoA reductase (KAR),  E.C. 1.1.1.- | 15622  KA095647 | 1,343 bp  242.7/kb | XP_003579998, *Brachypodium*  *distachyon* (5e-30, 56%) | EFN59097  (2e-31, 53%) | NF |
|  | 32790  KA113525 | 604 bp  46.4/kb | EFW46622, *Capsaspora* *owczarzaki*  (1e-33, 52%) | EFN59097  (5e-39, 48%) | EDP04696  (7e-08, 29%) |
| 3-hydroxyacyl-CoA dehydratase (PHS1),  E.C. 4.2.1.- | 14264  KA094180 | 1,360 bp  73.5/kb | EFJ42903, *Volvox carteri f. nagariensis* (4e-27, 59%) | EFN57865  (1e-30, 56%) | EDP01055  (9e-12, 38%) |
|  | 14707  KA094660 | 1,122 bp  29.4/kb | EFJ42903, *Volvox carteri f. nagariensis* (7e-48, 50%) | EFN57865  (3e-53, 52%) | EDP06194  (9e-34, 45%) |
|  | 43693  KA125077 | 1,575 bp  10.2/kb | EAL68514, *Dictyostelium discoideum* (2e-22, 51%) | EFN59260  (7e-29, 50%) | EDP01055  (1e-23, 39%) |
| Enoyl-CoA reductase (TER), E.C. 1.3.1.- | 19256  KA099492 | 414 bp  111.1/kb | EFJ53040, *Volvox carteri f. nagariensis* (3e-54, 63%) | EFN57949  (1e-68, 66%) | EDO99055  (5e-55, 57%) |
|  | 19361  KA099601 | 447 bp  232.7/kb | AEE79372, *Arabidopsis thaliana*  (6e-16, 61%) | EFN57949  (7e-20, 61%) | EDO99055  (3e-19, 56%) |
|  | 23461  KA103966 | 462 bp  121.2/kb | EFJ53040, *Volvox carteri f. nagariensis* (1e-34, 49%) | EFN57949  (3e-49, 57%) | EDO99055  (5e-39, 52%) |
|  | 29218  KA109622 | 562 bp  7.1/kb | EFJ27492, *Selaginella moellendorffii*  (5e-15, 36%) | EFN57949  (7e-21, 37%) | EDO99055  (1e-11, 28%) |
| Very long chain fatty acyl-CoA hydrolase, E.C. 3.1.2- | NF |  |  |  |  |
| **TAG biosynthesis** | | | | | |
| Glycerol kinase (GlpK), E.C. 2.7.1.30 | 11895  KA091608 | 1,178  13.6/kb | EDQ71347, *Physcomitrella patens* ssp. patens (3e-24, 48%) | EFN59182  (2e-34, 69%) | EDP04155  (2e-21, 65%) |
|  | 12844  KA092642 | 983 bp  7.1/kb | EFJ47782, *Volvox carteri f. nagariensis* (7e-106, 61%) | EFN59182  (1e-119, 67%) | EDP04155  (9e-108, 63%) |
|  | 18792*  KA099036 | 253 bp  15.8/kb | EGU81741, *Fusarium oxysporum*  (5e-23, 83%) | EFN59182  (2e-08, 51%) | EDP04155  (7e-11, 52%) |
| Glycerol-3-phosphate *O*-acyltransferase (GPAT), E.C. 2.3.1.15 | 05783  KA129267 | 279 bp  7.2/kb | ACT32030, *Vernicia fordii*  (5e-16, 68%) | EFN53048  (4e-05, 27%) | EDP08445  (1e-19, 58%) |
|  | 08414  KA132095 | 500 bp  8.0/kb | EFJ53211, *Volvox carteri f. nagariensis* (9e-66, 59%) | EFN54897  (4e-11, 31%) | EDP08445  (1e-69, 57%) |
|  | 13785  KA093658 | 1,005 bp  8.0/kb | EEC82177, *Oryza sativa*  (2e-73, 72%) | EFN54897  (3e-08, 49%) | EDP08445  (4e-55, 61%) |
|  | 16194  KA096265 | 722 bp  24.9/kb | EFJ48252, *Volvox carteri f. nagariensis* (1e-85, 53%) | NF | EDP02129  (2e-87, 52%) |
| Lysophosphatidic acid acyltransferase (LPAAT), E.C. 2.3.1.51 | 11667  KA091361 | 1,113 bp  11.7/kb | EDQ61546, *Physcomitrella patens* ssp. patens (2e-52, 51%) | EFN51749  (8e-60, 50%) | EDP02300  (1e-48, 45%) |
|  | 17008  KA097148 | 497 bp  20.1/kb | EEE91930, *Populus trichocarpa*  (4e-24, 58%) | EFN51749  (7e-32, 56%) | NF |
| Phosphatidic acid phosphatase (PAP),  E.C. 3.1.3.4 | 12737  KA092526 | 1,905 bp  16.3/kb | ACN25649, *Zea mays*  (6e-47, 44%) | EFN57390  (3e-69, 54%) | EDO97063  (7e-27, 36%) |
|  | 17100  KA097248 | 1,404 bp  12.8/kb | EEH60336, *Micromonas pusilla*  (4e-26, 38%) | EFN53992  (2e-29, 37%) | EDO97339  (7e-35, 40%) |
|  | 18112  KA098346 | 807 bp  3.7/kb | EDQ81247, *Physcomitrella patens* ssp. patens (9e-10, 36%) | EFN52812  (5e-13, 32%) | EDP01348  (3e-07, 40%) |
| Diacylglycerol acyltransferase (DGAT),  E.C. 2.3.1.20 | 17739  KA097939 | 1,230 bp  3.3/kb | AAT73629, *Glycine max*  (4e-71, 46%) | EFN50697  (5e-84, 54%) | NF |
|  | 42061  KA123273 | 2,857 bp  16.5/kb | EFJ51805, *Volvox carteri f. nagariensis* (7e-38, 33%) | EFN51306  (4e-18, 25%) | EDP02056  (1e-41, 36%) |
|  | 00393*  KA120369 | 353 bp  28.3/kb | EFQ28516, *Glomerella graminicola*  (2e-39, 89%) | EFN50697  (5e-10, 39%) | NF |
| Phospholipid:diacylglycerol acyltransferase (PDAT), E.C. 2.3.1.158 | 23371  KA103871 | 980 bp  18.4/kb | EEH56370, *Micromonas pusilla*  (1e-72, 51%) | EFN56524  (9e-07, 22%) | EDP07444  (3e-38, 50%) |
|  | 28074  KA108373 | 749 bp  8.0/kb | EER90937, *Sorghum bicolor*  (2e-27, 38%) | EFN56524  (3e-14, 34%) | EDP07444  (1e-05, 34%) |
|  | 31539  KA112153 | 2,226 bp  19.8/kb | EER26236, *Coccidioides posadasii*  (1e-26, 44%) | NF | EDP07444  (7e-23, 49%) |
| Major lipid droplet protein (MLDP) | 35177  KA116117 | 572 bp  295.5/kb | AEW43285, *Dunaliella bardawil*  (2e-10, 34%) | EFN52470  (8e-07, 30%) | EDP00062  (3e-10, 33%) |
|  | 42893  KA124192 | 2,018 bp  7.4/kb | NF | NF | EDP00062  (3e-06, 38%) |
|  | 33156  KA113927 | 1,082 bp  49.9/kb | ADN95182, *Haematococcus pluvialis*  (1e-08, 31%) | EFN52470  (8e-06, 29%) | NF |
|  | 07772  KA131406 | 1,291 bp  252.5/kb | ADN95182, *Haematococcus pluvialis* (2e-15, 25%) | EFN52470  (1e-41, 35%) | EDP00062  (7e-19, 26%) |
|  | 23127  KA103609 | 2,327 bp  237.6/kb | ADN95182, *Haematococcus pluvialis* (2e-11, 23%) | EFN52470  (5e-18, 28%) | NF |

## Supplementary Table 15 – Machine-assembled contigs for starch and cellulose biosynthesis in the *B. braunii* Showa transcriptome

Coverage indicated in reads per thousand bases of the curated contig. Accession numbers refer to the GenBank Transcriptome Shotgun Assembly Sequence Database. Best BlastX hit with cutoff at 1e-5. GenBank: excluding *Ch. variabilis* and *C. reinhardtii*, shown separately. *, curated contig that may have originated from a transcript of a *B. braunii* cohabitant organism. NF, not found.

| **Enzyme (symbol)** | **Contig ID, Accession** | **Contig length, coverage** | **Best BlastX hit in** | | |
| --- | --- | --- | --- | --- | --- |
|  |  |  | **GenBank** | ***Chlorella variabilis*** | ***Chlamydomonas reinhardtii*** |
| **Starch biosynthesis** |  |  |  |  |  |
| Hexokinase (HK), E.C. 2.7.1.1 | 13089  KA092906 | 2,002 bp  7.0/kb | EEE93920, *Populus trichocarpa*  (9e-39, 37%) | EFN54251  (1e-12, 33%) | EDO96927  (7e-33, 38%) |
|  | 19984  KA100242 | 766 bp  9.1/kb | ABR18277, *Picea sitchensis*  (1e-31, 35%) | EFN54251  (3e-21, 37%) | EDO96927  (4e-32, 35%) |
|  | 43562  KA124932 | 842 bp  24.9/kb | CAC81350, *Solanum lycopersicum*  (8e-41, 41%) | EFN54251  (1e-12, 49%) | EDO96927  (3e-28, 38%) |
|  | 00080*  KA131650 | 295 bp  37.3/kb | EGY22649, *Verticillium dahliae*  (3e-14, 76%) | NF | NF |
|  | 24299*  KA104855 | 345 bp  43.5/kb | EGY19897, *Verticillium dahliae*  (7e-26, 61%) | NF | NF |
| Glucokinase (Glk), E.C. 2.7.1.2 | 03075  KA111280 | 899 bp  12.2/kb | EEH58649, *Micromonas pusilla*  (1e-35, 47%) | NF | EDP09343  (1e-47, 48%) |
| Phosphoglucomutase (PGM), E.C. 5.4.2.2 | 00848  KA132166 | 576 bp  48.6/kb | EGI21888, *Verticillium dahliae*  (5e-44, 90%) | EFN54324  (7e-16, 56%) | EDO99577  (3e-25, 57%) |
|  | 03487  KA115799 | 572 bp  5.2/kb | EFX85018, *Daphnia pulex*  (3e-50, 56%) | EFN54976  (3e-51, 54%) | EDP09622  (8e-53, 56%) |
|  | 16061  KA096123 | 1,197 bp  10.0/kb | EDQ82715, *Physcomitrella patens* ssp. patens (7e-140, 67%) | EFN54324  (3e-97, 56%) | EDO99577  (2e-117, 60%) |
|  | 19700  KA099942 | 514 bp  33.1/kb | EGO22445, *Serpula lacrymans* var.  lacrymans (2e-44, 55%) | EFN54324  (3e-14, 52%) | EDO99577  (1e-47, 57%) |
|  | 30614  KA111131 | 2,566 bp  35.5/kb | EFJ52896, *Volvox carteri f. nagariensis*  (0.0, 68%) | EFN54324  (6e-124, 71%) | EDO99577  (0.0, 67%) |
|  | 04179*  KA122970 | 719 bp  2.8/kb | XP_003391495, *Amphimedon queenslandica* (1e-37, 43%) | EFN54976  (1e-38, 45%) | EDP09622  (1e-30, 51%) |
| Glucose-1-phosphate adenylyltransferase (GlgC), E.C. 2.7.7.27 | 08498  KA132186 | 663 bp  4.5/kb | EDQ67182, *Physcomitrella patens* ssp. patens (3e-05, 52%) | EFN50814  (2e-09, 51%) | EDP08701  (4e-10, 50%) |
|  | 10970  KA090601 | 1,862 bp  51.6/kb | CAL56698, *Ostreococcus tauri*  (2e-49, 34%) | EFN56332  (9e-50, 34%) | EDP08701  (9e-43, 31%) |
|  | 14111  KA094013 | 811 bp  22.2/kb | NF | NF | EDP08701  (5e-06, 59%) |
|  | 16448  KA096541 | 662 bp  10.6/kb | NF | NF | EDP04406  (2e-05, 29%) |
|  | 18148  KA098384 | 700 bp  8.6/kb | AAP99886, *Prochlorococcus marinus* ssp. marinus (7e-23, 50%) | EFN56332  (4e-30, 45%) | EDP08701  (1e-25, 43%) |
|  | 30810  KA111348 | 1,969 bp  30.0/kb | ABP00699, *Ostreococcus lucimarinus* (0.0, 67%) | EFN50814  (0.0, 62%) | EDP08701  (0.0, 66%) |
|  | 30843  KA111383 | 2,130 bp  49.3/kb | EFJ42808, *Volvox carteri f. nagariensis* (0.0, 70%) | EFN50814  (8e-155, 57%) | EDP04344  (0.0, 64%) |
|  | 32980  KA113734 | 1,433 bp  9.1/kb | EFJ04435, *Selaginella moellendorffii*  (1e-61, 40%) | EFN50814  (2e-56, 37%) | EDP08701  (2e-56, 39%) |
|  | 46182  KA127778 | 589 bp  8.5/kb | ACL47068, *Cyanothece sp*.  (1e-09, 51%) | EFN50814  (2e-13, 45%) | EDP04344  (9e-13, 41%) |
| Starch synthase (GlgA), E.C. 2.4.1.21 | 06082  KA129589 | 572 bp  10.5/kb | EFJ50113, *Volvox carteri f. nagariensis* (5e-65, 56%) | EFN55259  (4e-48, 80%) | EDP08815  (2e-70, 55%) |
|  | 07623  KA131245 | 487 bp  4.1/kb | EFJ41340, *Volvox carteri f. nagariensis* (2e-24, 56%) | EFN59872  (1e-23, 53%) | EDO99379  (2e-29, 56%) |
|  | 15308  KA095307 | 960 bp  7.3/kb | CAX51357, *Hordeum vulgare* ssp. vulgare (3e-82, 50%) | EFN59970  (3e-47, 38%) | EDP00740  (5e-81, 53%) |
|  | 17434  KA097611 | 697 bp  2.9/kb | EFJ17036, *Selaginella moellendorffii*  (1e-42, 55%) | EFN55259  (9e-24, 46%) | AAY42381  (9e-27, 86%) |
|  | 29962  KA110413 | 604 bp  5.0/kb | ABN48659*, Triticum aestivum*  (4e-43, 65%) | EFN59872  (7e-46, 72%) | AAC17970  (2e-47, 81%) |
|  | 30919  KA111467 | 1,984 bp  8.6/kb | EFJ40335, *Volvox carteri* f. *nagariensis* (0.0, 62%) | EFN55259  (0.0, 61%) | AAY42381  (0.0, 71%) |
|  | 31914  KA112566 | 890 bp  18.0/kb | EFJ50113, *Volvox carteri f. nagariensis* (6e-64, 70%) | EFN55259  (5e-56, 62%) | EDP08815  (5e-69, 69%) |
|  | 32079  KA112748 | 1,040 bp  7.7/kb | EEE86082, *Populus trichocarpa*  (3e-25, 35%) | EFN55259  (5e-16, 47%) | AAY42381  (9e-18, 70%) |
|  | 33219  KA113997 | 1,234 bp  6.5/kb | ACO61951, *Micromonas sp.*  (2e-59, 51%) | EFN53763  (6e-52, 49%) | EDO97466  (2e-54, 48%) |
|  | 38186  KA119394 | 940 bp  3.2/kb | EFJ39907, *Volvox carteri f. nagariensis* (1e-69, 46%) | EFN53763  (2e-84, 69%) | EDO97466  (1e-77, 46%) |
|  | 41923  KA123119 | 3,583 bp  35.7/kb | BAE79814, *Parachlorella kessleri*  (6e-163, 58%) | EFN54355 (1e-123, 70%) | EDP00372  (1e-155, 55%) |
|  | 43465  KA124826 | 1,244 bp  27.3/kb | EFH48362, *Arabidopsis lyrata* ssp.  lyrata (8e-08, 36%) | EFN59872  (5e-11, 34%) | EDP00740  (1e-12, 40%) |
|  | 45448  KA126968 | 317 bp  34.7/kb | AAZ66390, *Arabidopsis thaliana*  (2e-43, 62%) | EFN59970  (2e-38, 54%) | EDO96030  (1e-42, 57%) |
|  | 46208  KA127806 | 1,218 bp  4.1/kb | EFJ41340, *Volvox carteri f. nagariensis* (3e-74, 56%) | EFN59970  (3e-33, 67%) | EDO99379  (1e-74, 53%) |
| 1,4-α-glucan branching enzyme (GlgB),  E.C. 2.4.1.18 | 08586  KA132282 | 871 bp  5.7/kb | EFJ45925, *Volvox carteri f. nagariensis* (8e-56, 49%) | EFN50981  (1e-46, 69%) | EDP02832  (7e-66, 49%) |
|  | 11816  KA091521 | 1,717 bp  15.1/kb | BAF98234, *Parachlorella kessleri*  (0.0, 71%) | EFN58941  (0.0, 62%) | EDP05581  (0.0, 77%) |
|  | 12609  KA092388 | 2,368 bp  8.0/kb | AAZ20130, *Malus x domestica*  (2e-167, 61%) | EFN60069  (9e-157, 80%) | EDP05581  (9e-133, 44%) |
|  | 14148  KA094052 | 1,018 bp  51.1/kb | BAF98234, *Parachlorella kessleri*  (1e-175, 71%) | EFN58941  (5e-178, 69%) | EDP05581  (3e-177, 69%) |
|  | 41168  KA122292 | 987 bp  2.0/kb | ADX46226, *Acidovorax avenae* ssp.  avenae (9e-28, 53%) | EFN50981  (1e-45, 50%) | EDP02832  (1e-42, 52%) |
|  | 00136*  KA093458 | 450 bp  66.7/kb | EGU85161, *Fusarium oxysporum*  (8e-47, 80%) | EFN58941  (2e-22, 43%) | EDP05581  (5e-23, 46%) |
|  | 02076*  KA101066 | 283 bp  7.1/kb | EAQ83852, *Chaetomium globosum*  (3e-35, 86%) | EFN60069  (2e-28, 67%) | EDP08206  (2e-27, 67%) |
|  | 02271*  KA103156 | 300 bp  6.7/kb | EAQ83852, *Chaetomium globosum*  (5e-53, 92%) | EFN60069  (4e-37, 70%) | EDP01649  (6e-34, 62%) |
| Amylo-α-1,6-glucosidase, glycogen debranching enzyme (AGL), E.C. 3.2.1.33 | 13953*  KA093841 | 909 bp  55.0/kb | EFQ36345, *Glomerella graminicola*  (8e-25, 78%) | NF | NF |
| **Cellulose biosynthesis** |  |  |  |  |  |
| UTP:α-D-glucose-1-phosphate uridylyltransferase (UGP), E.C. 2.7.7.9 | 30609  KA111125 | 1,633 bp  22.7/kb | EFJ46986, *Volvox carteri f. nagariensis* (0.0, 67%) | EFN56331  (0.0, 67%) | EDP04196  (0.0, 63%) |
|  | 24769*  KA105247 | 214 bp  14.0/kb | EGU76002, *Fusarium oxysporum*  (8e-14, 75%) | NF | NF |
|  | 43024*  KA124338 | 658 bp  74.5/kb | EFQ29255, *Glomerella graminicola*  (3e-85, 96%) | EFN56331  (5e-19, 35%) | EDP04196  (2e-22, 42%) |
| Cellulose synthase (BcsA), E.C. 2.4.1.12 | 10565  KA090162 | 2,272 bp  6.6/kb | BAJ65324, *Molgula tectiformis*  (1e-51, 32%) | NF | EDP08496  (7e-05, 26%) |
|  | 11061  KA090702 | 1,537 bp  64.4/kb | ACO69823, *Micromonas sp.*  (4e-75, 44%) | EFN51937  (4e-63, 55%) | EDP08496  (3e-20, 51%) |
|  | 20495  KA100786 | 530 bp  3.8/kb | BAB75456, *Nostoc sp.*  (3e-09, 38%) | NF | NF |
|  | 32669  KA113391 | 748 bp  22.7/kb | ACO69823, *Micromonas sp.*  (2e-52, 50%) | EFN51937  (6e-73, 57%) | EDP08496  (2e-32, 50%) |

## Supplementary Table 16 – Machine-assembled contigs for putative ABC transporter pumps in the *B. braunii* Showa transcriptome

Coverage indicated in reads per thousand bases of the curated contig. Accession numbers refer to the GenBank Transcriptome Shotgun Assembly Sequence Database. Best BlastX hit with cutoff at 1e-5. GenBank: excluding *Ch. variabilis* and *C. reinhardtii*, shown separately. *, curated contig that may have originated from a transcript of a *B. braunii* cohabitant organism. NF, not found.

| **Enzyme** | **Contig ID, Accession** | **Contig length, coverage** | **Best BlastX hit in** | | |
| --- | --- | --- | --- | --- | --- |
|  |  |  | **GenBank** | ***Chlorella variabilis*** | ***Chlamydomonas reinhardtii*** |
| ABC transporter, Subfamily A | 04653  KA128050 | 522 bp  7.7/kb | EGZ11630, *Phytophthora sojae*  (7e-13, 29%) | EFN54700  (2e-30, 31%) | NF |
|  | 12678  KA092462 | 1,294 bp  8.5/kb | EGV99481, *Cricetulus griseus*  (6e-57, 48%) | EFN54700  (8e-54, 49%) | EDP00368  (7e-53, 45%) |
|  | 29099  KA109491 | 485 bp  4.1/kb | EHH31333, *Macaca mulatta*  (3e-16, 69%) | EFN57675  (4e-19, 59%) | EDO99096  (2e-19, 49%) |
|  | 42173  KA123397 | 1,444 bp  20.8/kb | EFJ47840, *Volvox carteri f. nagariensis* (4e-83, 53%) | EFN54700  (2e-66, 40%) | EDP00368  (6e-51, 41%) |
| ABC transporter, Subfamily B | 04480  KA126283 | 769 bp  5.2/kb | EFH47064, *Arabidopsis lyrata* ssp. lyrata (1e-74, 50%) | EFN52494  (9e-81, 54%) | EDP00495  (6e-55, 44%) |
|  | 09970  KA133774 | 2,034 bp  14.7/kb | EAZ28624, *Oryza sativa*  (6e-74, 61%) | EFN52494  (1e-70, 60%) | EDP00406  (7e-63, 51%) |
|  | 12912  KA092715 | 692 bp  7.2/kb | EFJ41513, *Volvox carteri f. nagariensis* (8e-37, 49%) | EFN54275  (5e-29, 60%) | EDP04614  (1e-27, 74%) |
|  | 24135  KA104685 | 631 bp  20.6/kb | EEF06006, *Populus trichocarpa*  (1e-48, 72%) | EFN54275  (8e-46, 65%) | EDP04614  (7e-50, 66%) |
|  | 30542  KA111051 | 3,239 bp  27.2/kb | EDQ53872, *Physcomitrella patens* ssp. patens (2e-62, 58%) | EFN54621  (4e-71, 55%) | EDO98725  (3e-68, 64%) |
|  | 31052  KA111615 | 3,460 bp  30.6/kb | ABG56414, *Taxus cuspidata*  (1e-122, 50%) | EFN58606  (4e-137, 56%) | EDP00495  (7e-121, 51%) |
|  | 45924  KA127491 | 771 bp  2.6/kb | ABG56414, *Taxus cuspidata*  (7e-31, 43%) | EFN58606  (8e-29, 60%) | EDP00495  (7e-32, 44%) |
|  | 31055  KA111618 | 2,389 bp  15.1/kb | EEH54358, *Micromonas pusilla*  (7e-130, 49%) | EFN54348  (0.0, 53%) | EDP06881  (7e-118, 49%) |
|  | 31774  KA112413 | 1,168 bp  8.6/kb | EDQ54424, *Physcomitrella patens* ssp. patens (1e-11, 49%) | EFN54621  (2e-18, 46%) | EDP06881  (3e-11, 42%) |
| ABC transporter, Subfamily B, mitochondrial | 12206  KA091950 | 1,165 bp  12.0/kb | EFJ45521, *Volvox carteri f. nagariensis* (2e-100, 67%) | EFN52675  (4e-111, 62%) | EDP00522  (5e-116, 72%) |
|  | 20134  KA100403 | 726 bp  5.5/kb | XP_003245596, *Acyrthosiphon pisum* (2e-33, 41%) | EFN54392  (1e-38, 50%) | EDP05599  (7e-41, 53%) |
|  | 31257  KA111840 | 797 bp  16.3/kb | ABO98832, *Ostreococcus lucimarinus* (2e-23, 57%) | EFN53974  (5e-29, 61%) | EDP00522  (6e-25, 58%) |
|  | 34722  KA115650 | 1,345 bp  5.9/kb | EDQ52704, *Physcomitrella patens* ssp. patens (6e-42, 56%) | EFN53974  (2e-47, 50%) | EDP05599  (4e-23, 35%) |
|  | 35963  KA116948 | 869 bp  2.3/kb | EFJ49390, *Volvox carteri f. nagariensis* (4e-54, 62%) | EFN52666  (9e-56, 45%) | EDO96584  (2e-55, 53%) |
|  | 40914  KA122014 | 745 bp  4.0/kb | EFJ49390, *Volvox carteri f. nagariensis* (3e-55, 47%) | EFN52666  (6e-59, 45%) | EDP05599  (2e-73, 53%) |
|  | 44475  KA125939 | 904 bp  4.4/kb | EEH58719, *Micromonas pusilla*  (5e-78, 65%) | EFN52675  (2e-82, 72%) | EDP00522  (2e-69, 62%) |
|  | 44615  KA126095 | 1,332 bp  8.3/kb | EFV85127, *Achromobacter xylosoxidans* (1e-18, 38%) | EFN54392  (6e-76, 42%) | EDO96584  (5e-68, 56%) |
|  | 45501  KA127028 | 665 bp  6.0/kb | EDV29654, *Trichoplax adhaerens*  (6e-32, 43%) | EFN54392  (8e-38, 41%) | EDP05599  (2e-37, 42%) |
| ABC transporter, Subfamily C | 10083  KA089637 | 978 bp  14.3/kb | EFJ48392, *Volvox carteri f. nagariensis* (3e-70, 49%) | EFN52814  (5e-80, 52%) | EDP07736  (6e-74, 48%) |
|  | 12326  KA092080 | 1,541 bp  8.4/kb | EDV28182, *Trichoplax adhaerens*  (5e-52, 43%) | EFN56057  (1e-59, 48%) | EDP08676  (2e-31, 33%) |
|  | 13729  KA093599 | 968 bp  22.7/kb | EFJ48392, *Volvox carteri f. nagariensis* (1e-90, 55%) | EFN52814  (8e-96, 54%) | AAL35383  (2e-93, 53%) |
|  | 22435  KA102870 | 524 bp  3.8/kb | EFJ44543, *Volvox carteri f. nagariensis* (1e-18, 46%) | EFN56982  (3e-17, 38%) | EDP07482  (1e-24, 49%) |
|  | 27405  KA107656 | 828 bp  4.8/kb | EFJ52174, *Volvox carteri f. nagariensis* (4e-84, 67%) | EFN56982  (2e-85, 62%) | EDP06139  (7e-81, 62%) |
|  | 31311  KA111901 | 2,375 bp  24.0/kb | EFJ52174, *Volvox carteri f. nagariensis* (6e-178, 63%) | EFN56982  (5e-172, 68%) | EDP06139  (1e-164, 58%) |
|  | 31885  KA112533 | 1,705 bp  8.8/kb | AAC49798, *Arabidopsis thaliana*  (4e-46, 55%) | EFN56982  (5e-35, 44%) | EDP01031  (1e-40, 57%) |
|  | 36127  KA117129 | 894 bp  4.5/kb | EFJ48392, *Volvox carteri f. nagariensis* (6e-65, 42%) | EFN52814  (8e-72, 41%) | EDP07736  (8e-69, 41%) |
|  | 41658  KA122825 | 765 bp  2.6/kb | XP_001500757, *Equus caballus*  (2e-19, 47%) | EFN56982  (3e-23, 50%) | EDP08676  (3e-20, 51%) |
|  | 44672  KA126157 | 1,490 bp  5.4/kb | XP_001341895, *Danio rerio*  (2e-46, 49%) | EFN56982  (4e-54, 59%) | EDP06139  (6e-48, 46%) |
|  | 37214*  KA118328 | 569 bp  3.5/kb | EEB95340, *Moniliophthora perniciosa* (7e-57, 78%) | EFN56982  (8e-29, 48%) | EDP06139  (7e-31, 47%) |
| ABC transporter, Subfamily D | 05104  KA128536 | 1,613 bp  7.4/kb | EDQ68747, *Physcomitrella patens* ssp. patens (1e-74, 45%) | EFN57216  (2e-77, 43%) | EDP05816  (6e-43, 34%) |
|  | 07626  KA131248 | 570 bp  7.0/kb | EEF01268, *Populus trichocarpa*  (8e-16, 58%) | EFN57216  (3e-15, 45%) | EDP05816  (2e-15, 62%) |
|  | 11863  KA091574 | 1,152 bp  7.8/kb | EFJ52346, *Volvox carteri f. nagariensis* (1e-33, 78%) | EFN54030  (2e-40, 80%) | EDP05816  (1e-38, 78%) |
|  | 31500  KA112111 | 1,279 bp  18.8/kb | EFJ35787, *Selaginella moellendorffii*  (2e-67, 45%) | EFN57216  (1e-37, 52%) | EDP05816  (4e-46, 40%) |
|  | 32030  KA112695 | 1,634 bp  6.7/kb | EFJ40520, *Volvox carteri f. nagariensis* (5e-94, 58%) | EFN54974  (2e-85, 50%) | EDO97765  (8e-23, 48%) |
|  | 43838  KA125236 | 978 bp  16.4/kb | EFJ40520, *Volvox carteri f. nagariensis* (1e-50, 56%) | EFN54974  (2e-64, 68%) | EDO97765  (3e-45, 50%) |
| ABC transporter, Subfamily G | 06666  KA130216 | 559 bp  3.6/kb | XP_003520157, *Glycine max*  (2e-29, 42%) | EFN53135  (9e-36, 44%) | EDO96210  (1e-32, 41%) |
|  | 06719  KA130269 | 740 bp  8.1/kb | EAL72763, *Dictyostelium discoideum* (5e-26, 45%) | EFN55091  (1e-21, 33%) | EDP07756  (8e-19, 31%) |
|  | 07125  KA130710 | 837 bp  3.6/kb | EDQ91872, *Monosiga brevicollis*  (1e-51, 50%) | EFN53222  (6e-57, 52%) | EDP04121  (2e-57, 54%) |
|  | 09075  KA132810 | 591 bp  5.1/kb | EGG14344, *Dictyostelium fasciculatum* (2e-42, 48%) | EFN58398  (1e-28, 39%) | EDP02909  (1e-28, 38%) |
|  | 09830  KA133621 | 1,691 bp  27.2/kb | EGC40363, *Dictyostelium purpureum* (2e-54, 33%) | EFN53179  (3e-36, 28%) | EDP02909  (7e-39, 33%) |
|  | 10431  KA090016 | 1,395 bp  17.9/kb | EFW45932, *Capsaspora owczarzaki*  (7e-26, 24%) | EFN59136  (2e-14, 23%) | EDO99773  (2e-15, 23%) |
|  | 10719  KA090329 | 2,779 bp  132.8/kb | CCA26515, *Albugo laibachii*  (4e-67, 34%) | EFN53179  (4e-133, 45%) | EDP07756  (2e-47, 31%) |
|  | 11979  KA091700 | 830 bp  9.6/kb | EGZ08256, *Phytophthora sojae*  (3e-19, 41%) | EFN55003  (4e-21, 34%) | EDP02909  (3e-19, 34%) |
|  | 12459  KA092224 | 1,383 bp  28.9/kb | EFW45932, *Capsaspora owczarzaki*  (4e-53, 53%) | EFN53135  (1e-35, 37%) | EDP02909  (6e-45, 45%) |
|  | 13388  KA093227 | 1,301 bp  46.1/kb | EFW45932, *Capsaspora owczarzaki*  (4e-54, 37%) | EFN53135  (1e-36, 31%) | EDP02909  (3e-49, 34%) |
|  | 15979  KA096032 | 1,301 bp  3.8/kb | EEH57118, *Micromonas pusilla*  (5e-76, 61%) | EFN51572  (3e-92, 68%) | EDO98284  (9e-51, 67%) |
|  | 15990  KA096044 | 1,289 bp  48.9/kb | EFW45932, *Capsaspora owczarzaki*  (4e-41, 29%) | EFN51572  (6e-25, 25%) | EDP02909  (4e-29, 39%) |
|  | 16595  KA096702 | 1,512 bp  3.3/kb | XP_002270397, *Vitis vinifera*  (3e-14, 45%) | EFN55091  (6e-15, 49%) | EDP02909  (4e-12, 43%) |
|  | 17249  KA097408 | 671 bp  14.9/kb | EFW45932, *Capsaspora owczarzaki*  (9e-16, 50%) | EFN58775  (3e-14, 45%) | EDP02909  (5e-11, 32%) |
|  | 21577  KA101935 | 698 bp  4.3/kb | AED96269, *Arabidopsis thaliana*  (8e-17, 58%) | EFN55091  (1e-14, 40%) | EDP02909  (2e-12, 36%) |
|  | 22750  KA103200 | 1,336 bp  6.0/kb | EDQ55167, *Physcomitrella patens* ssp. patens (4e-29, 48%) | EFN55090  (5e-16, 32%) | EDO96210  (1e-29, 56%) |
|  | 22904  KA103365 | 1,919 bp  8.9/kb | EFJ44628, *Volvox carteri f. nagariensis* (3e-85, 49%) | EFN50715  (1e-94, 53%) | EDO97711  (4e-32, 50%) |
|  | 23692  KA104213 | 2,100 bp  30.5/kb | EFW45932, *Capsaspora owczarzaki*  (3e-23, 26%) | EFN53179  (1e-12, 22%) | EDP06016  (3e-17, 23%) |
|  | 27786  KA108066 | 912 bp  5.5/kb | EFJ48316, *Volvox carteri f. nagariensis* (1e-26, 54%) | EFN58775  (3e-21, 36%) | EDP02909  (5e-25, 42%) |
|  | 28230  KA108544 | 545 bp  33.0/kb | BAJ88327, *Hordeum vulgare* ssp. vulgare (5e-13, 40%) | EFN55003  (1e-12, 43%) | EDP02909  (8e-12, 37%) |
|  | 31138  KA111709 | 3,272 bp  33.0/kb | EFW45932, *Capsaspora owczarzaki*  (1e-62, 45%) | EFN55091  (4e-36, 37%) | EDP07756  (2e-38, 37%) |
|  | 31324  KA111915 | 1,645 bp  17.0/kb | EFW45932, *Capsaspora owczarzaki*  (2e-80, 46%) | EFN53135  (2e-52, 41%) | EDO96210  (1e-52, 43%) |
|  | 36908  KA117988 | 1,028 bp  13.6/kb | EDQ85608, *Monosiga brevicollis*  (1e-28, 36%) | EFN55003  (1e-27, 36%) | EDP02909  (2e-26, 36%) |
|  | 40346  KA121398 | 621 bp  3.2/kb | EDQ51053, *Physcomitrella patens* ssp. patens (2e-17, 49%) | EFN53222  (1e-18, 48%) | EDP04121  (5e-16, 46%) |
|  | 41302  KA122438 | 900 bp  2.2/kb | EFC42872, *Naegleria gruberi*  (8e-31, 39%) | EFN54244  (1e-30, 50%) | EDP00679  (4e-24, 48%) |
|  | 43006  KA124318 | 1,795 bp  5.0/kb | EGG18935, *Dictyostelium fasciculatum* (1e-23, 31%) | EFN51572  (1e-12, 22%) | EDP06016  (2e-12, 23%) |
|  | 43986  KA125399 | 2,107 bp  3.8/kb | EFW47465, *Capsaspora owczarzaki*  (3e-18, 47%) | EFN54244  (5e-21, 32%) | EDP07607  (9e-22, 53%) |
|  | 46376  KA127986 | 812 bp  6.2/kb | EFW45932, *Capsaspora owczarzaki*  (1e-37, 52%) | EFN58775  (5e-25, 40%) | EDP02909  (4e-29, 45%) |

## Supplementary Table 17 – Machine-assembled contigs related to autophagy in the *B. braunii* Showa transcriptome

Coverage indicated in reads per thousand bases of the curated contig. Accession numbers refer to the GenBank Transcriptome Shotgun Assembly Sequence Database. Best BlastX hit with cutoff at 1e-5. GenBank: excluding *Ch. variabilis* and *C. reinhardtii*, shown separately. *, curated contig that may have originated from a transcript of a *B. braunii* cohabitant organism. NF, not found.

| **Enzyme (symbol)** | **Contig ID,**  **Accession** | **Contig length, coverage** | **Best BlastX hit in** | | |
| --- | --- | --- | --- | --- | --- |
|  |  |  | **GenBank** | ***Chlorella variabilis*** | ***Chlamydomonas reinhardtii*** |
| 5'-AMP-activated protein kinase, catalytic alpha subunit (AMPK), E.C. 2.7.11.11 | 03936  KA120421 | 1,039 bp  6.7/kb | EFJ50877, *Volvox carteri f. nagariensis* (2e-81, 54%) | EFN57456  (4e-83, 48%) | EDO96994  (1e-85, 53%) |
|  | 14669  KA094619 | 1,130 bp  9.7/kb | EFJ41434, *Volvox carteri f. nagariensis* (2e-137, 76%) | EFN56161  (2e-145, 78%) | EDO99409  (3e-120, 67%) |
|  | 17104  KA097252 | 919 bp  5.4/kb | EDQ77518, *Physcomitrella patens* ssp. patens (1e-15, 29%) | EFN56161  (1e-19, 31%) | EDO99409  (3e-15, 45%) |
|  | 37122  KA118226 | 532 bp  3.8/kb | EFJ50877, *Volvox carteri f. nagariensis* (3e-28, 51%) | EFN56248  (7e-51, 58%) | EDO96994  (4e-33, 52%) |
| mTOR associated protein (GβL) | 10102  KA089659 | 1,252 bp  25.6/kb | EFJ46224, *Volvox carteri f. nagariensis* (4e-118, 75%) | EFN53592  (3e-131, 81%) | EDO98145  (5e-123, 75%) |
|  | 41377  KA122520 | 325 bp  6.2/kb | ABK24259, *Picea sitchensis*  (1e-35, 77%) | EFN53592  (8e-44, 77%) | EDO98145  (4e-38, 70%) |
| FKBP12-rapamycin complex-associated protein (mTOR) | 32008  KA112670 | 1,680 bp  13.7/kb | AAN72136, *Arabidopsis thaliana*  (5e-60, 56%) | EFN54447  (2e-30, 44%) | ABB13529  (2e-58, 54%) |
|  | 44259  KA125700 | 767 bp  14.3/kb | EFJ44271, *Volvox carteri f. nagariensis* (1e-130, 84%) | EFN54447  (1e-26, 68%) | ABB13529  (5e-136, 84%) |
| unc51-like kinase (ATG1), E.C. 2.7.11.1 | 13068  KA092884 | 1,459 bp  10.3/kb | EDQ59728, *Physcomitrella patens* ssp. patens (8e-66, 58%) | EFN52208  (3e-35, 47%) | EDP02226  (2e-68, 58%) |
|  | 44480  KA125945 | 887 bp  4.5/kb | EFJ50877, *Volvox carteri f. nagariensis* (4e-35, 50%) | EFN56248  (2e-37, 39%) | EDO96994  (7e-38, 52%) |
| Beclin 1 (VPS30, ATG6) | 15449  KA095459 | 1,408 bp  6.5/kb | EDQ73282, *Physcomitrella patens* ssp. patens (2e-40, 45%) | NF | EDP09243  (7e-44, 43%) |
| Phosphatidylinositol 3-kinase (VPS34),  E.C. 2.7.1.137 | 37250  KA118368 | 524 bp  5.7/kb | EFJ45030, *Volvox carteri f. nagariensis* (1e-59, 62%) | EFN58631  (5e-16, 33%) | EDP09369  (5e-61, 59%) |
|  | 12122  KA091857 | 1,205 bp  24.9/kb | CAC81902, *Oryza sativa*  (8e-66, 60%) | EFN58631  (1e-66, 60%) | EDP05127  (3e-65, 58%) |
|  | 43969  KA125380 | 1,527 bp  5.9/kb | EER91000, *Sorghum bicolor*  (8e-39, 51%) | EFN54838  (9e-25, 66%) | EDP07180  (3e-58, 72%) |
|  | 44151  KA125583 | 1,776 bp  5.6/kb | EFJ51516, *Volvox carteri f. nagariensis* (2e-63, 60%) | EFN58631  (2e-58, 53%) | EDP05127  (1e-66, 62%) |
|  | 44259  KA125700 | 767 bp  14.3/kb | EFJ44271, *Volvox carteri f. nagariensis* (1e-130, 84%) | EFN54447  (1e-26, 68%) | ABB13529  (5e-136, 84%) |
| Autophagy-related protein 8 (ATG8) | 06183  KA129698 | 438 bp  6.8/kb | NF | EFN52105  (2e-07, 64%) | EDO98830  (7e-09, 44%) |
|  | 33285  KA114070 | 771 bp  204.9/kb | EDQ77980, *Physcomitrella patens* ssp. patens (3e-68, 90%) | EFN52105  (2e-73, 87%) | EDO98830  (5e-74, 85%) |
|  | 38760*  KA119908 | 385 bp  13.0/kb | EHK45493, *Trichoderma reesei*  (8e-71, 100%) | EFN52105  (3e-57, 80%) | EDO98830  (2e-60, 83%) |
| Autophagy-related protein 4 (ATG4),  E.C. 3.4.22.- | 41651  KA122818 | 922 bp  3.3/kb | EDQ76938, *Physcomitrella patens* ssp. patens (1e-27, 37%) | EFN56996  (1e-23, 41%) | EDP05495  (7e-30, 54%) |
| Autophagy-related protein 7 (ATG7) | 29233  KA109639 | 781 bp  6.4/kb | CAK37977, *Aspergillus niger*  (1e-49, 54%) | EFN52000  (4e-53, 63%) | EDP06047  (3e-43, 53%) |
| Autophagy-related protein 7 (ATG10) | NF |  |  |  |  |
| Autophagy-related protein 3 (ATG3) | 12569  KA092343 | 773 bp  6.5/kb | EFJ46364, *Volvox carteri f. nagariensis* (9e-40, 41%) | EFN54110  (3e-43, 67%) | EDP07491  (9e-43, 75%) |
|  | 45818*  KA127374 | 464 bp  15.1/kb | EFI94380*, Schizophyllum commune*  (6e-50, 73%) | EFN54110  (1e-19, 38%) | EDP07491  (5e-19, 36%) |
| Autophagy-related protein 12 (ATG12) | 20040  KA100304 | 334 bp  12.0/kb | EGF78859, *Batrachochytrium dendrobatidis* (2e-08, 55%) | EFN51330  (1e-15, 58%) | EDO96875  (1e-13, 50%) |
|  | 40768  KA121854 | 300 bp  16.7/kb | CBY20570, *Oikopleura dioica*  (4e-07, 47%) | EFN51330  (2e-08, 39%) | NF |
| Autophagy-related protein 5 (ATG5) | 40939  KA122041 | 1,721 bp  4.6/kb | ABR17459, *Picea sitchensis*  (5e-32, 45%) | EFN59373  (3e-33, 43%) | EDP03681  (1e-33, 43%) |
